# MOF-Derived PdCo and PdMn Systems as Versatile Catalysts in Alkyne Semihydrogenation

**DOI:** 10.1021/acscatal.4c07149

**Published:** 2025-04-18

**Authors:** Jordan Santiago Martinez, Luigi Carpisassi, Gonzalo Egea, Jaime Mazarío, Christian Wittee Lopes, Carmen Mora-Moreno, Susana Trasobares, Luigi Vaccaro, Jose Juan Calvino, Giovanni Agostini, Pascual Oña-Burgos

**Affiliations:** † Instituto de Tecnología Química, Universitat Politècnica de València-Consejo Superior de Investigaciones Científicas (UPV-CSIC), Avda. de los Naranjos s/n, Valencia 46022, Spain; ‡ Laboratory of Green S.O.C—Dipartimento di Chimica biologia e Biotecnologie, Università degli Studi di Perugia, Via Elce di Sotto 8, Perugia 06123, Italy; § Department of Chemistry, Federal University of Paraná (UFPR), Curitiba 81531-990, Brazil; ∥ División de Microscopía Electrónica de los Servicios Centralizados de Investigación Científica y Tecnológica de la Universidad de Cádiz (DME-UCA), Facultad de Ciencias, Universidad de Cádiz, Campus Río San Pedro S/N, Puerto Real, Cádiz 11510, Spain; ⊥ Departamento de Ciencia de los Materiales e Ingeniería Metalúrgica y Química Inorgánica, Facultad de Ciencias, Universidad de Cádiz, Campus Río San Pedro S/N, Puerto Real, Cádiz 11510, Spain; # ALBA Synchrotron Light Facility, Carrer de la Llum 2-26, Cerdanyola del Valles, Barcelona 08290, Spain

**Keywords:** MOF-derived, bimetallic nanoparticles, heterogeneous catalysis, alkyne semihydrogenation, nanomaterials characterization

## Abstract

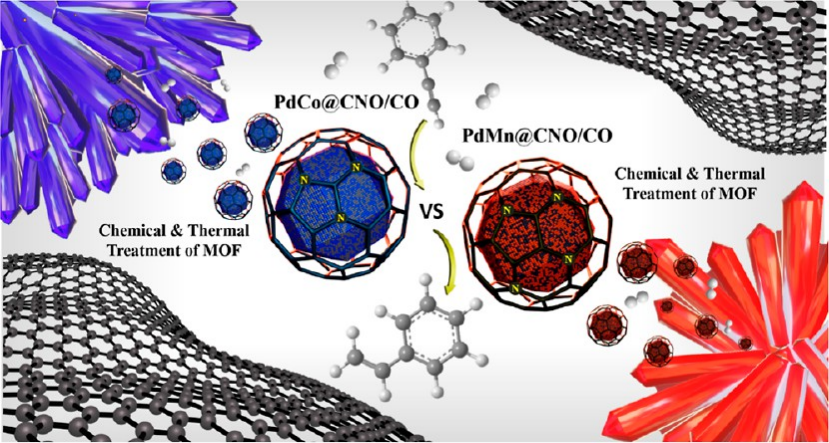

This study investigates the structure and catalytic properties of bimetallic nanocomposites derived from PdCo- and PdMn-based metal–organic frameworks. These materials, synthesized via chemical (Q) and thermal treatments (T), resulted in **PdCo-QT** and **PdMn-QT** catalysts containing Pd-based nanoparticles modified with Co or Mn and supported on N-doped carbon. Detailed characterization techniques confirm these complex structures, including high-resolution transmission electron microscopy, scanning transmission electron microscopy energy-dispersive X-ray spectroscopy, X-ray diffraction, X-ray photoelectron spectroscopy, and X-ray absorption spectroscopy. The catalytic performances of these materials were evaluated for the selective semihydrogenation of phenylacetylene and 4-octyne under soft conditions (1 H_2_ bar, room temperature) in batch reactors, demonstrating very high selectivity (≥95 mol %) toward alkenes at high conversion levels (≥94 mol %). Moreover, they displayed significant stability after five catalytic cycles with minimal leaching and highly competitive values of alkyne productivity in the semihydrogenation of phenylacetylene. The study also explored the potential of these catalysts in continuous gas-phase reactions, where **PdCo-QT** demonstrated remarkable catalytic activity and selectivity with a high gas hourly space velocity.

## Introduction

The semihydrogenation of alkynes to alkenes is a crucial process in the industrial purification of olefin streams. This process reduces the frequent presence of alkyne compounds as byproducts to the parts per million (ppm) range. Moreover, the production of substituted alkenes through the selective semihydrogenation of acetylenic compounds plays a vital role in the industrial production of polymer, pharmaceutical, and fragrance intermediates.^1−3^

An efficient semihydrogenation catalyst must strike a balance between preventing overhydrogenation of the formed alkene and maintaining a suitable reaction rate. Typically, alkyne and alkene adsorption on the catalyst surface is decisive in product selectivity. For high selectivity in alkyne hydrogenation to the alkene, it is essential that the desorption energy barrier of the alkene’s π-bond is lower than that required for further hydrogenation. This ensures that the alkene product is released from the catalyst surface before being overhydrogenated, thus maintaining selectivity.^4,5^

Pd-based catalysts dominate alkyne semihydrogenation because they offer a combination of high activity, tunable selectivity and stability, and the ability to operate under mild conditions.^6,7^ Usually, Pd-based catalysts have an undesired tendency to overhydrogenation and polymerization when applied in alkyne semihydrogenation. In fact, due to the strong adsorption of alkenes on large Pd atomic arrangements,^8^ and the relatively low activation barrier of alkene hydrogenation over Pd nanoparticles, Pd catalysts are highly active but only selective if they are poisoned.^4,9,10^ For instance, they are modified by the addition of Ag^11^ or Pb (Lindlar Pd catalyst)^12^ for their use in several commercial semihydrogenation processes. Nevertheless, these industrially relevant Pd-based catalysts have several disadvantages, such as containing environmentally unfriendly lead, low alkene selectivity in the hydrogenation of terminal alkynes, and low stability.^7^

The alternatives preferred in the literature to design highly selective heterogeneous catalysts for alkyne semihydrogenation include creating metal–organic interfaces,^13−16^ controlling Pd ensemble formation, downsizing, and local environment through support engineering,^17−22^ or adding other elements such as Cu,^23−25^ Ni,^26^ Zn,^27,28^ Ga,^29,30^ In,^31−33^ Bi,^34^ S,^35^ C,^36−38^ B,^39^ or Ag.^40,41^ These strategies work most of the time by preventing the emergence of unselective subsurface β-hydride species on Pd and isolating Pd sites to alter the intermediate adsorption behaviors.

In the pursuit of novel approaches to Pd-based bimetallic systems, we previously optimized a soft chemical treatment using aniline and H_2_ to transform a bimetallic metal–organic framework (MOF) with [Fe_3_(μ_3_-O)(-COO)_6_] and *trans*-[PdCl_2_(PDC)_2_] (PDC: pyridine-3,5-dicarboxylate) subunits into a bimetallic nanocomposite containing a N-doped carbon.^42,43^ Combining this chemical pretreatment with a subsequent pyrolysis results in better preservation of the MOF features, such as high metal dispersion at very high loadings as well as a large surface area. These efforts, along with those from other authors, have shown the value of MOF-mediated synthesis to produce a new generation of catalysts with high porosity, long-range order, high dispersions for high metal loadings, unprecedented stoichiometries, functionalized carbons, or encapsulated metal nanoparticles.^44,45^

This way, the combination of our soft chemical and the pyrolytic treatments crystallized in a successful catalyst based on PdIn nanoparticles supported on N-doped graphitic carbon, derived from a PdIn-MOF. Remarkably, despite the high metal loading (ca. 50 wt %), we achieved a good nanoparticle size distribution, both in terms of average and narrow size range. This precise control, along with the presence of nitrogen in the support, led to an intermetallic system that preserved a high catalytic activity while effectively inhibiting the overhydrogenation of alkynes to alkanes.^33^

**Scheme 1:**
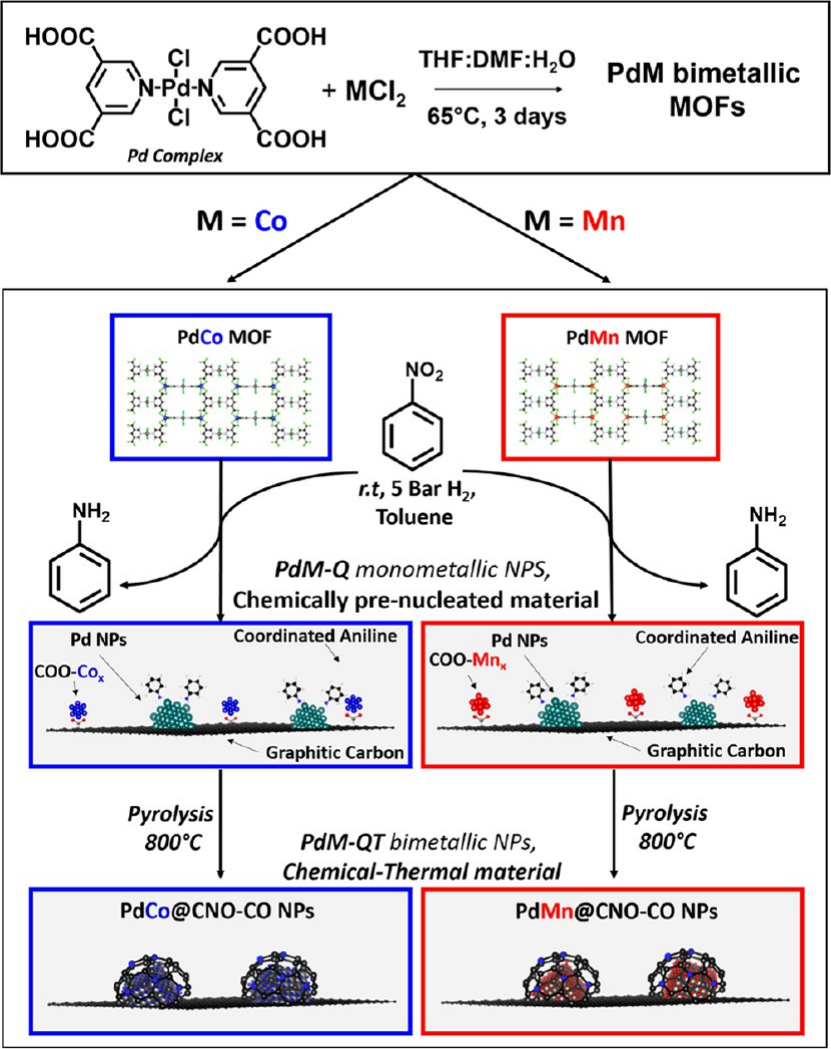
Synthetic Routes Described in This Work to Achieve the Final Catalytic Composites

In this work, we aimed to replace indium (In) with less expensive and more abundant transition metals, such as Mn and Co. We started by synthesizing new bimetallic PdCo and PdMn-MOFs incorporating the structural subunits. Then, these materials were submitted to our previously reported soft chemical method followed by a pyrolysis treatment. The result is again two materials based on PdCo and PdMn nanoparticles with optimized dispersion supported on high-surface-area nitrogen-doped graphitic carbon (see Scheme 1). The performance of these materials in the semihydrogenation of phenylacetylene surpasses that reported for the PdIn system. Furthermore, its catalytic activity could be intensified by working in continuous flow and even transposed to internal alkyne (4-octyne) semihydrogenation.

## Materials and Methods

### Catalyst Preparation

#### Preparation of PdM-MOFs (M = Co or Mn)


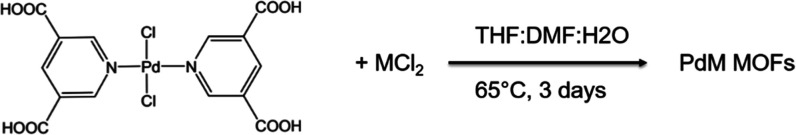
Typically, a mixture of 0.23 mmol of the Pd complex and 0.53 mmol of MCl_*X*_ (M: Co/Mn) was dissolved under vigorous magnetic stirring in 56 mL of a THF/DMF/H_2_O solvent mixture (28 mL/21 mL/7 mL). The resulting solution was evenly poured into 10 scintillation vials (5.6 mL in each). Then, the vials were sealed and placed in an oven at 65 °C for 72 h. After cooling the vials at room temperature, the resulting crystals were recovered by vacuum filtration, washed several times with acetone, and dried under vacuum.

#### Chemical Treatment: Preparation of PdM-Q (M = Co or Mn)

The **PdM MOF** (400 mg) was placed into a 300 mL hydrogenation reactor with a solution of 80 mmol of nitrobenzene and 80 mL of toluene. The system was sealed and pressurized at 5 H_2_ bar at room temperature. After 24 h of vigorous magnetic stirring, the resulting dark solution was filtrated under vacuum to recover the material. The material was then washed multiple times with methanol and activated at 300 °C under vacuum for 6 h.

#### Chemical-Thermal Treatment: Preparation of **PdM-QT** (M = Co or Mn)

In order to synthesize **PdM-QT** materials, a pyrolytic thermal treatment was applied to **PdM-Q**. Accordingly, 200 mg of **PdM-Q** (before the activation step under vacuum) were pyrolyzed in a tubular fixed-bed reactor under N_2_ flow (20 mL·min^–1^) at 800 °C for 2 h (ramp 25 °C·min^–1^). Then, the material was cooled to room temperature with a higher N_2_ flow (40 mL·min^–1^).

#### Characterization Techniques

Below, the techniques employed to characterize the synthesized materials are described.

#### Elemental Analysis (E.A.)

The nitrogen, carbon, and hydrogen (N, C, H) contents were determined with a CNHS EA3000 elemental analyzer from Eurovector (calibrated with Sulphanilamide from Elemental Microanalysis company).

#### Inductively Coupled Plasma Spectrometry

iCAP PRO XP inductively coupled plasma atomic emission spectrometer (ICP–AES) was used to determine the metal content of the different materials. Prior to analyses, 20 mg of the sample was digested in a mixture of 98% sulfuric acid (4 mL), with a few drops of hydrogen peroxide, at 100 °C, and vigorously stirred for 24 h.

#### Thermogravimetric Analysis

This technique has been used to study the decomposition and desorption of molecules from solid materials with temperature. Measurements were conducted in a Jupiter STA 449F3 apparatus from NETZSCH. The heating rate was 25 °C·min^–1^ in an air or N_2_ stream, and the temperature ranged from 25 to 800 °C.

#### Powder X-ray Diffraction

X-ray diffraction (XRD) was used to identify the atomic periodical structure of the solids, resulting in different crystalline phases. The X-ray diffraction measurements were acquired in Bragg–Brentano geometry, using a CUBIX diffractometer from PANalytical operating at 40 kV and 35 mA, and equipped with a PANalytical X’Celerator detector. X-ray radiation from the Cu Kα source was used in the range of 2 to 90° (2θ) with a step of 0.020° (2θ). Experimental diffractograms were compared against the PDF2 database (codes in parentheses).

Equations used to evaluate the crystallite size^1^ and the lattice parameters^2,3^ are available in the Supporting Information.

#### Gas Adsorption Measurements

The relationship between adsorbed gas molecules and the partial pressure at a constant temperature was registered in adsorption isotherms.

Specifically, nitrogen adsorption isotherms (for BET area) were recorded for PdM-MOF-derived materials in an ASAP 2420 apparatus from Micrometrics at −196 °C from 0 to 1 relative pressure (*P*/*P*_0_). First, 150 mg of pelletized sample (0.2–0.4 mm) were degassed at 400 °C at ≈5 × 10^–6^ bar overnight. The BET surface area was calculated by using the Brunauer–Emmet–Teller equation fulfilling the criterion by Rouquerol et al.^46^ and the micropore volume was calculated by the t-plot method.

#### TEM–STEM Characterization

TEM/STEM measurements were performed to obtain information about the structure (HR-TEM and HR-STEM-HAADF) and composition of the materials (STEM–XEDS and STEM-EELS) with a very high spatial resolution. The samples were prepared for analysis by placing a drop of a suspension of the corresponding powders in ethanol onto a lacey-carbon-coated Cu grid (300 mesh).

HR-TEM images were recorded using a JEOL JEM2100F operating at 200 kV. The analysis in the frequency space of HR-TEM images was carried out using Gatan Digital Micrograph software. The crystalline phases were identified by comparing quantitative measurements obtained from FFTs (*d*_*hkl*_ values and interplanar angles) of selected areas of the images to those made on simulated electron diffraction patterns.

Scanning transmission electron microscopy (STEM) studies were performed on a double Aberration-Corrected FEI Titan Cubed Themis 60–300, available at the DMEUCA node of the Spanish Unique Infrastructure (ICTS) on Electron Microscopy of Materials ELECMI. Particle size distribution was obtained by fitting nanoparticle size frequency plots with a Gaussian curve using at least 200 size measurements. Unless otherwise specified, the sizes estimated by directly measuring the EM images correspond to the metallic core, excluding the contribution of the carbonaceous layers. ImageJ software was used to estimate the individual nanoparticle sizes. The instrument is also equipped with a high energy resolution Gatan GIF Quantum ERS/966 electron energy loss (EELS) spectrometer, as well as a high efficiency X-ray energy-dispersive spectroscopy (XEDS) Super X-G2 system. These allowed us to simultaneously combine two spectroscopic signals with high-resolution STEM images. XEDS and EELS experiments were performed by working in the spectrum imaging (SI) mode,^47^ allowing for the correlation of analytical and structural information on the selected regions of the material under study. In this technique, the spectroscopy and HAADF signals were collected simultaneously, while the electron beam was scanned across the selected area of the sample. STEM–XEDS experiments were recorded using the 4-SDD detectors of the Super X-G2 system of the microscope using a beam current of 120 pA and a dwell time per pixel of 100 μs. X-EDS maps were obtained by analyzing the C–K, O–K, Pd–L, Co–K, or Mn–K lines. To improve visualization, the elemental maps were postfiltered using a Gaussian blur of 0.8, as provided in the Velox software. EELS data was acquired using the DUAL EELS acquisition mode, which allows acquisition of the core loss spectrum with the accompanying low loss spectrum. A series of EELS spectra (core loss and low loss with an acquisition time of 50 and 0.1 ms, respectively) were acquired by using an energy dispersion of 0.25 eV and 50 pA probe current. Chemical information from the samples was obtained by acquiring the C–K, N–K, Pd–M, Co–M, Mn–M, and O–K EELS signals. The low-loss spectrum was used to realign and calibrate the acquired spectra, and the chemical maps were obtained after quantifying the EELS data using standard methods. To identify the different phases present in the sample, EELS data was denoised using principal component analysis (PCA), and then, the individual spectral responses from the sample were obtained using the independent component analysis (ICA) method, both available at the Hyperspy open-source program^48,49^ ICA is a mathematical treatment that separates a multivariate signal into additive subcomponents, assuming that these subcomponents are statistically independent.

#### Field Emission Scanning Electron Microscopy

Field emission scanning electron microscopy (FESEM) was used to characterize morphology and average composition (at the micrometer scale) of the parent PdCo and PdMn-MOFs, serving as precursors for the catalysts used in this work. Powder samples were prepared on a sample holder with S4 double-sided adhesive tape for dispersion. The images were acquired using a Zeiss Ultra 55 microscope at 1.0 kV. This microscope also had an EDS X-Max 80 detector operating at 10.0 kV.

#### Raman Spectroscopy

Raman spectroscopy allowed us to gain insights into the structural characteristics of the carbonaceous parts of the nanocomposites. A LabRAM-HR Raman spectrometer (600 mm^–1^ grating, 100 mm entrance slit) coupled to a Peltier-cooled CCD detector and an Olympus BXFM optical microscope was used to acquire Raman spectra. The scattering was produced by excitation at 514 nm using a HeNe laser with 0.1 mW of excitation power on the samples. The laser beam was focused on the sample at 50× the microscope objective (numerical aperture = 0.5). Rayleigh scattering was removed by a holographic notch filter, and the Raman spectra were recorded between 200 and 2000 cm^–1^, with a resolution of 0.5 cm^–1^.

#### X-ray Absorption Spectroscopy

The energy dependence of X-ray absorption from the inner-shell electrons of the atoms provides valuable insights into the specific elements present in a sample, including their local coordination environment, oxidation state, and electronic structure. Therefore, X-ray absorption spectroscopy (XAS) experiments were conducted at the Pd K-edge (24350 eV), Co K-edge (7709 eV), and Mn K-edge (6539 eV) using the NOTOS beamline at the ALBA Spanish synchrotron facility in Cerdanyola del Vallès, Spain. The initial broad-spectrum beam was narrowed down with a water-cooled Si(111) double crystal monochromator, and unwanted harmonics were filtered out using two mirrors working at grazing incident equipped with Si and Rh strips for the low and high energy, respectively. The samples were blended with appropriate quantities of BN and then measured as self-supporting pellets with a carefully adjusted thickness to achieve an edge jump of approximately 1. The spectra were acquired in transmission mode by employing ionization chambers as detectors. Pd, Co, and Mn metal foils were employed as references for aligning the data; these were positioned between the I1 and I2 ionization chambers. Multiple spectra were gathered for each sample to ensure consistency and quality of the signal-to-noise ratio. The reduction of data and the extraction of the function χ(*k*) were accomplished using the IFEFFIT package.^50^ A corefinement fit was applied to Pd, Co, and Mn edges for the samples subjected to thermal (**PdM-T**) and chemical-thermal (**PdM-QT**) treatments.

#### X-ray Photoelectron Spectroscopy

In X-ray photoelectron spectroscopy (XPS), X-rays eject electrons from inner atomic shells, raising them beyond the Fermi level (EF). Their kinetic energy is registered and transformed into the so-called binding energy (B.E.), which is element-specific and influenced by the oxidation state and chemical environment. As a result of the shallow escape depth of the photoemitted electrons, XPS is extremely sensitive to the surface layers of a material, typically analyzing only the top few nanometers (<10 nm).

In our case, XPS analysis was carried out using a SPECS spectrometer equipped with a Phoibos 150 MCD-9 multichannel analyzer using a nonmonochromatic Mg Kα radiation (50 W, 1253.6 eV). The spectrometer was calibrated by measurements of core level signals from Cu and Ag foils, and the spectrometer BE scale was adjusted so that BEs of reference peaks agreed with the recommended values.^51^ Core-level spectra of powdered samples (∼10 mg) were recorded by loading them onto a SPECS stainless-steel sample holder, acquired with constant pass energy values at 30 eV, using a 7 × 20 mm analysis area, at 25 °C, and under an operating pressure of 10^–9^ mbar. Intensities were corrected with a spectrometer transmission function. Curve fitting was performed with CasaXPS software, fixing the main contribution to the C 1s signal at 284.8 eV (Csp^3^: C–C, C–H). Shirley-type or U 2 Tougaard backgrounds were subtracted from the signals. Gaussian–Lorentzian curves were used to determine the B.E. of the different contributions for each of the element core levels, with a degree of asymmetry introduced in the C 1s component corresponding to graphitic carbon, and in the Pd 3d components corresponding to Pd^0^. These asymmetries were based on the analyses of a reference graphitic carbon sample and an in situ-reduced reference Pd-based sample (**Pd-H**_**4**_**L-QT**, vide infra).

### Catalytic Tests and Stability

#### Liquid-Phase General Procedure

Reactions were carried out in a 12 mL glass microreactor equipped with a pressure gauge and a metallic probe for sample collection on a thermostatic hot plate equipped with a magnetic stirrer (1000 rpm). The alkyne (5 mmol) and the catalyst (substrate/Pd: 323/1 molar ratio, i.e., 5.9 mg in the case of **PdCo-QT**) were mixed in ethanol (5 mL). The reactor was then pressurized at the desired hydrogen pressure (1 bar), and the pressure was maintained throughout the experiment. Once the reaction was finished, the catalyst was removed by vacuum filtration. The products were identified and analyzed by gas chromatography (Agilent 7890A equipped with an HP5 column: 32 m, 0.25 mm/0.25 μm; and a FID detector). Reactant conversion and product quantification were determined from GC data using calibration curves, dodecane as the internal standard, and the equations presented in the Supporting Information (A–E).

#### Reusability Tests

**PdCo-QT** and **PdMn-QT** catalysts were recovered by centrifugation (8000 rpm for 20 min), washed three times with ethanol (5 mL each), and dried overnight under vacuum. Then, catalytic experiments and analytical protocols followed the above-described methodologies for typical tests with fresh catalysts.

#### Leaching Tests (Catalyst Filtration)

The leaching of active species was studied by filtering the reaction mixture with 0.45 μm Nylon filters after stopping the reaction at 3 h. This operation was repeated twice. On one hand, the filtrate was returned to a clean reactor, and the standard procedure for catalytic tests was followed. On the other hand, the filtrate was analyzed by ICP to identify any metal species.

#### Gas-Phase General Procedure

Catalytic experiments were performed in a 1 cm diameter quartz tubular fixed-bed continuous reactor. The temperature (150 °C) was controlled by an ultrathin platinum thermocouple introduced in a separate space in the quartz tube. Phenylacetylene was introduced in a glass vessel equipped with a bubbling system, refrigerated at 19 °C and carried toward the catalyst bed using a controlled N_2_ flow from 0 to 30 mL·min^–1^ as a range and 1 mL·min^–1^ as a step (Figure S1). To calculate the reagent quantity which reached the catalytic bed, the Antoine equation was used, resulting in 0.56 mmol of phenylacetylene/100 mL of N_2_ (or 1.10 mmol of 4-octyne/100 mL). The H_2_ is directly carried to the reactor inlet by a flow controller inside the tubular reactor. The pressure has been maintained at 1 atm. The catalytic bed was prepared with 5 mg of **PdCo-QT** and **PdMn-QT**. The catalyst was physically mixed with SiC and normalized to 1.2 mL of volume. The reactor outlet is connected to a GC-FID (Agilent 7890A equipped with an HP5 column 32 m, 0.25 mm/0.25 μm). Conversion and product quantification were determined from GC data using calibration curves, similar to liquid phase quantification. Additionally, GHSV was calculated using equation E in the Supporting Information.

## Results and Discussion

### Catalyst Characterization

First, we report the synthesis of new PdCo and PdMn-based bimetallic MOF. They are constituted by the subunits [M^1^_3_(μ_3_-O)(–COO)_6_] and *trans*-[PdCl_2_(PDC)_2_], as in the PdIn-based MOF previously reported by Brastos et al.^52^ The reader is guided to the Supporting Information for the corresponding characterization (Figures S4 and S5 and Table S1). Then, several nanocomposites were prepared from these new **PdCo-MOF** and **PdMn-MOF** by a combination of chemical (**PdCo-Q** and **PdMn-Q**) and thermal treatments (**PdCo-QT** and **PdMn-QT**). The chemical compositions of these materials are reported in Tables 1 and S2. A modification of the PdM ratio throughout the chemical treatment is observed in all cases. Concretely, there is an increase in the Pd/Co and Pd/Mn molar ratio from 1:1.4 and 1:1.7 in the MOFs up to 1:1 and 1:1.3 in the chemically treated materials, respectively. Oppositely, the chemical compositions of the **PdCo-QT** and **PdMn-QT** material presented in Table 1 confirmed that the thermal treatment does not involve any further changes in terms of metal molar ratio (**PdCo-QT**: 1:1 and **PdMn-QT** 1:1.4). After chemical and thermal modifications, **PdCo-QT** and **PdMn-QT** materials exhibit 118 and 133 m^2^·g^–1^ BET surface area.

**Table 1:** Chemical Compositions of the **PdCo-QT** and **PdMn-QT** Materials

material	Pd wt %^a^	M	M wt %^a^	Pd/M ratio (mol %)	N wt %^b^	C wt %^b^	H wt %^b^	cBET surface area (m^2^·g^–1^)	dpore size (Å)
**PdCo-QT**	27.9	Co	15.6	1:1	1.7	23.2	0.4	118	59
**PdMn-QT**	36.5	Mn	25.1	1:1.4	1.9	22.0	0.4	133	70

a Calculated by ICP.

b Calculated by EA.

c From N_2_-adsorption isotherm (BET method).

d From N_2_-desorption isotherm (BJH-plot method).

**Figure 1:**
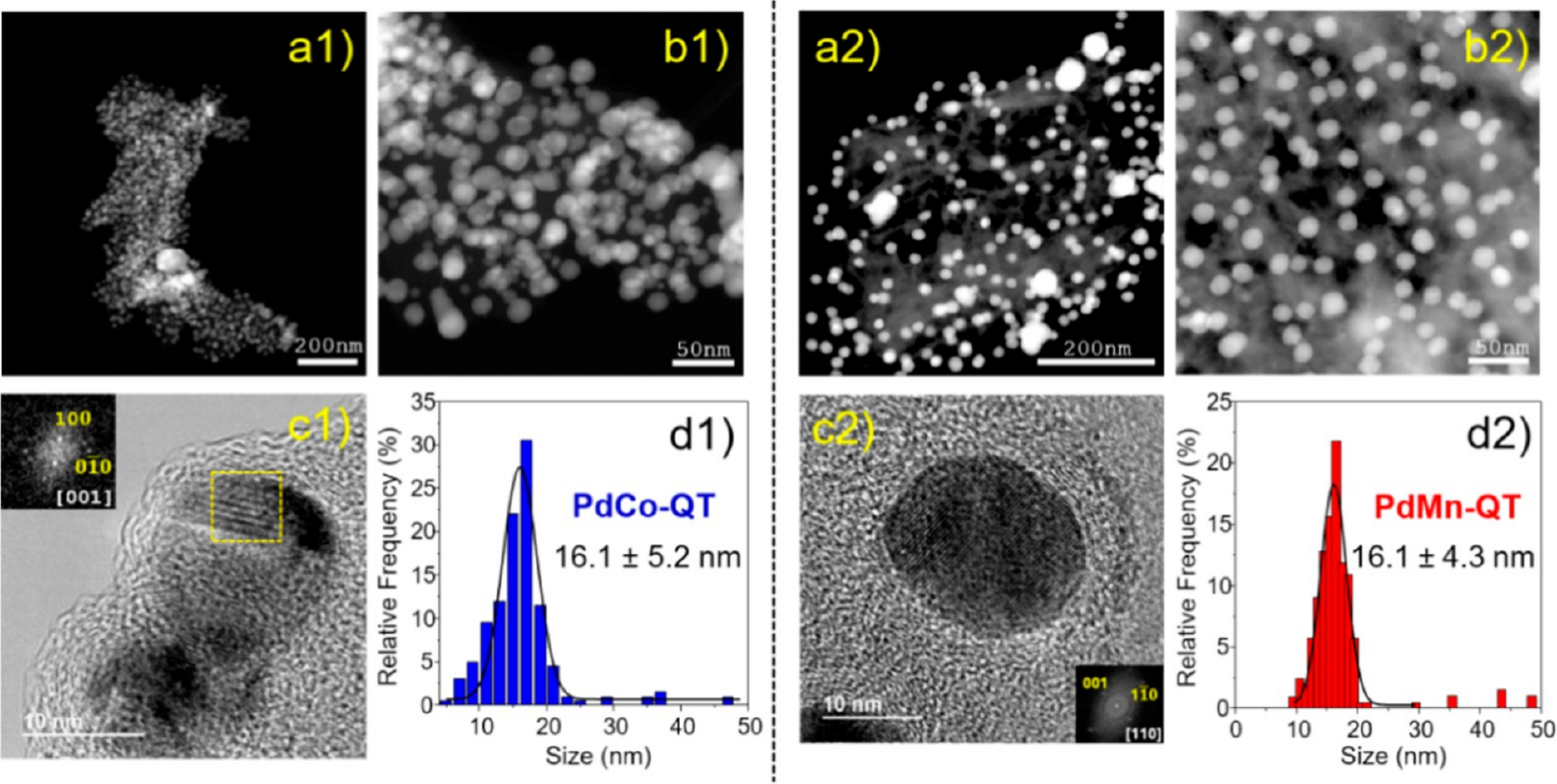
Electron microscopy characterization of (1) **PdCo-QT** (left panel) and (2) **PdMn-QT** (right panel). (a,b) Representative STEM-HAADF images of the **PdMn-QT** catalyst; (c) representative HR-TEM image of the **PdMn-QT** catalyst and the measured interplanar distances. FFTs of the HRTEM images, depicting reflections characteristic of the ordered phases, are shown as insets; (d) nanoparticle size distributions.

As well as in the previously described composites based on PdIn-materials,^33^ the mild conditions used during the chemical treatment facilitate a soft transformation of the parent MOFs. This chemical process does not lead to intermetallic or alloy species formation. Still, it generates a combination of two types of NPs, a first one consisting of Pd and a second one consisting of an oxide of the first–row transition metal (Figure S6 and S7). The harsher conditions provided by the pyrolysis step drive the mixing of the two metals into the desired bimetallic NPs. Thus, the combination of chemical and thermal processes of the **PdCo** and **PdMn-MOF** precursors results in two new composites: **PdCo-QT** and **PdMn-QT** catalysts, which have been successfully reproduced (see Figure S15). According to the electron microscopy analyses, these materials exhibit the presence of supported metal nanoparticles with a narrow size distribution of average size 16.1 ± 5.2 and 16.1 ± 4.3 nm for **PdCo-QT** and **PdMn-QT**, respectively (Figure 1).

**Figure 2:**
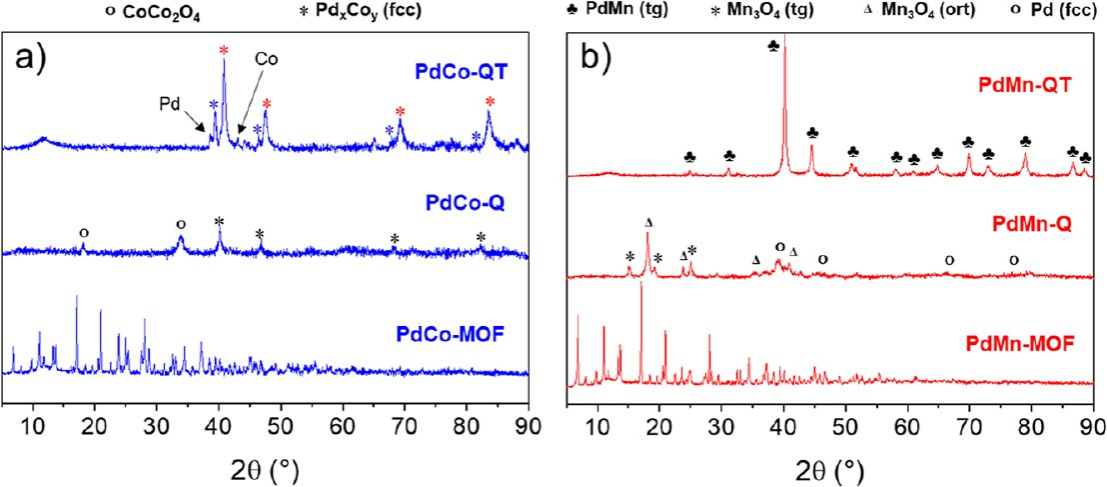
XRD patterns of PdM synthesized materials, (a) **PdCo** and (b) **PdMn**. Note: different colors for (*) indicate different compositions.

**Figure 3:**
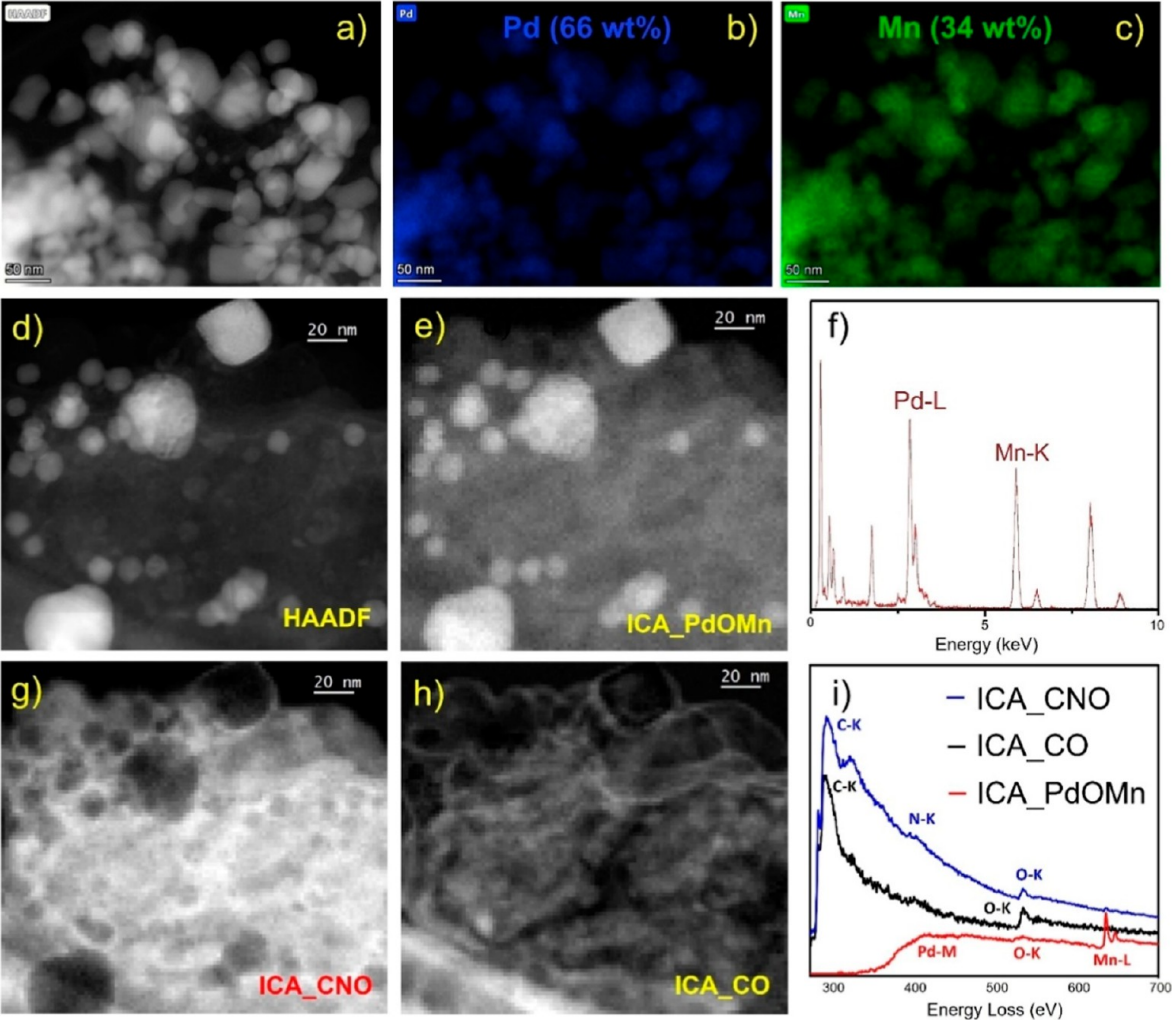
STEM–XEDS and STEM-EELS of **PdMn-QT** sample. (a) HAADF image and the corresponding elemental maps extracted from the STEM-SI-XEDS: (b) Pd and (c) Mn; and (f) an area representative XEDS spectrum. (d) HAADF image; and the images corresponding to three components of the ICA analysis of the whole set of STEM-EELS-SI data, (e,g,h). (i) EELS spectrum corresponding to the three independent components, a Pd–O–Mn, a C–N–O, and an external C–O component.

Remarkably, analyses from XRD (Figure 2), STEM XEDS, STEM-EELS (Figure 3), and HR-TEM (Figure 1) confirm the bimetallic nature of the nanoparticles in the PdM materials after thermal treatment (**PdM-QT**). In the first place, when examining the **PdMn-QT** material, STEM–X-EDS (Figure 3) reveals that Pd and Mn metals appear mixed in the nanoparticles. The relative composition of these NPs is 66 wt % Pd and 34 wt % Mn, which corresponds to an atomic ratio of 1:1. This composition aligns with the expected stoichiometry of a PdMn intermetallic phase. This result also agrees with those obtained in the STEM-EELS (Figure 3e,g–i) study, which shows the presence of an ICA corresponding to the intermetallic nanoparticles. As a reminder, the ICA method is a mathematical treatment that separates a multivariate signal into additive subcomponents, assuming that these subcomponents are statistically independent. In this way, it helps separate different spectral components that arise either from different physical processes (e.g., plasmon resonances) or material phases. This component also contains a tiny contribution of oxygen (Pd–Mn–O), which is related to the oxidation of Mn on the surface (plot in green in Figure 3i). Moreover, HR-TEM imaging and the corresponding Digital Diffractograms (Figure 1c2) reveal a diffraction pattern that does not correspond to fcc-type Pd–Mn solid solutions but matches specific zone-axis images characteristic of a PdMn intermetallic structure. An additional HR-HAADF-STEM study evidencing Pd and Mn ordering into a PdMn intermetallic can be found in the Supporting Information (Figure S13).

Furthermore, the analysis of the XRD pattern (Figure 2 and Table S4) reveals the presence of crystalline tetragonal Pd_1_Mn_1_ (CuAu type) nanoparticles with experimental cell parameters (*a* = *b* = 2.87 Å, *c* = 3.58 Å), in accordance with the JCPDS (01-071-9662) data. The pattern has been indexed, and the presence of (001), (100), (101), (110), (002), (111), (102), (200), (112), (201), (210), (211), (202), and (103) reflection planes at 24.9, 31.1, 40.2, 44.5, 50.9, 51.6, 60.9, 64,8, 69.9, 73.1, 79.0, 86.7, and 88.5°, respectively, has been observed. Additionally, the FFT analysis along the [110] zone axis of the HR-TEM image in Figure 1c2 confirms this interpretation.

**Figure 4:**
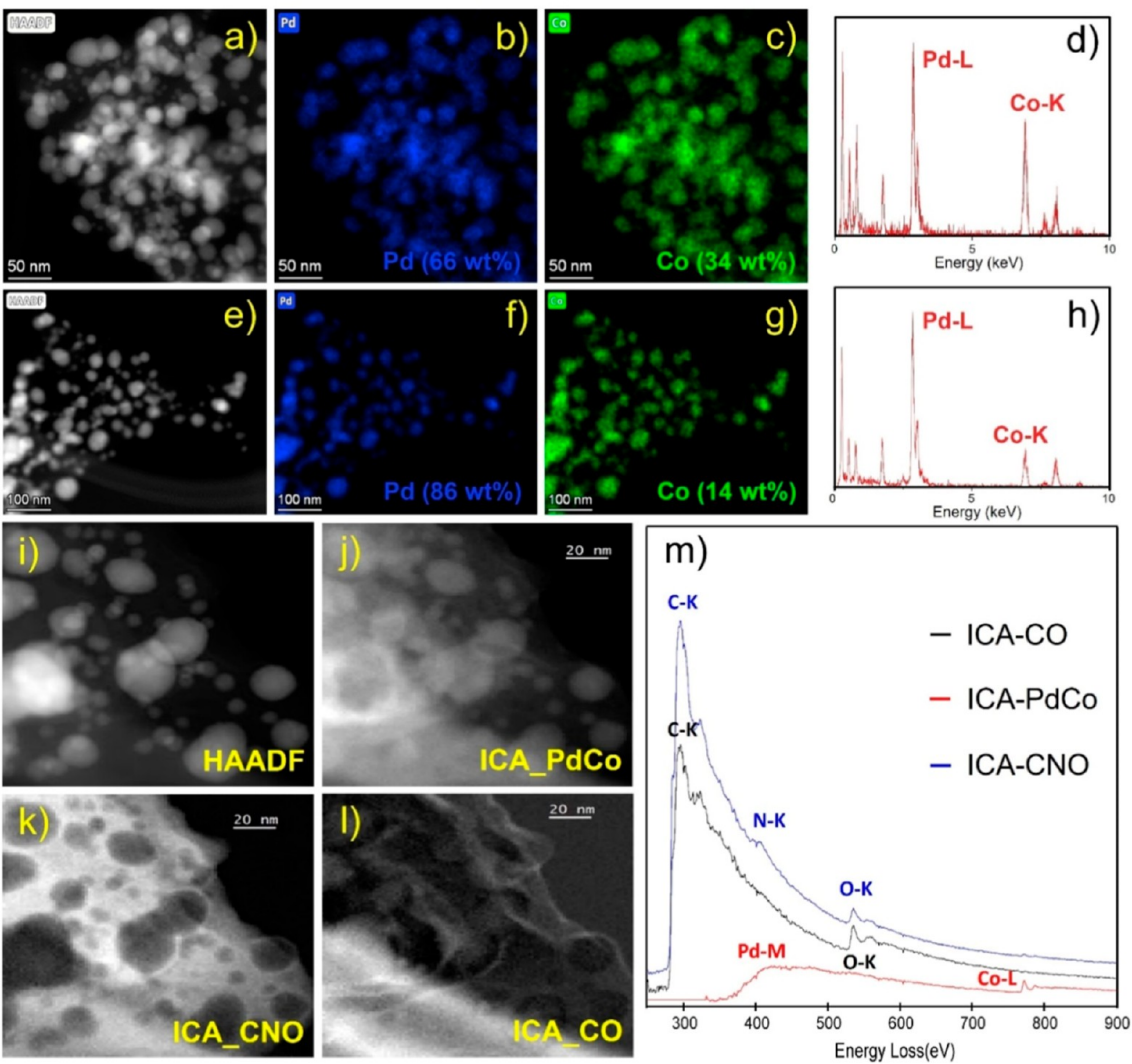
STEM–EDX and STEM-EELS of **PdCo-QT** sample. (a,e) HAADF images; (b,f) Pd and (c,g) Co elemental maps extracted from the STEM-SI-EDS, and (d,h) representative XEDS spectra. (i) a HAADF image; and the images corresponding to three components of the ICA analysis of the whole set of STEM-EELS-SI data: a (j–l). (m) EELS spectra corresponding to the three independent components: a Pd–Co component, a C–N–O component, and an external C–O component.

Similarly, STEM–XEDS analysis of **PdCo-QT** materials (Figure 4a–h) showed an intimate mixture, at the atomic scale, of Pd and Co metals, also confirmed by the presence of the PdCo component from the STEM-EELS study (Figure 4m). However, according to STEM–XEDS, two different bimetallic nanoparticle compositions were observed in this case. The **PdCo-QT** materials showed a first population consisting of 66 wt % of Pd and 34 wt % of Co and other population based on 86 wt % of Pd and 14 wt % of Co (Figure 4d,h).

These results agree with the XRD pattern, which suggests the presence of two fcc crystal systems (Figure 2). In particular, the XRD diagram indicates the presence of two patterns corresponding to shifted versions of that of Pd_1_Co_1_ fcc (JCPDS: 03-065-6075), with estimated average crystal sizes of 17 and 22 nm. The peaks corresponding to the (111), (200), (220), and (311) reflections of this phase appear in this case at the two following sets of diffraction angles: (40.9, 47.5, 69.3, and 83.6°) and (39.5, 46.4, 65.1, and 81.5°). Compared to the standard, a different nanoparticle composition can explain the pattern shifts. In this sense, the experimental lattice parameter has been calculated and compared to a theoretical plot of lattice parameter versus composition (Figure S9). PdCo fcc nanoparticles exhibit a lattice parameter of 3.75 Å, whereas our **PdCo-QT** material exhibits a phase close to Pd_2_Co (fcc structure) with a lattice constant at 3.83 Å and a Co-doped Pd (fcc) with a lattice constant at 3.94 Å (Table S4). Therefore, the PdCo fcc structures of the **PdCo-QT** materials can be estimated as Pd_0.7_Co_0.3_ and Pd_0.9_Co_0.1_ by simple interpolation. Additionally, FFT analysis along the [001] zone axis of the HR-TEM image in Figure 1c1 proves atomic ordering, distinguishing the observed phases from disordered solid solutions. Finally, monometallic Pd phase traces can also be observed in the XRD pattern.

Regarding the organic fraction in these composites, both **PdMn-QT** and **PdCo-QT** STEM EELS and STEM XEDS studies (Figures 3 and 4) reveal the presence of a double carbon layer surrounding the NPs, based on C–N–O and C–O according to the ICA component analyses. Additionally, Figure S10 presents a line scan at the exact location of Figure 3d. Note that the C–O component peaks at both sides of the Pd–O–Mn component. This profile does not reach zero in the intermediate positions, which indicates that the C–O layer fully covers the metallic core. On the other hand, the C–N–O component (blue line) increases intensity just outside the area corresponding to the metallic core, but it is more intense and extends beyond that of C–O. An attempt to measure the thickness distribution of the sum of both layers is also presented in Figures S11 and S12. As seen in our previous work, these two layers induce higher resistance against metal leaching.^33^ Additionally, in Figure S8 and Table S3, the Raman spectra of these materials show the typical graphitic carbon G band at 1589 cm^–1^.^53−55^ The ratio *I*_D_/*I*_G_ indicates that the order of the carbon is very low (**PdMn-QT**, *I*_D_/*I*_G_ = 0.86 and **PdCo-QT***I*_D_/*I*_G_ = 0.83). Also, typical bands from MnOx, and CoOx species are detected, suggesting surface oxidation due to ambient exposure (**PdMn-QT**: 642, 793 cm^–1^, and **PdCo-QT**: 673 cm^–1^). According to literature, the Raman signals at 642 and 793 cm^–1^ from **PdMn-QT** samples correspond to bands slightly shifted from those of tetragonal MnO_2_.^56^ Likewise, the signal at 673 cm^–1^ from **PdCo-QT** material is only slightly shifted to the A_1g_ transition of Co_3_O_4_.^57^ These observed band shifts are likely due to Pd–M–O_*X*,_ or Pd–O_*X*_–M (M = Co, Mn) interactions, as suggested by PdM EELS ICA analysis (Figures 3i and 4m).

**Figure 5:**
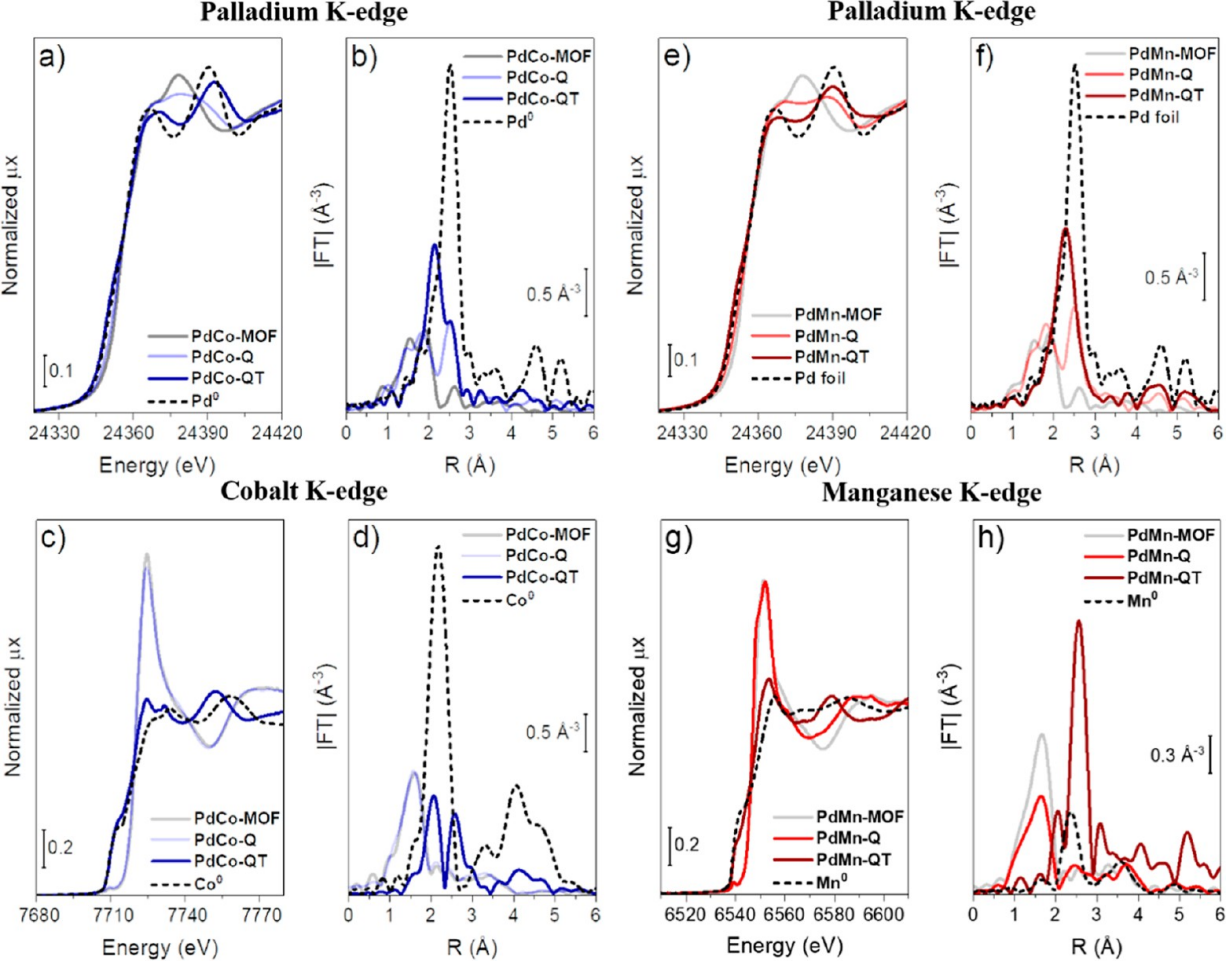
XANES spectra at the (a,e) Pd K, (c) Co K and (g) Mn–K-edges, and *k*^2^ weighted |FT| EXAFS spectra of (b,f) Pd, (d) Co, and (h) Mn data of MOF-derived PdCo and PdMn samples.

To strengthen the conclusions drawn from electron microscopy, XRD, and Raman analyses, XAS was also conducted to gather insights into the electronic properties and local environments of Pd, Mn, and Co atoms within the bulk of PdM materials (Figure 5). The Pd, Mn, and Co K-edge XANES spectra reveal the progression of Pd, Mn, and Co atoms from the PdM-MOF to the MOF-derived samples. Figure 5a shows the normalized Pd K-edge of the PdCo-based samples in different catalyst preparation steps. The spectrum of the **PdCo-MOF** sample (gray) presents the absorption edge located at the typical position for Pd^2+^ (∼24,354 eV), and the edge shape is similar to what was already observed in our previous study with PdIn-MOF.^33^ As soon as the **PdCo-MOF** sample is chemically treated (**PdCo-Q**), its spectrum (light blue) shifts slightly to lower energies; the white line decreases in intensity, and the edge shape changes, which indicates a partial reduction of Pd atoms.^33^ By pyrolyzing the sample after the chemical treatment (**PdCo-QT**, navy plot), the spectrum shifts completely to lower energies, the edge position resembles that of Pd foil, and the oscillations beyond the edge are similar to metallic palladium but slightly shifted and flattened, which indicates the formation of metallic Pd ensembles, possibly interacting with Co due to the differences with respect to the spectrum of Pd^0^. The observations from XANES are endorsed by the EXAFS spectra shown in Figure 5b. The initial sample (**PdCo-MOF**) shows two contributions related to Pd-L (L = C, N, O) and Pd–Cl between 1.0 and 2.2 Å (nonphase-corrected distances), respectively, which is in good agreement with the crystallographic information for this MOF. The chemically treated sample **PdCo-Q** maintains the first two Pd-L and Pd–Cl contributions from the remaining Pd in the MOF, but a Pd–Pd distance is now observed due to the partial reduction of Pd atoms. This Pd–Pd contribution is quite low in intensity (CN_Pd–Pd_ = 3.3 ± 0.9), which suggests the formation of a small fraction of Pd metal. Last, the EXAFS spectrum of **PdCo-QT** shows a Pd–Co contribution at ∼2.1 Å (CN_Pd–Co_ = 4.9 ± 0.2)^58^ and a small shoulder at ∼2.5 Å related to Pd–Pd contribution (CN_Pd–Pd_ = 4.9 ± 0.3) distances in agreement with the crystallographic structure identified by XRD analysis. The higher shells beyond 3 Å are not in phase with respect to those of metallic palladium, which reinforces the formation of the PdCo bimetallic ensembles.

The XANES spectra at the Pd K-edge of the different phases of the PdMn materials are shown in Figure 5e. The evolution from **PdMn-MOF** to **PdMn-QT** follows the same XANES features as in the case of PdCo catalysts, with Pd^2+^ in **PdMn-MOF**, partially reduced Pd atoms in **PdMn-Q** and metallic Pd in the **PdMn-QT** sample. However, the oscillations beyond the absorption edge of the **PdMn-QT** spectrum are in phase with those of the reference Pd^0^ with fcc structure, which hinders the suggestion of Pd and Mn alloying by XANES. The EXAFS spectra of the PdMn samples (Figure 5f) are also pretty similar to those of PdCo materials of Figure 5b, except that the **PdMn-QT** catalyst shows a broad contribution composed by Pd–Mn (CN_Pd–Mn_ = 6.8 ± 0.2) and Pd–Pd (CN_Pd–Pd_ = 4.1 ± 0.3) distances that are shifted to lower R-values in comparison with that of Pd foil, indicating that Pd atoms are alloyed with Mn.

The oxidation state and local environment of the Co atoms in PdCo materials were also assessed by XAS (Figure 5). Initially, Co atoms in **PdCo-MOF** are present as Co^2+^, which is seen by the typical position of the absorption edge around 7720 eV and the characteristic pre-edge peak at 7709 eV (XANES spectrum, Figure 5c), related to 1s → 3d transition of Co^2+^ compounds.^59^ Another characteristic of Co^2+^ is the high white-line intensity, typical of oxidized compounds. The spectrum of the sample after chemical treatment (**PdCo-Q**) remains the same as that in the as-synthesized **PdCo-MOF**, demonstrating that Co atoms are not as affected as Pd ones and stay within the MOF environment. Conversely, when the chemical-thermal treatment is performed, the XANES spectrum changes drastically, with the position of the absorption edge shifting to lower energies, indicating a reduction of Co^2+^ to Co^0^, and the spectrum shape does not resemble that of Co foil, suggesting the formation of bimetallic PdCo instead of segregated Co^0^ nanoparticles. EXAFS spectra in Figure 5d show that both **PdCo-MOF** and **PdCo-Q** present a main first shell related to Co–O bonds (1.6 Å, nonphase-corrected). After the chemical-thermal treatment, two contributions of Co–Co (2.0 Å) and Co–Pd (∼2.6 Å) distances appear related to the formation of bimetallic Co–Pd clusters. The coordination numbers for Co–Co and Co–Pd contributions are quite low (CN_Co–Co_ = 3.1 ± 0.1 and CN_Co–Pd_ = 4.5 ± 0.1), indicating tiny PdCo ensembles or highly disordered particles.

The XANES spectra of the PdMn-based materials are presented in Figure 5g. **PdMn-MOF** sample displays the position of the absorption edge (6547 eV) and a pre-edge peak at 6539 eV related to 1s → 3d transition, characteristic of Mn^2+^.^60^ Moreover, a highly intense white line typical of oxidized compounds is present in the spectrum of **PdMn-MOF**. The Mn atoms in **PdMn-Q** are partially reduced since a slight shift of the absorption edge to lower energies is perceived as well as the modification of the oscillations beyond the edge. Lastly, the spectrum of **PdMn-QT** shifted entirely to the same absorption edge position as that in Mn^0^ (6539 eV). However, the edge shape does not resemble it, reinforcing the previous observation in the Pd K-edge of Mn alloying with Pd. EXAFS data displayed in Figure 5h show that, for **PdMn-MOF**, a first shell related to Mn–O contribution of Mn in the MOF with CN_Mn–O_ = 4.0 ± 0.4 is present between 1.0 and 2.0 Å (nonphase-corrected). The partial reduction of Mn atoms upon chemical treatment can be seen in the EXAFS spectrum of the **PdMn-Q** sample, where a decrease in the intensity of the first shell and development of higher shells (between 2.0 and 4.0 Å, red spectrum) related to Mn^0^ can be perceived. Last but not least, the spectrum of the sample after chemical-thermal treatment displays a quite intense main contribution at 2.55 Å (nonphase-corrected) related to Mn atoms bound to Pd and Mn, with CN_Mn–Pd_ = 3.2 ± 0.5 and CN_Mn–Mn_ = 1.2 ± 0.4, respectively. The Tables containing all the information regarding EXAFS fits (coordination numbers, distances, Debye–Waller factors, etc.) can be found in the Supporting Information (Tables S5–S10).

**Figure 6:**
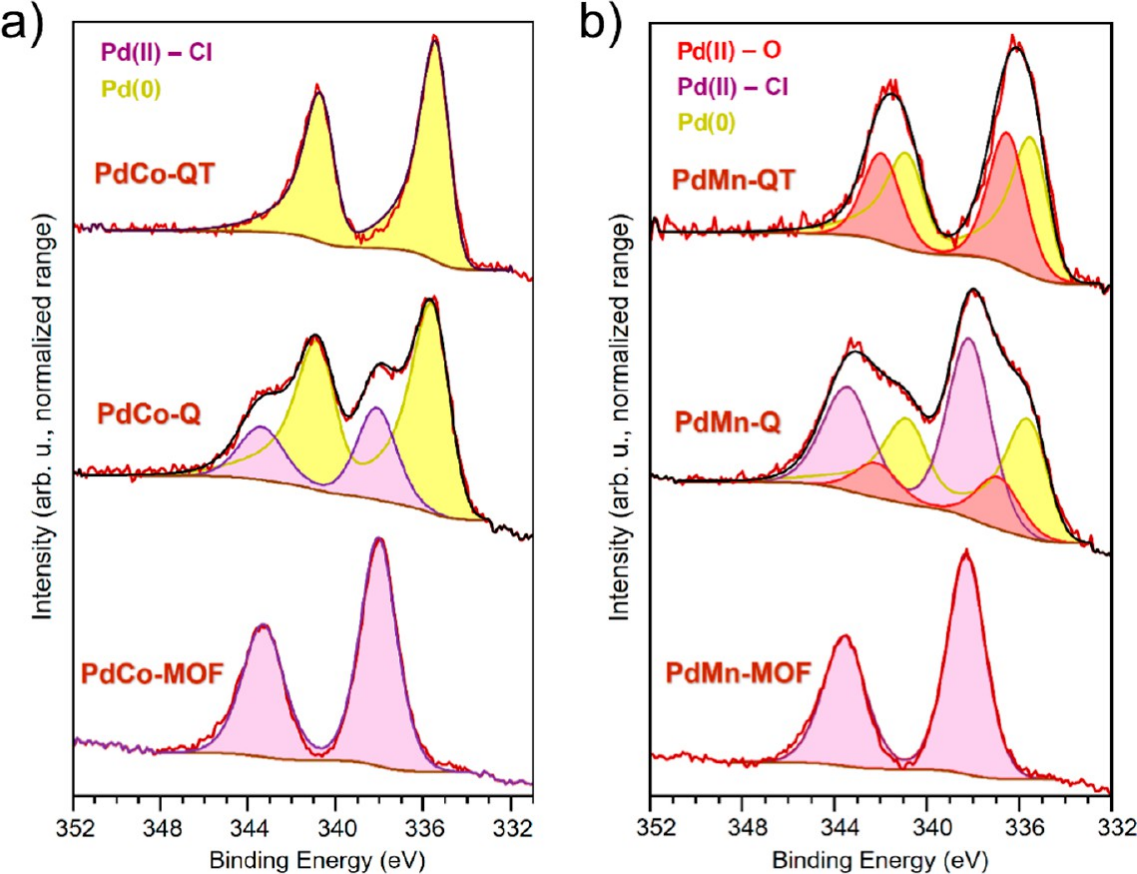
Pd 3d XP spectra of (a) PdCo-based samples, (b) PdMn-based samples.

Finally, the electronic states of the different elements composing the samples prepared in this work were also analyzed by XPS. Concerning the Pd 3d_5/2_ XP region in the PdCo materials (Figure 6a), the peak at higher B.E. is attributed to oxidized Pd, positively shifted (338.1 eV) in **PdCo-MOF** and **PdCo-Q**, suggesting the coordination of Pd to Cl^–^.^61^ This coordination to chlorine is lost after thermal treatment. Also, Pd^0^ 3d_5/2_ characteristic signals at lower B.E. are slightly upshifted (0.2 eV for **PdCo-QT** and 0.3 eV for **PdCo-Q**) with respect to a monometallic Pd-based sample consisting of the Pd metalloligand submitted to the chemical (Q) and thermal treatments (T) **Pd-H**_**4**_**L-QT** (Figures S2 and S3a). This shift may suggest an electronic interaction between Pd and Co.^62^ This reference sample (**Pd-H**_**4**_**L-QT**) contains Pd nanoparticles with shapes and sizes within the range of those attained in the PdCo and PdMn nanocomposites (Figure S2), and has also been used to model the line shapes of Pd^0^ 3d_5/2_ and Pd^0^ 3d_3/2_(LF(0.5, 6, 100, 400, 2) in CASAXPS). As for the Co 2p XP spectrum in the **PdCo-Q**, it remains identical to that of the **PdCo-MOF**, indicating a preservation of the original coordination environment that aligns with the XAS results. In both cases, the primary signal at the Co 2p_3/2_ region appears at 781.3 eV, while 797.1 eV is the position of Co 2p_1/2_ (Figure S16). That means a doublet separation of 15.8 eV. This splitting, along with a notable satellite feature at ca. 786.5 eV, is characteristic of high-spin Co^2+^.^63,64^ When subjected to thermal treatment, the Co into the **PdCo-QT** composite surface changes, with part of it present as Co^0^, resulting in a shoulder at ca. 778 eV. The oxidized fraction also shifts toward lower B.E. to **PdCo-MOF** and **PdCo-Q**, indicating a different nature. However, the low resolution of the spectrum makes any attempt to go deeper into the analysis of this oxidized Co highly adventurous. Co 2p XP spectra can be found in Supporting Information (Figure S16).

Regarding the PdMn materials (Figure 6b), the Pd 3d region is similar to that of the PdCo catalysts. In the case of **PdMn-MOF** and **PdMn-Q**, the spectra showed the presence of a shifted signal of surface-oxidized Pd, corresponding to Cl^–^ coordination.^61^ Again, at lower B.E., the Pd^0^ 3d_5/2_ signals are slightly upshifted compared to our **Pd-H**_**4**_**L-QT** monometallic reference (0.3 eV for **PdMn-QT** and 0.3 eV for **PdMn-Q**), also indicating some electronic interaction between Pd and Mn. On the other hand, the Mn 2p XP region (Figure S18) was not fitted due to low S/N (signal/noise) ratio and complex multiplet splitting. Therefore, the given information is limited (see Supporting Information).

**Figure 7:**
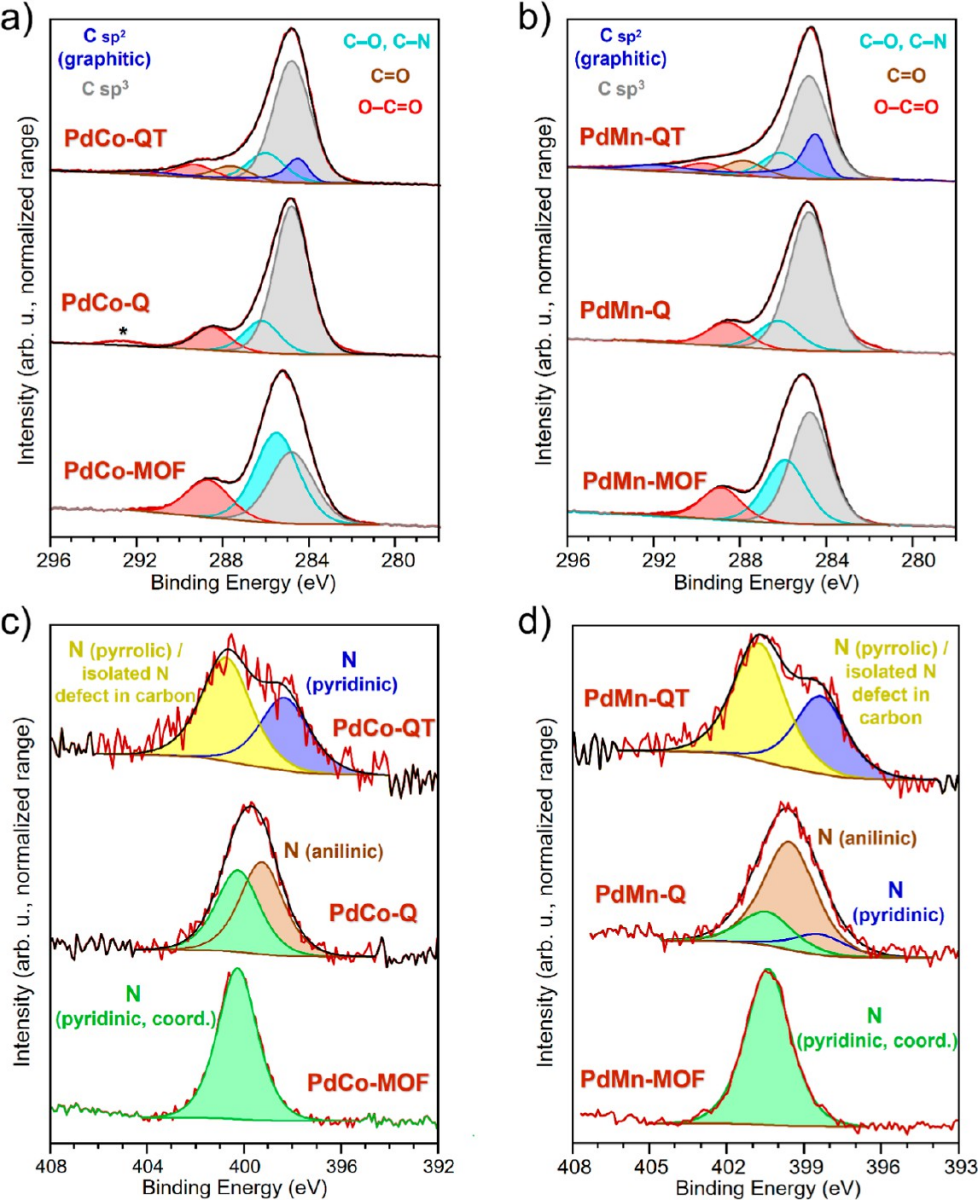
(a,b) C 1s XP spectra, (c,d) N 1s XP spectra of PdCo and PdMn-based samples, respectively. *: –CF_2_– contamination.

Finally, the electronic states of C and N in the PdCo and PdMn-derived materials can be discussed after the curve fitting of the corresponding regions. First, according to the C 1s XPS region, the previously mentioned graphitization of the carbon after the thermal treatment is observable for both **PdCo-QT** and **PdMn-QT** materials. In this sense, taking the line shape of a reference graphitic C 1s component (Figure S3b) as well as the fwhm ratio between C 1s (graphitic) and C 1s (sp^3^; C−C, C−H), it has been possible to introduce a significant graphitic contribution during the fitting of the C 1s XP region (Figure 7a,b). Satellite structures above 290 eV (π → π*) further justify the introduction of the component corresponding to this type of carbon.^65,66^ Then, for both **PdCo-QT** and **PdMn-QT** materials, the fitting of the N 1s region (Figure 7c,d) enables tracking the pyridine exit from the coordination sphere (from 400.3 to 400.4 in the PdM-MOFs to 398.3 eV in the **PdM-QT** materials).^67,68^ Additionally, the incorporation of a new type of nitrogen into the graphitic structure (400.7 eV) can be observed in the **PdM-QT** materials, likely due to isolated graphitic nitrogen defects or in-plane pyrrolic nitrogen.^69,70^ Finally, too many contributions in the oxygen XPS region may lead to risky and unpredictably inaccurate fitting for both material families (PdCo- and PdMn-derived materials). Figures S17 illustrate the complexity of this region, with estimated B.E. values for the expected contributions marked.

### Terminal Alkyne Catalytic Results (Phenylacetylene)

**Figure 8:**
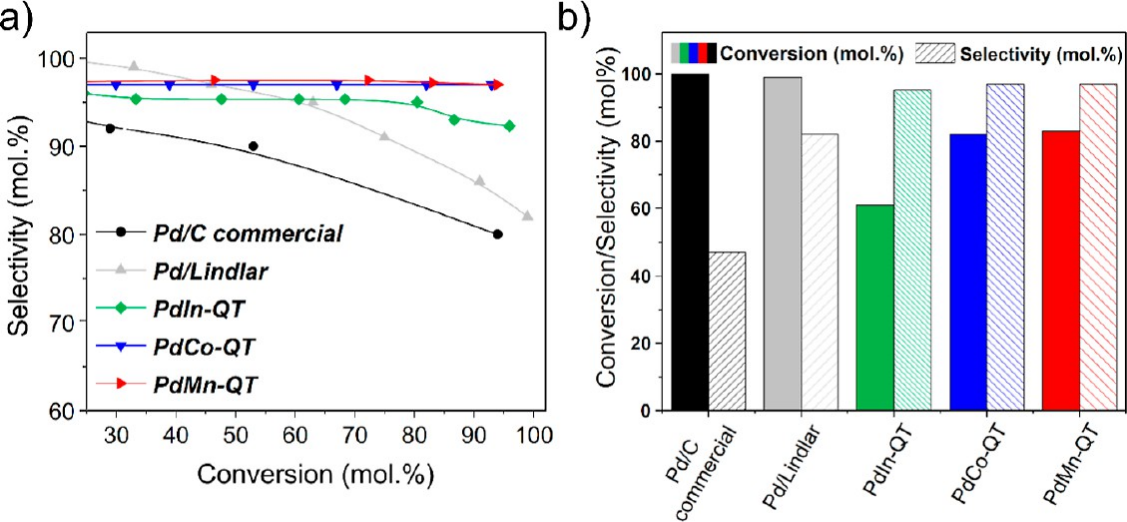
(a) Conversion vs selectivity plot of various PdM-based materials used in the selective hydrogenation of phenylacetylene, (b) activity and selectivity comparison of several Pd-based materials after 7 h of phenylacetylene hydrogenation reaction. Reaction Conditions: 5 mmol of phenylacetylene, substrate/Pd molar ratio: 323/1, 5 mL EtOH, r.t., 1 bar H_2_, 1000 rpm. Note: PdIn-QT reported in ref (33).

The catalytic activities of these first-row transition-metal-modified Pd-based materials were evaluated and compared for the selective hydrogenation of phenylacetylene. Remarkably, mild reaction conditions were used in this work: room temperature, ethanol as the solvent, and 1 bar of H_2_. Figure 8 exposes the corresponding results for the newly described **PdCo-QT** and **PdMn-QT** bimetallic materials. Notably, they have emerged as outstanding catalysts, selectively producing alkenes while achieving significant alkyne conversions. Their performance is benchmarked against a commercial Pd/C, and a Pd/Lindlar catalyst. As expected, the commercial Pd/C catalyst exhibited high activity but significant over-reduction and low selectivity toward alkenes, as shown in Figure 8. Also, the Lindlar catalyst is not particularly selective in the semihydrogenation of a terminal alkyne, with selectivity dropping below 90% when surpassing 90% conversion (Figure 8a). Indeed, when comparing **PdCo-QT** and **PdMn-QT** composites with commercial catalysts at conversion values nearing 100%, it becomes clear that the addition of these transition metals is beneficial for achieving high selectivity (>95 mol %) at elevated conversion levels (≥94 mol %). In that sense, Figure 8a also demonstrates that both Mn and Co demonstrate a slightly superior ability to modulate the selectivity of Pd at high conversions compared with our previously reported In-based system. Moreover, the catalytic activity at 7 h surpasses our previously reported catalyst (82% and 83% vs 61%), as depicted in Figure 8b.^33^ Tentatively, the catalytic behaviors of Pd–Mn, Pd–Co, and Pd–In (QT) systems in semihydrogenating alkynes can be explained by the electronic effects of the doping elements. XPS analyses revealed that Mn, Co, and In act as electron-withdrawing agents, inducing shifts in the Pd(0) 3d_5/2_ B.E. by +0.3, +0.2, and +0.4 eV,^33^ respectively (with respect to an analogous monometallic system). This electron withdrawal reduces the electron density around Pd, which might moderate its π-back–donation interaction with alkyne and alkene molecules, contributing to high selectivity by suppressing overhydrogenation. However, it seems that Mn and Co induce more subtle subtile shifts, also preserving high activity, whereas the stronger withdrawal effect of In also significantly reduces activity. Additionally, In, as a larger post-transition metal, may introduce geometric distortions in Pd–In NPs, further differentiating their catalytic behavior.^71,72^

Additionally, Table S12 compares the precursor materials involved in the current work to generate the QT material family. In entries 1 and 3 of Table S12, **PdCo-MOF** and **PdMn-MOF** exhibited the lowest activities among all tested bimetallic materials, with conversion values of 25% and 69% after 7 h, with selectivities of 91% and 87%, respectively. The outcome was expected because the MOF materials mainly consisted of oxidized palladium. Subsequently, materials derived from chemical treatment (**PdCo-Q**, and **PdMn-Q**) were evaluated. As shown in entries 2 and 4, **PdCo-Q** and **PdMn-Q** demonstrated similar activities, achieving 99% conversion, but low alkene selectivity (>80%) after a few hours of reaction. However, **PdCo-QT** and **PdMn-QT** materials displayed optimal catalytic performances (Table 2), meaning a selectivity of 97% (entries 3 and 4; Table 2) with conversion values of 95% and 96%, respectively, after 8 h. Several key points must be considered to understand the different behaviors of these bimetallic materials derived from **PdCo-MOF** and **PdMn-MOF**. The previous characterization studies (EM and XAS) revealed that the PdM-Q catalysts consist of monometallic Pd nanoparticles, with manganese or cobalt existing as isolated species associated with oxygen. In this context, the catalytic activity exhibited by both materials resembles that of the monometallic Pd/C reference to a greater extent. Thus, these materials demonstrate high activity but limited selectivity for the desired alkene. It is likely that the speciation resulting from the chemical treatment fails to establish the required bimetallic character necessary to regulate the Pd activity and prevent excessive reduction to alkane. Furthermore, Figure S19 shows the results from a kinetic study together with catalyst filtration tests for **PdCo-Q** and **PdMn-Q** materials. These tests, along with the corresponding ICP analysis results conducted at the end of the reaction (Table S13), indicate the metals are leaching out of the PdM-Q samples. Precisely, 2.3 ppm of Pd and 1.24 ppm of Co were detected in the final reaction mixture for the **PdCo-Q** catalysts. Similarly, for **PdMn-Q** materials, 0.66 ppm of Pd and 1.35 ppm of Mn were detected in the final reaction crude. In contrast, Table S16 shows the ICP results of the final crude in the case of the **PdMn-QT** and **PdCo-QT** catalysts. They exhibit a lower Pd leaching compared to the chemically treated materials. The corresponding characterization of the chemically treated materials before and after catalysis is available in the Supporting Information.

**Table 2:**
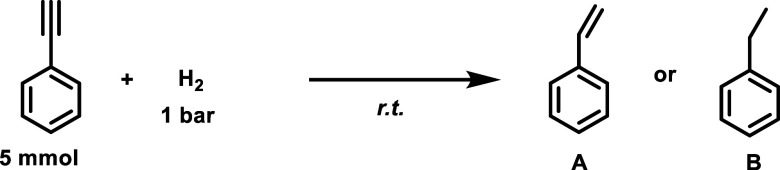
Comparison of Activity and Selectivity of Various Catalysts in Phenylacetylene Hydrogenation^a^

entry	catalyst	time (h)	conv (%)	selec. to A (%)	TON	TOF (h^–1^)	productivity (g_alkene_ g_cat_^–1^ h^–1^)
**1**	**Pd/C commercial**	5	94	84	303	60.6	5.0
**2**	**Pd/Lindlar**	7	99	82	319	45.6	1.4
**3**	**PdCo-QT**	8	95	97	152.7	19.1	10.1
**4**	**PdMn-QT**	8	96	97	133.3	16.7	13.5

a Reaction conditions: 5 mmol of phenylacetylene, substrate/Pd molar ratio: 323/1, 5 mL EtOH, r.t., 1 H_2_ bar. TON = mol of converted alkyne/mol of metal, TOF = TON·time^–1^.

**Figure 9:**
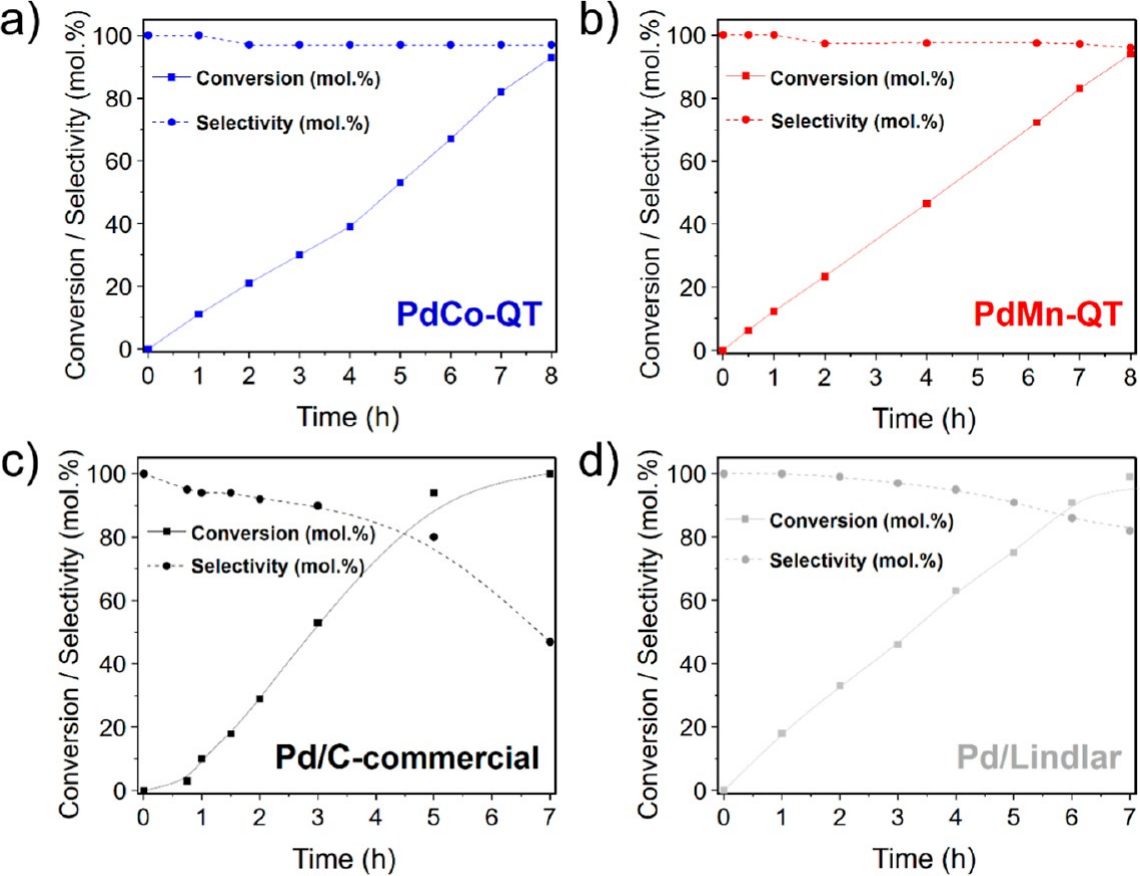
Kinetic curves of (a) **PdCo-QT**, (b) **PdMn-QT**, (c) **Pd/C commercial**, and (d) **Pd/Lindlar** catalysts. Reaction conditions: 5 mmol of phenylacetylene, substrate/Pd molar ratio: 323/1, 5 mL EtOH, 1000 rpm, r.t., 1 H_2_ bar.

Regarding **PdCo-QT** and **PdMn-QT** catalysts, the previous characterization showed that both comprise carbon-supported PdM bimetallic nanoparticles. Excitingly, both materials showed improved selectivity compared to commercial Pd/C and Pd/Lindlar (Table 2: entries 1 and 2). In fact, Figure 9 displays kinetic curves that show a significant drop in selectivity once conversions exceed ca. 80% and 90% for these two commercial catalysts, patterns not observed in the **PdCo-QT** and **PdMn-QT** materials. Also, the **PdCo-QT** catalyst was similarly active to **PdMn-QT** (Table 2: entries 3 and 4, and Figure 8b). However, the **PdCo-QT** catalyst shows higher performance regarding TON, and TOF, whereas the **PdMn-QT** materials present a higher productivity value (TON = mol of converted alkyne/mol of metal, TOF = TON·time^–1^, productivity = g_alkene_·g_cat_^–1^·h^–1^). To the best of our knowledge, the productivity values observed in the first catalytic cycle position our MOF-derived catalysts, **PdMn-QT** (13.5 g_alkene_·g_cat_^–1^·h^–1^) and **PdCo-QT** (10.1 g_alkene_ g_cat_^–1^·h^–1^), among the top four catalysts documented in the existing literature for the semihydrogenation of phenylacetylene at 1 H_2_ bar and temperatures below 50 °C (Table S15).

### Catalytic Stability of PdCo, PdMn Materials in the Phenylacetylene Semihydrogenation

**Figure 10:**
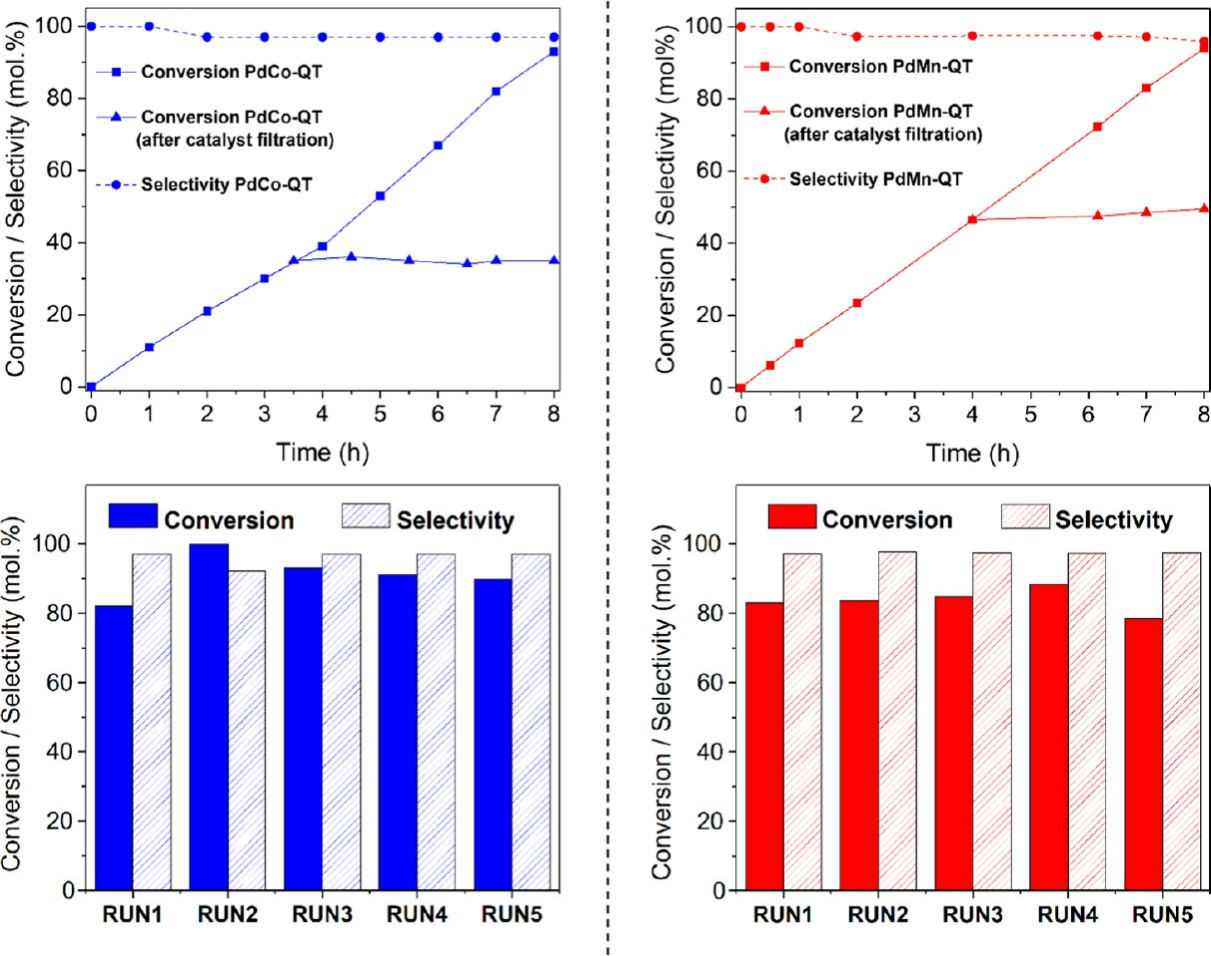
Catalyst filtration and stability cyclic test of **PdCo-QT** (left panel) and **PdMn-QT** (right panel) catalyst at 7 h of reaction time. Reaction conditions: 5 mmol of phenylacetylene, substrate/Pd molar ratio: 323/1, 5 mL EtOH, r.t., 1 bar H_2_, 1000 rpm.

In the following section, the stabilities of the PdCo and PdMn-derived materials are discussed. Figure 10 (top) depicts the kinetics of the **PdCo-QT** and **PdMn-QT** catalysts together with the catalyst filtration results. The corresponding ICP analyses conducted on the final reaction crude are provided in Table S16. As mentioned, the MOF-derived materials after chemical and thermal treatments are more stable than those after simple chemical modifications. Specifically, the **PdCo-QT** composite seems to exhibit better stability than the **PdMn-QT** catalyst. In that sense, the catalyst filtration tests reveal a conversion increase of 3% after the filtration of the **PdMn-QT** composite, while the conversion remains unchanged in the case of **PdMn-QT**. These experiments indicate that metals are leaching out of the material for the **PdMn-QT** sample to a more significant extent. In good agreement, the ICP results in Table S16 of the final crude showed a higher metal quantity in the case of **PdMn-QT** than for **PdCo-QT** (0.33 ppm of Pd and 2.86 ppm of Mn vs 0.13 ppm of Pd and 1.16 ppm of Co, respectively). It should be noted the low Pd leaching value in both cases compared to the commercial Pd catalyst (1.67 ppm). On the other hand, Figure 10 (bottom) shows the reusability tests of the chemically and thermally treated materials. These composites still display significant catalytic activity after 5 runs of phenylacetylene semihydrogenation reaction. Precisely, after five 7 h-long runs, the **PdCo-QT** can transform 5 mmol of alkyne at 90% conversion, keeping 97% selectivity, which translates into a productivity value of 12.6 g_alkene_ g_cat_^–1^·h^–1^. Even this fifth use stands out as one of the best-reported results for a Pd-based catalyst under similar reaction conditions in our study (Table S15). Furthermore, ICP results from the reaction crudes of each run are provided in Tables S17 and S18, which confirmed the minimal leaching of our **PdCo-QT** material. The slight deactivation observed in the case of the **PdMn-QT** during 5 runs can be attributed to minor leaching observed during the catalyst filtration tests in Figure 10 and Table S18.

Up to now, the discussion has led to considering both the **PdCo-QT** and **PdMn-QT** catalysts as well-balanced materials to maintain good activity with high selectivity. **PdCo-QT** and **PdMn-QT** catalysts possess very similar properties. The difference between both materials is mainly based on the nature of the nanoparticles. In that sense, the behavior of each catalyst will be governed by the nature of the interaction between the doping metal and the palladium. As demonstrated in the previous work, one of the critical roles of the doping metal is modulating the desorption rate of the intermediate.^33^ Nevertheless, at this point of the discussion, the excellent behavior of both catalysts does not permit us to distinguish any benefits in using Co or Mn as doping elements to modulate the Pd activity, but only in terms of catalyst stability. To understand the distinct behavior of these materials through the stability tests, the catalysts after runs 1 and 5 have been characterized by electron microscopy, XRD, Raman, and XPS.

First, Figures S23 and 11 show STEM-HAADF images of **PdCo-QT** and **PdMn-QT** catalysts after runs 1 and 5, respectively. Remarkably, the nanoparticle size distributions after run 5 confirmed a preservation of the nanoparticle size after the catalytic process. The fresh **PdCo-QT** material exhibited an average nanoparticle size of 16.1 ± 5.2 nm, and a nanoparticle size distribution of 14.5 ± 10.3 nm was estimated after the fifth run. Similarly, in the **PdMn-QT** catalyst, the fresh PdMn nanoparticles possessed an average size of 16.1 ± 4.3 nm before catalysis and 15.4 ± 9.0 nm after 5 catalytic runs.

**Figure 11:**
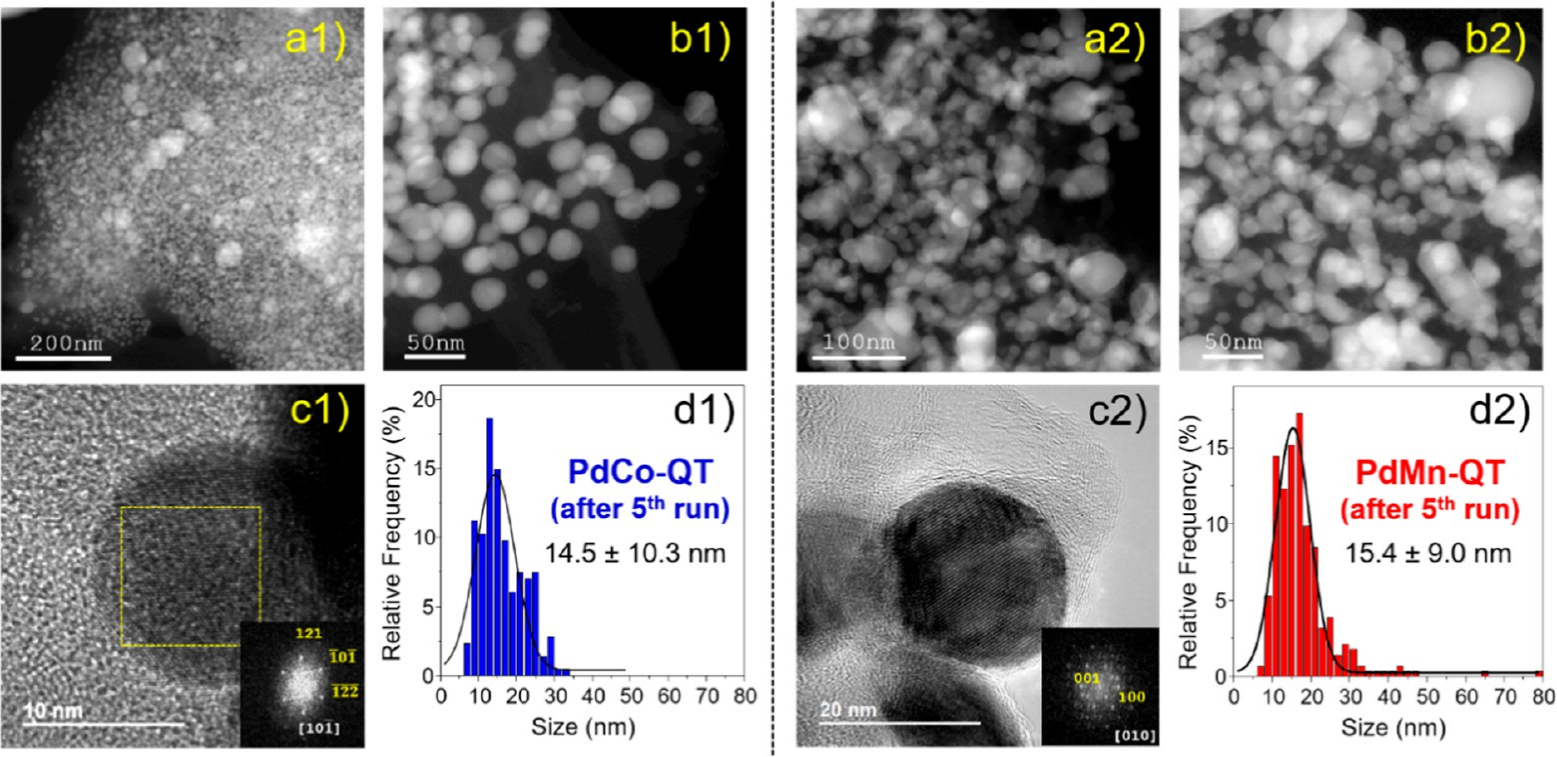
Electron microscopy characterization after 5 catalytic cycles of (1) **PdCo-QT** (left panel) and (2) **PdMn-QT** (right panel). (a,b) Representative STEM-HAADF images, (c) representative HR-TEM images and the measured interplanar distances. FFTs of the HRTEM images, depicting reflections characteristic of the phases PdCo and PdMn, are shown as insets; (d) nanoparticle size distributions.

The STEM–XEDS results of the **PdCo-QT** and **PdMn-QT** catalysts following 5 runs are depicted in Figures S24 and S25. The acquired maps demonstrate the retention of an intimate mixture between Pd and Co or Mn, thus pointing to the preservation of the bimetallic nature of the PdM nanoparticles. XRD diagrams of the postcatalytic materials were also acquired (Figure S26 and Table S19). First, the **PdMn-QT** materials demonstrated a high level of stability, as indicated by the consistency of their patterns before and after catalysis. This confirms that the material retains its original crystalline phases, which is consistent with the structure (interplanar distances and angles) measured in the FFTs of the image included in Figure 11c2 (Pd_1_Mn_1_ tetragonal NPs along the [010] zone axis). On the other hand, the XRD pattern of the **PdCo-QT** catalyst changes through the catalytic cycles, indicating restructuring under working conditions. As mentioned earlier, the XRD diagram of the fresh **PdCo-QT** catalyst showed the coexistence of two PdCo fcc bimetallic phases with different compositions, Pd_0.7_Co_0.3_ and Pd_0.9_Co_0.1_. This observation was based on the interpolation of the corresponding lattice parameters measured in the experimental XRD data, compared to the theoretical plot of the lattice constant parameter for the PdCo fcc crystal system versus atomic composition. After the first run, the material still exhibits the presence of two distinct fcc PdCo bimetallic systems, but with modified values of the lattice parameters of the two populations of NPs (see Table S19: 3.79 and 3.76 Å). The same interpolation procedure indicates that after the first run, the system consists of Pd_0.60_Co_0.40_ and Pd_0.55_Co_0.45_ fcc nanoparticles. This suggests the occurrence of a Co/Pd remixing process that brings the compositions of the two families of NPs to a closer molar ratio. Finally, after the fifth run, the material is based on a single Pd_0.45_Co_0.55_ fcc crystalline phase with 3.74 Å as the lattice constant value and 13.1 nm as the average crystal size. Additional HR-HAADF-STEM studies on this phase, along with the compositional analysis, point to this phase being an intermetallic PdCo (Figure S14). These composition changes must be linked to sintering processes, very likely induced by Ostwald ripening.^73^ To account for the global enrichment of Co in the NPs, additional cobalt remaining in the carbon should also be incorporated into the NPs under the reductive conditions used during the catalysis process. Additionally, the characteristic peak at 18° corresponding to the (111) CoCo_2_O_4_ (fcc) was still detected, which indicates the persistence of this phase under the working conditions.

Additionally, Figure S27 and Table S20 compare the Raman spectra of **PdCo-QT** and **PdMn-QT** after runs 1 and 5 with those of the fresh materials. The spectra exhibit remarkable similarity before and after catalysis, indicating the stability of the carbon support. The characteristic graphitic carbon G band appears around 1589 cm^–1^, and the *I*_D_/*I*_G_ ratio suggests a low order of carbon without drastic changes throughout catalysis. Notably, after catalysis, the peaks at 667 and 642 cm^–1^, corresponding to CoOx and MnOx species, respectively, disappeared. This strengthens the previous suggestion that the reaction mixture reduces these phases during catalysis.

Finally, XPS analysis was also performed on the final materials after 5 reaction cycles (Figures S28–S31). Concerning the **PdCo-QT** composite, the only change appreciated in the Pd 3d region is the incorporation of a component at higher B.E. (336.9 eV), which is characteristic of Pd^2+^, suggesting a small surface oxidation, likely due to ambient exposure (Figure S28b). Also, the C 1s region (Figure S28a) showed an increasing contribution after catalysis (286.5 eV), indicating the presence of C–O from the reaction solvent (e.g., ethanol). Apart from this component, the C 1s region spectra are very similar before and after catalysis. Lastly, the N 1s region (Figure S30) was not fitted due to the poor resolution of the spectra. Nevertheless, the similar profiles before and after catalysis show a dominant peak centered around 400.7 eV in both cases, indicating good preservation of the N doping the graphitic carbon as an isolated graphitic nitrogen defect or an in-plane pyrrolic nitrogen.^69,70^ The C 1s and N 1s XP spectra confirm Raman’s discussion. Unfortunately, the bad quality of the Co 2p region does not allow us to discuss the Co electronic state after catalysis. Oppositely, the Pd 3d region of the **PdMn-QT** composite after 5 catalytic cycles showed an increase in the characteristic Pd^0^ 3d_5/2_ signal at lower B.E. (335.2 eV), suggesting a more reduced character of palladium (Figure S29b). The Mn 2p region of the material after five uses was not fitted due to the complexity associated with the presence of multiplet splitting and the insufficient quality of the spectrum. Nonetheless, the Mn 2p XP profiles appear to be unchanged after the catalytic process (Figure S31a). Then, the speciation in both C 1s and N 1s regions is nearly identical before and after the reaction, demonstrating good preservation of the N-doped graphitic carbon.

Considering the higher stability of the **PdCo-QT** system, we focused on this material to experimentally demonstrate its selectivity toward alkenes. In that sense, an experiment with a mixture of alkynes and alkenes in a 1:9 ratio was conducted (Figure S22). Despite the higher alkene concentration, the **PdCo-QT** catalyst completely converted the alkyne after just 6 h, while the alkene conversion was still negligible. In this respect, the behavior is very similar to that previously reported for PdIn-QT.^33^ As the indium-modified composites, the new **PdCo-QT** material probably has a weaker styrene adsorption capacity as well as a stronger interaction with phenylacetylene. These facts should certainly be the driving force allowing the material to maintain the selectivity toward alkene production. DRIFT analyses could be conducted to shed some light on the selectivity of the **PdCo-QT** catalyst. Unfortunately, the dark nature of the sample does not allow the acquisition of spectra with adequate signal-to-noise ratio.

### Internal Alkyne Catalytic Results (4-Octyne)

**Figure 12:**
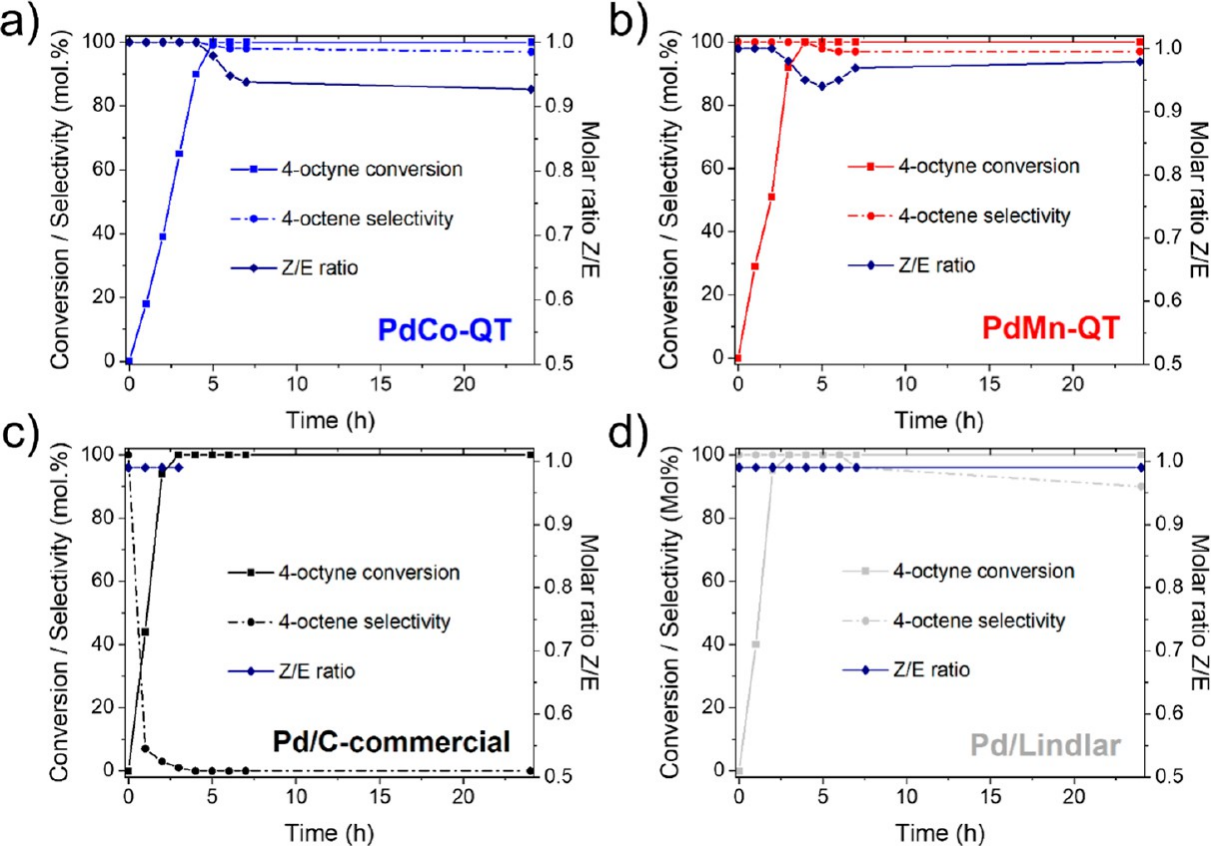
(a–d) Catalytic batch results of 4-octyne selective hydrogenation with **PdCo-QT**, **PdMn-QT**, **Pd/C**, and **Pd-Lindlar** catalysts, respectively. Reaction conditions: 5 mmol of 4-octyne, substrate/Pd molar ratio: 323/1, 5 mL EtOH, 1000 rpm, r.t., 1 bar H_2_.

In order to broaden the catalytic scope of the developed composites **PdCo-QT** and **PdMn-QT**, we attempted hydrogenation of an internal alkyne (4-octyne). Interestingly, Figure 12 demonstrates how the two materials can achieve selectivity values above 95% after quantitative conversions were reached (Table 3). On the contrary, the Pd/C catalyst displays a dramatic drop in selectivity above 10% conversion. Even the Pd/Lindlar catalyst, renowned for its efficacy in hydrogenating internal alkynes, experiences a reduction in selectivity when the resulting alkene remains in contact with the catalyst after quantitative conversion. In contrast, our materials demonstrate sustained stability under analogous conditions, avoiding the loss of selectivity. This fact clearly highlights the specificity of the herein-developed catalysts to react with alkynes vs alkenes.

**Table 3:** Comparison of Activity and Selectivity of Different Catalysts in 4-Octyne Hydrogenation^c^

catalyst	aalkyne conversion (mol %)	aalkene selectivity (mol %)	amolar ratio (*Z*/*E*)	balkyne conversion (mol %)	balkene selectivity (mol %)	bmolar ratio (*Z*/*E*)
**PdCo-QT**	99	99	0.97	99	97	0.93
**PdMn-QT**	99	98	0.94	99	97	0.98
**Pd/C commercial**	99	0		99	0	
**Pd/Lindlar**	99	99	0.99	99	90	0.99

a 5 h.

b 24 h.

c Reaction conditions: 5 mmol of 4-octyne, substrate/Pd mol ratio: 323/1, 5 mL EtOH, r.t., 1 bar H_2_, 1000 rpm.

### Process Intensification Using **PdCo-QT** and **PdMn-QT** Materials

Up to now, we have presented the successful extension of our methodology to generate N-doped carbon-supported bimetallic materials based on the use of a first–row transition metal, instead of In (our previous work),^33^ to modify the Pd. The excellent behavior of these new composites has been demonstrated in the liquid phase selective semihydrogenation of phenylacetylene and 4-octyne in mild conditions. Consequently, we decided to intensify the selective production of styrene and 4-octene, testing the activity of these materials in a continuous gas-phase reactor (see Supporting Information: gas-phase general procedure).

**Figure 13:**
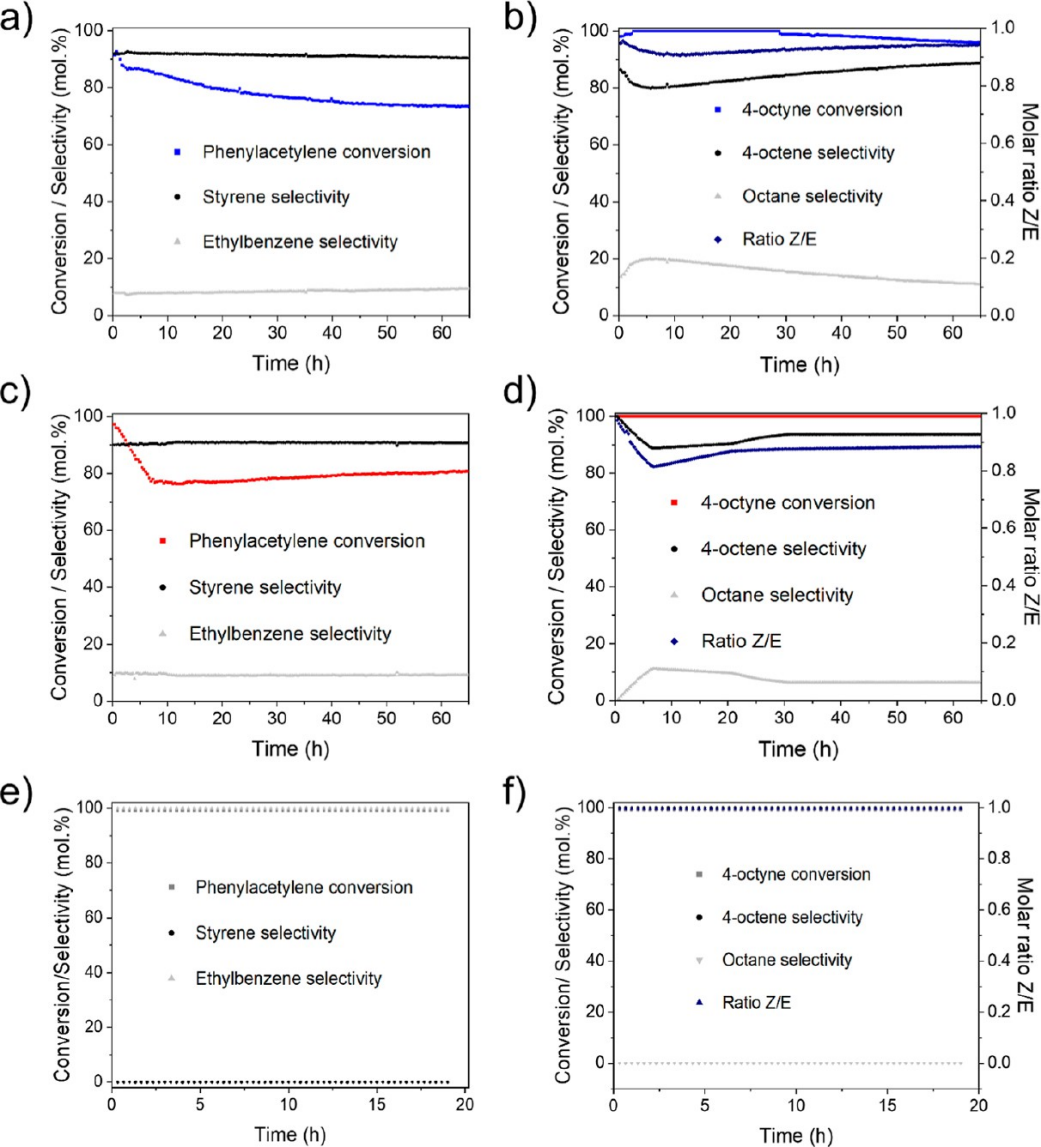
Catalytic flow results of (a,c,e) phenylacetylene and (b,d,f) 4-octyne selective hydrogenation with **PdCo-QT**, **PdMn-QT**, and **Pd-Lindlar** catalysts, respectively. Reaction Conditions: 150 °C, see Table 4.

The conditions, especially the nitrogen flow, were optimized for high conversion (≥75%) while maintaining a high selectivity (≥90 mol %) for each catalyst. The catalytic bed was prepared with 5 mg of **PdCo-QT** and **PdMn-QT**. The catalyst was physically mixed with SiC and normalized to 1.2 mL of volume. In the case of the Pd/Lindlar catalyst, the catalyst loading was adjusted to normalize the palladium quantity to get a meaningful comparison with our materials. It was also determined that the temperature should be maintained at 150 °C to prevent condensation of the resulting product and to ensure the successful carbon balance closure. Finally, after 1 h of catalyst activation under H_2_ at 150 °C, time-on-stream experiments were carried out with both catalysts up to 65 h. After this time, the **PdMn-QT** catalyst presents a conversion of 81% with 91% selectivity to the desired alkene. Therefore, in the gas phase reaction, the manganese-based catalyst showed an interesting behavior exhibiting a GHSV of 150,000 mL·h^–1^·g_cat_^–1^, high selectivity, and high stability. Even more excitingly, the **PdCo-QT** catalyst exhibits a very similar behavior, but operates with a GHSV of 336,000 mL·h^–1^·g cat^–1^, which is more than twice the **PdMn-QT** value. This implies much higher productivity in terms of alkyne produced per unit time and unit mass of catalyst (**PdCo-QT** productivity = 13.2 h^–1^, **PdMn-QT** productivity = 10.3 h^–1^). All of these results are summarized in Table 4. Figure 13 also demonstrates the versatility of these catalysts in comparison to that of the Lindlar catalyst. Our catalyst successfully achieves the hydrogenation of both terminal (i.e., phenylacetylene) and internal alkynes (i.e., 4-octyne), while the Lindlar catalyst is limited to internal alkynes.

**Table 4:**
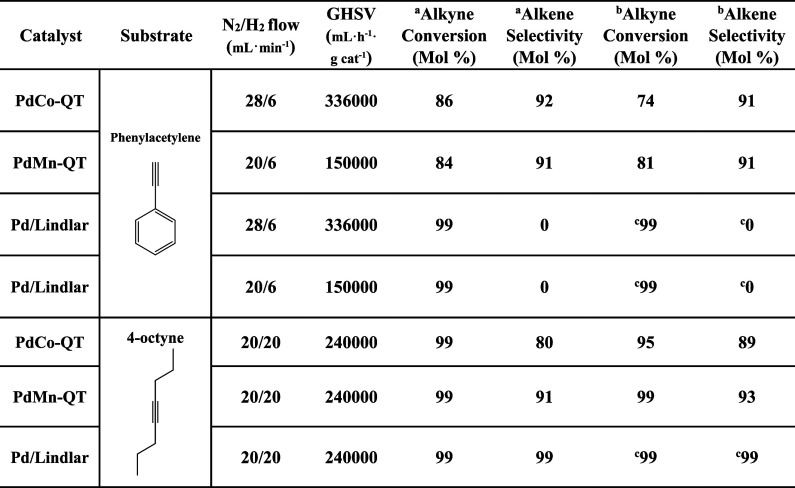
Comparison of Activity and Selectivity of **PdCo-QT**, **PdMn-QT**, and Pd-Lindlar Catalysts in Continuous Gas Phase Selective Hydrogenation^d^

a 5 h.

b 65 h.

c 19 h. GHSV = mL_alkyne+N2_·time^–1^·g_cat_^–1^.

d Reaction conditions: 150 °C.

## Conclusions

Using combined chemical and thermal treatments, this work successfully developed PdCo- and PdMn-based bimetallic nanocomposites from MOF precursors. The objective was to improve specificity and selectivity of traditional Pd-based catalysts for alkyne semihydrogenation reactions and explore a cost-effective alternative to the previously reported PdIn-based catalyst^33^ by replacing indium with a first-row transition metal. These materials, as characterized by advanced spectroscopic (Raman, XAS, and XPS) and electron microscopy (HR-TEM, HR-STEM-HAADF, STEM–XEDS, and STEM EELS) techniques, formed well-defined Pd-based bimetallic nanoparticles supported on a N-doped carbon substrate. The catalytic evaluation for the semihydrogenation of phenylacetylene revealed that both **PdCo-QT** and **PdMn-QT** materials exhibit high selectivity and activity under mild reaction conditions (1 H_2_ bar and r.t.), clearly surpassing the productivity values previously reported for our PdIn-QT catalyst (10.1 and 13.5 g_alkyne_·g_cat_^–1^·h^–1^).^33^**PdCo-QT** showed superior stability and performance compared to **PdMn-QT**, particularly in terms of lower metal leaching as well as higher turnover frequencies. Moreover, the activity of the two systems could be extended to the hydrogenation of an internal alkyne (i.e., 4-octyne), showing slightly lower activity than the Pd/Lindlar catalyst but without experiencing a reduction in selectivity when the resulting alkene remains in contact with the catalyst after quantitative conversion. Overall, the versatility of these systems, capable of hydrogenating both internal and terminal alkynes, is superior with respect to the Pd/Lindlar catalyst. In continuous gas-phase reactions, **PdCo-QT** exhibited a promising performance, with high GHSV and stable activity over extended periods (up to 70 h).

Further improvements might be achieved by optimizing the nanoparticle sizes of the bimetallic systems. Our group is conducting preliminary investigations that suggest that modifications on the chemical treatment, e.g., the nature of the nitro group, can provide better control over particle size, porosity, and nitrogen content. These strategies represent a promising avenue for further enhancing the activity and selectivity of these catalysts.

In summary, this study highlights the potential of MOF-derived PdM bimetallic systems as robust and efficient catalysts for selective hydrogenation reactions. It should also continue strengthening the community’s confidence in MOF-driven synthetic procedures to achieve sophisticated catalyst designs with extraordinary behaviors.

## Supporting Information Available

Supporting information includes additional descriptions of materials and procedures used; characterization studies (TEM and XPS) on the PdM-MOF and PdM-Q materials; additional XAS data; complementary tests to evaluate the catalytic activity and the catalytic stability of the materials; and literature reference data (PDF)
