# Supplementary material for: MOF-Derived PdCo and PdMn Systems as Versatile Catalysts in Alkyne Semihydrogenation
Source: ACS Catal. 2025 Apr 18;15(9):7263–82. doi: 10.1021/acscatal.4c07149 (PMC12053837; doi:10.1021/acscatal.4c07149)
Supplement: Supplementary file 1 — cs4c07149_si_001.pdf [file cs4c07149_si_001.pdf]

MOF-derived PdCo and PdMn systems as versatile catalysts in alkyne semi-hydrogenation

[1]Jordan Santiago Martinez, [2]Luigi Carpisassi, [1]Gonzalo Egea, [1#*]Jaime Mazarío, [3]Christian Wittee Lopes, [4,5]Carmen Mora-Moreno, [4,5]Susana Trasobares, [2]Luigi Vaccaro, [4,5]Jose Juan Calvino, [6]Giovanni Agostini, and [1*]Pascual Oña-Burgos

[1] *Instituto de Tecnología Química, Universitat Politècnica de València-Consejo Superior de Investigaciones Científicas (UPV-CSIC), Avda. de los Naranjos s/n, 46022 Valencia, Spain.*

[2] *Laboratory of Green S.O.C – Dipartimento di Chimica biologia e Biotecnologie, Università degli Studi di Perugia, Via Elce di Sotto 8, Perugia 06123, Italy.*

[3] *Department of Chemistry, Federal University of Paraná (UFPR), Curitiba, 81531-990, Brazil.*

[4] *División de Microscopía Electrónica de los Servicios Centralizados de Investigación Científica y Tecnológica de la Universidad de Cádiz (DME-UCA), Facultad de Ciencias, Universidad de Cádiz, Campus Río San Pedro S/N, Puerto Real 11510, Cádiz, Spain.*

[5] *Departamento de Ciencia de los Materiales e Ingeniería Metalúrgica y Química Inorgánica, Facultad de Ciencias, Universidad de Cádiz, Campus Río San Pedro S/N, Puerto Real 11510, Cádiz, Spain.*

[6] *ALBA Synchrotron Light Facility, Carrer de la Llum 2-26, Cerdanyola del Valles, Barcelona 08290, Spain.*

# Current address: LPCNO (Laboratoire de Physique et Chimie des Nano-Objets), Université de Toulouse, CNRS, INSA, UPS, 31077 Toulouse, France.

*corresponding authors: pasoabur@itq.upv.es, mazario@insa-toulouse.fr

# - Supporting Information -

Index

I- Catalytic tests and stability **3**

II- Additional characterization and catalytic data **4**

Pd-H_4L-QT monometallic reference 4

XPS reference spectra 5

Additional characterization of the catalyst precursors PdCo-MOF and PdMn-MOF 6

Additional PdM-Q and QT characterization 8

PdM-MOF and PdM-Q activity/stability 19

PdM-QT activity/stability 23

III- References **31**

I- Catalytic tests and stability

- **Equations to evaluate catalytic activity and selectivity**

$$\text{(A) Conversion (\%)} = \frac{\text{initial mol of alkyne} - \text{final mol of alkyne}}{\text{mol initial of alkyne}} \times 100$$

$$\text{(B) Selectivity (\%)} = \frac{\text{mol of alkene}}{\text{mol of alkyne converted}} \times 100$$

$$\text{(C) TON (Turnover Number)} = \frac{\text{mol of alkyne converted}}{\text{mol of metal in the catalyst}}$$

$$\text{(D) TOF (Turnover Frequency, } h^{-1}) = \frac{\text{TON}}{\text{Time}}$$

$$\text{(E) Productivity } (h^{-1}) = \frac{\frac{\text{final mass of alkene}}{\text{mass of catalyst}}}{\text{Time}}$$

$$\text{(F) GHSV} = \frac{\text{flow rate of alkyne } \left(\frac{mL}{h}\right)}{g_{cat}}$$

- **Continuous gas-phase reactor setup**

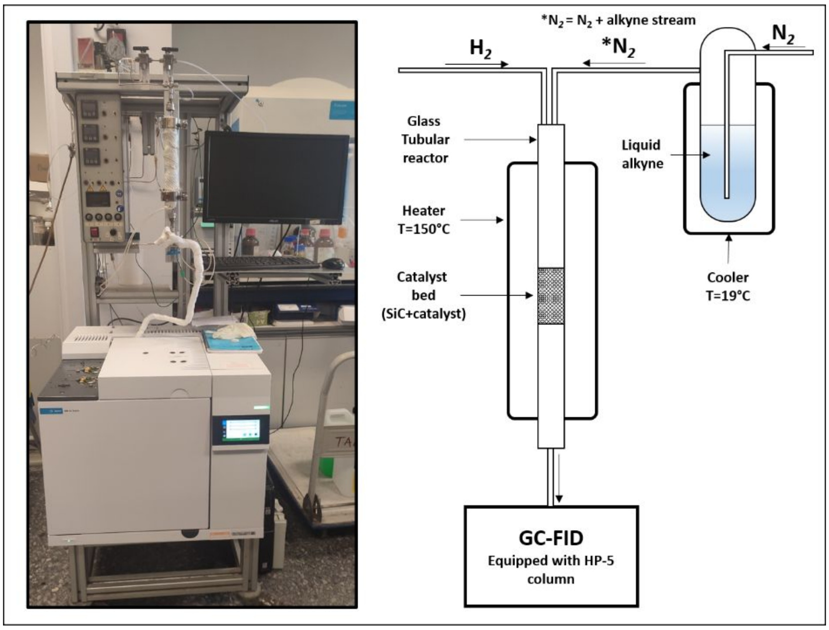

Figure S1. Schematic of the system used for the gas-phase catalytic hydrogenation reactions.

II- Additional characterization and catalytic data

- **XRD formula**

equations used to evaluate the crystallite size (1) and the lattice parameters (2-3):

$$\text{(1)}\quad \boldsymbol{Dp = \frac{K \times \lambda}{\beta \times Cos\,\theta}}$$

Dp= Average cystalite size, **K**=shape factor (0.94), **β**= FWHM (Full Width at Half Maximum), ***θ***= Bragg angle, **λ**= X-ray wavelength.

$$\text{(2) Cubic system (a=b=c):}\quad \boldsymbol{d_{hkl} = \frac{a}{\sqrt(h^2+k^2+l^2)}}$$

$$ie\text{:}\ d_{111} = \frac{a}{\sqrt{3}}$$

$$\text{(3) Tetragonal system (a=b}\neq\text{c):}\quad \boldsymbol{\frac{1}{(d_{hkl})^2} = \frac{h^2+k^2}{a^2} + \frac{l^2}{c^2}}$$

$$ie\text{:}\ \frac{1}{(d_{110})^2} = \frac{h^2+k^2}{a^2},\ a = b = \sqrt(\frac{2}{d_{110}})$$

$$ie\text{:}\ \frac{1}{(d_{001})^2} = \frac{l^2}{c^2}\ ,\ c = 2 \times d_{002}$$

- **Pd-H_4L-QT monometallic reference**

We applied our methodologies (chemical-thermal treatments) to transform the common building unit of our MOFs, specifically the Pd-H_4L metal complex. The main goal of creating this material was to obtain a reliable Pd on carbon reference to gain a deeper understanding of the influence of the doping metal on the previously presented materials. In this sense, Figure S2 presents the characterization by HAADF-STEM and XRD of the resulting materials after chemical and thermal transformations of the Pd monometallic metalloligand. HAADF-STEM results show the correct transformations of our metal complex after chemical and thermal treatments with the presence of nanoparticles with an average size of 14.4 ± 6.2 nm. Then, the XRD diagram of **Pd-H_4L-QT** shows the diffraction maxima expected for fcc Pd with an experimental lattice parameter of 3.89Å (peaks at 40.2°, 46.7°, 68.3°, 82.2° and 86.7° corresponding to (111), (200), (220), (311) and (222)), in good agreement with the JCPDS reference (98-006-0960). In conclusion, the correct transformation of the **Pd-H_4L** metal complex to carbon-supported Pd NPs has been demonstrated, and the material will be used as a reference for XPS analyses.

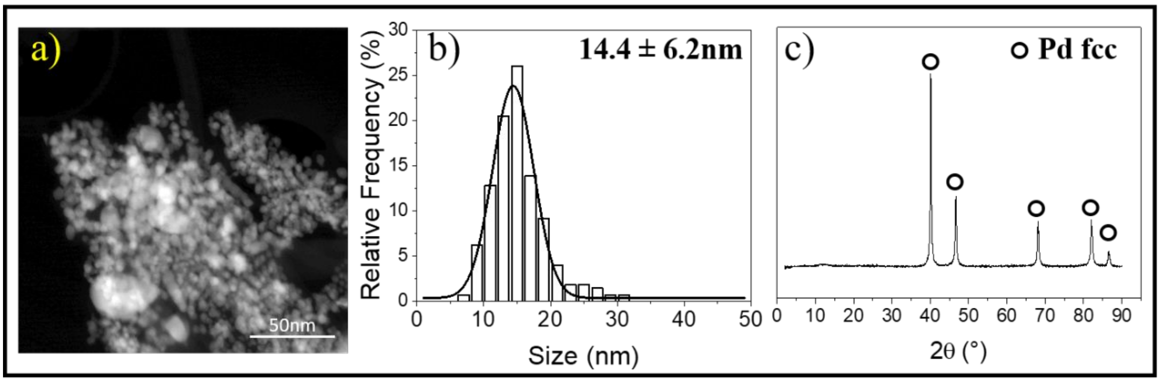

Figure S2. a) HAADF STEM image, b) the corresponding nanoparticle size distributions, and c) XRD spectra of the **Pd-H_4L-QT** sample.

- **XPS reference spectra**

Several spectra were taken with the sole purpose of establishing dependable references for curve fitting. First, in Figure S3a, the LF lineshapes (0.5,6,100,400,2) for the Pd^0 doublet components in the Pd*3d* XP region were estimated after curve fitting of this signal in a reference sample consisting of the Pd-H_4L metalloligand submitted to chemical and thermal treatments (i.e., **Pd-H_4L-QT**). As previously demonstrated in Figure S2 (see previous section: ***Pd-H4L-QT*** *monometallic reference*), this sample contains Pd NPs with shapes and sizes within the range of those attained in the **PdCo-QT** and **PdMn-QT** nanocomposites. Additionally, a hydrogen pretreatment was conducted at 250 ºC in the XPS antechamber to ensure a complete reduction of the palladium surface in this sample. The fitting included two doublet components for Pd^0, and two other component peaks corresponding to satellite plasmon peaks of Pd*3d*, in agreement with those reported before by Militello *et al.*[1]

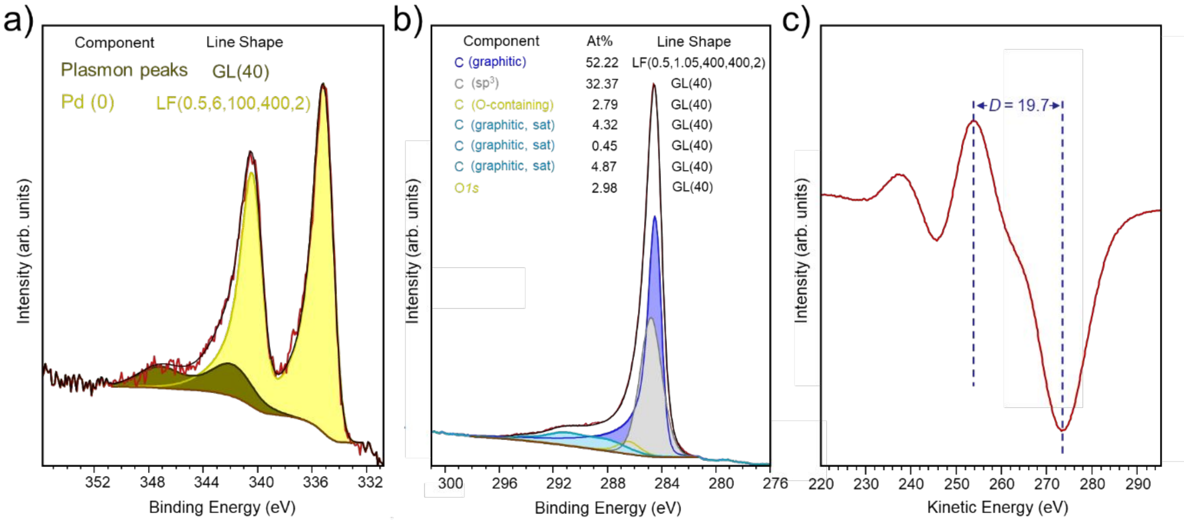

Figure S3. a) Pd*3d*, b) C*1s* XPS regions of **Pd-H_4L-QT** (reduced) and a **graphitic carbon** reference, respectively, c) first derivative C (KVV) Auger of the graphitic carbon reference.

Additionally, in Figure S3b, a curve fitting is presented for a reference sample (graphitic carbon, *Sigma ref 282863*). The peak fitting model for this reference sample was built upon using as a starting point the atomic ratio of C (sp^3): C (sp^2) estimated from the D parameter (being aware of the difference in information depth between the C*1s* XP and the Auger electron), and keeping a difference between the concentration of C-O-containing peaks in the C*1s* region and the total O*1s* region below 1 at.%, as recommended in the work of D.J. Morgan.[2] Following this fitting, the LF lineshape (0.5,1.05,400,400,2) for the graphitic component in the C*1s*, the FWHM ratio between C*1s* (graphitic) and C*1s* (sp^3) components, and the intensity ratio between the π–π* shake-up structure and the main peak were taken and used in the fitting of the C*1s* region in the problem samples.

- **Additional characterization of the catalyst precursors PdCo-MOF and PdMn-MOF**

SEM analysis has been carried out to study the crystal morphology of the new **PdCo-MOF** and **PdMn-MOF** samples (Figure S5), finding well-defined, bladed tubular/acicular crystals of 5-60 µm in length. The chemical information provided by SEM-EDS confirmed the presence of palladium and cobalt or manganese atoms in the observed crystals. According to the bulk ICP/AE results in Table S1, **PdCo-MOF** and **PdMn-MOF** showed a Pd/M (M: Co, Mn) molar ratio of 1/1.4 and 1/1.7 respectively, which is fairly close to the previously reported PdIn-MOF constituted by the analogous subunit $[M^{1}_{3}(\mu_3\text{-}O)(-COO)_6]$.[3,4] Furthermore, the XRD analysis depicted in Figure S4 exhibits a diffraction pattern that matches the expected outcome. **PdCo-MOF** and **PdMn-MOF** showed a pattern similar to that of above-mentioned PdIn-MOF, with a pattern shift due to indium substitution by Co or Mn of 1.20° and 1.25°, respectively. The difference in the relative intensity between peaks compared to the PdIn-MOF can be explained considering the difference in crystal shape observed by previous SEM results (PdIn-MOF: edge truncated cube vs PdCo/PdMn-MOF: bladed tubular/acicular).

Table S1. ICP and EA results for **PdIn-MOF, PdCo-MOF** and **PdMn-MOF** samples.

Material	Pd wt%[a]	M	M wt%[a]	Pd/M molar ratio	N wt%[b]	C wt%[b]	H wt%[b]
PdIn MOF[4]	**9.3**	**In**	**18.5**	**1/1.8**	**5.1**	**24.0**	**2.6**
PdCo MOF	**11.7**	**Co**	**8.9**	**1/1.4**	**7.1**	**30.9**	**3.6**
PdMn MOF	**10.1**	**Mn**	**9.2**	**1/1.7**	**7.5**	**32.3**	**3.6**

[a] Calculated by ICP, [b] Calculated by EA.

The thermal stability of the MOFs was evaluated by TGA under airflow (Figure S5b). Upon heating, these MOFs exhibit multiple weight-loss stages. Below 100 °C, the weight loss is attributed to the evaporation of adsorbed solvent molecules. Additional weight losses occur at 200 °C and 300 °C, likely due to the elimination of coordinated molecules. At temperatures above 400 °C, the weight loss corresponds to the structural breakdown of the MOF as the metallo-ligand decomposes, culminating at 600 °C (Figure S5b). At 800 °C, 29 wt% of residues remained for both MOFs.

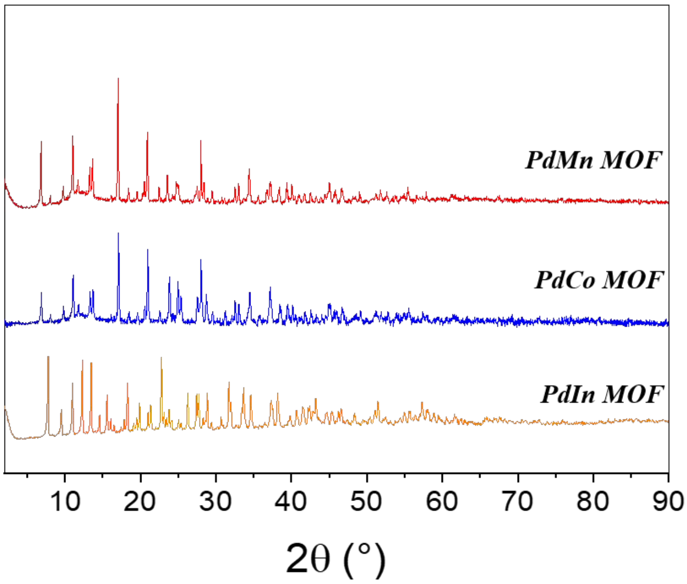

Figure S4. XRD patterns of PdM-MOFs materials (M= In,[4] Co or Mn).

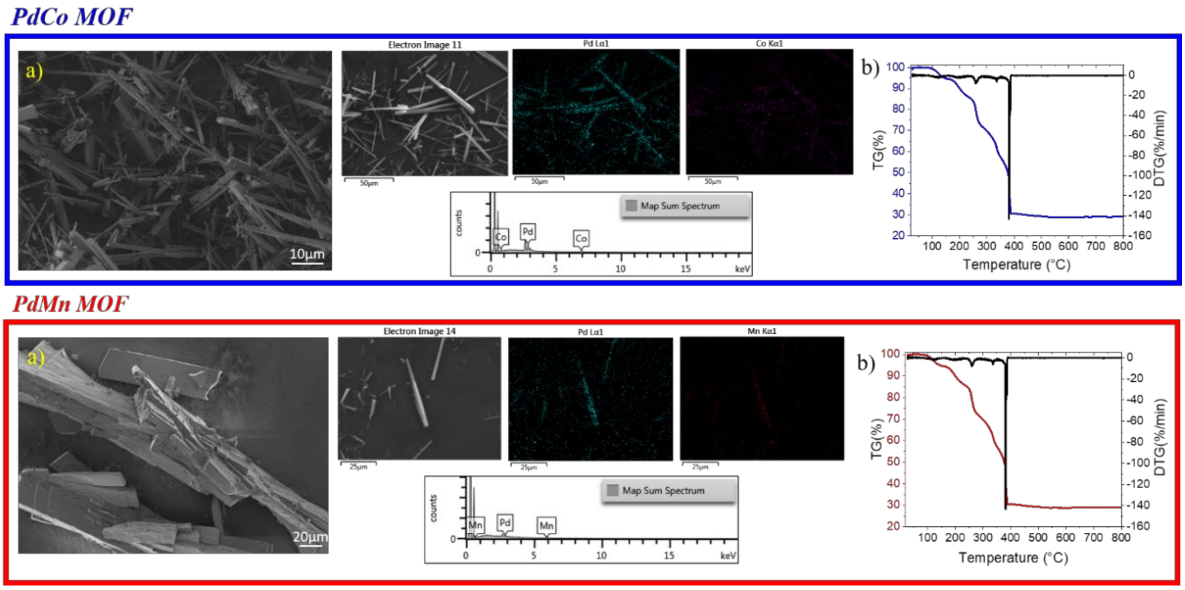

Figure S5. PdM-MOF characterization (M=Co or Mn). a) SEM picture showing nanocrystals and the corresponding EDS analysis of Pd, M, C, N, and O elements. b) **Black**: Thermogravimetric analysis (TGA) using a heating rate of 25 $^{0}C \cdot min^{-1}$ under airflow. **red**: The derivative of weight loss with temperature.

- **Additional PdM-Q and QT characterization**

To obtain the **PdCo-Q** and **PdMn-Q** nanocomposites, the MOF precursors were modified under the previously reported chemical treatment involving the in-situ generation of aniline in the presence of H_2 at room temperature. This treatment leads to Pd nanoparticle formation onto a carbonaceous support that still preserves most of the first-raw transition metal in an oxidized state. These composites serve as precursors for the **PdCo-QT** and **PdMn-QT** catalysts. Their corresponding chemical compositions are presented in the table below.

Table S2. Chemical composition of the PdM-Q materials.

Material	Pd wt%[a]	M	M wt%[a]	Pd/M molar ratio	N wt%[b]	C wt%[b]	H wt%[b]
PdCo-Q	**20.9**	**Co**	**11.5**	**1 :1**	**3.7**	**26.1**	**1.7**
PdMn-Q	**24.5**	**Mn**	**17.6**	**1 :1.3**	**4.5**	**27.0**	**2.2**

[a] By ICP, [b] By EA.

In Figure S6, very narrowly distributed and small nanoparticles can be distinguished by HR-HAADF-STEM imaging: 1.7 ± 0.6 nm for the **PdCo-Q** material and 1.8±0.8nm for **PdMn-Q** material. In order to determine these crystal structures, XRD spectra and derived data are presented in Figure S7 and Table S4. First, note the total disappearance of the MOF signal after the chemical treatment in **PdCo-Q** pattern. In fact, **PdCo-Q** shows a typical pattern of Co-doped Pd fcc nanoparticles (JCPDS 01-087-0637) with peaks observed at 40.1°, 46.8° and 82.0° corresponding to (111), (200) and (311) planes respectively. Following this observation, the calculated lattice constant parameter of 3.89Å agrees with the referenced cobalt-doped Palladium JCPDS card and the XAS results. Additionally, the peaks detected at 18.1° and 33.9° are consistent with (111) and (311) planes of $CoCo_2O_4$ fcc crystal (JCPDS 01-080-1541). On the other hand, **PdMn-Q** material showed a complex pattern with a clear signal from Pd fcc NPs (JCPDS 98-006-0960) at 39.5° (111), along with a mixture of peaks from Mn_3O_4 orthorhombic and tetragonal crystals. In this sense, peaks at 15.1°, 19.2°, 25.1°, 40.8°, and 42.0° are attributed to the tetragonal phase (JCPDS 03-065-2776) corresponding to (101), (200), (211), (321) and (204) planes respectively, while the peaks at 18.0°, 23.8°, 29.3°, and 35.1° are indexed to (002), (102), (120), and (202) planes of the orthorhombic phase (JCPDS 00-016-0350).

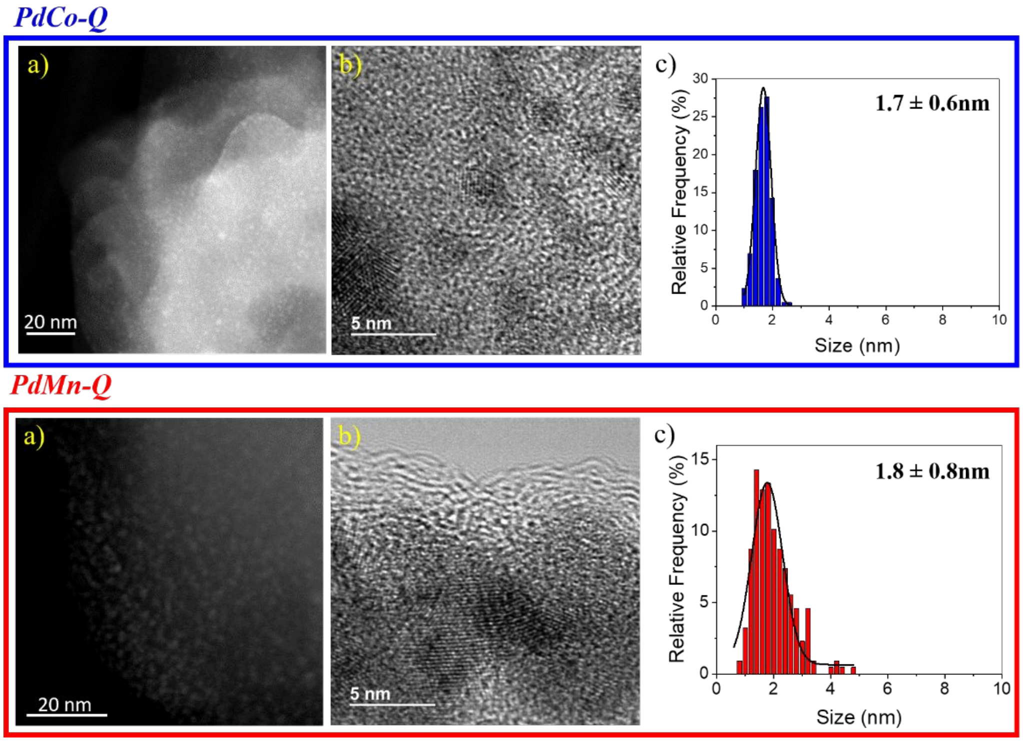

Figure S6. Electron microscopy characterization of **PdCo-Q** (up panel) and **PdMn-Q** (bottom panel) a) Representative HAADF STEM images of the **PdM-Q** catalysts, b) Representative HR-TEM images of the **PdM-Q** catalysts and, d) nanoparticle size distributions.

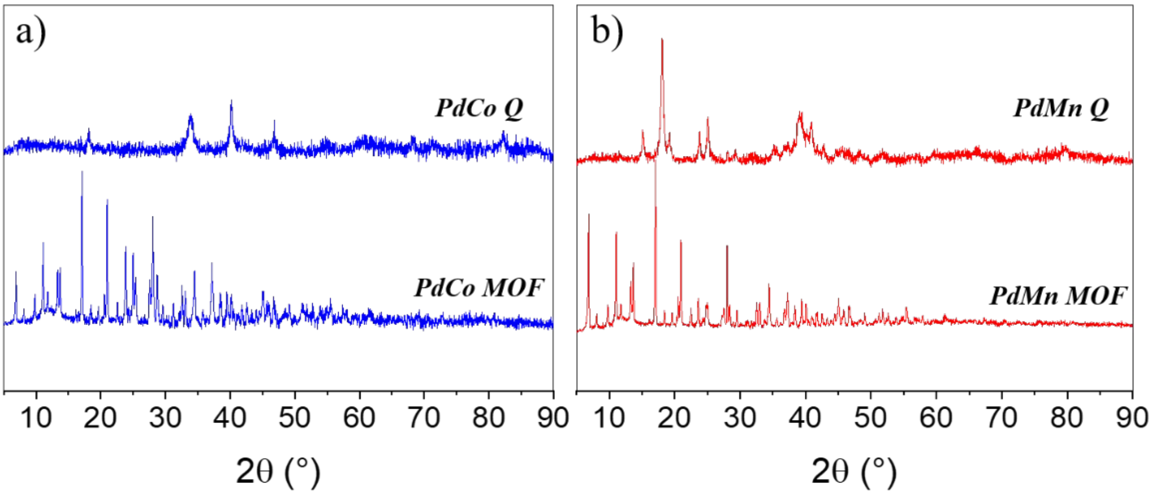

Figure S7. XRD patterns of PdM synthesized materials, a) **PdCo** and, b) **PdMn**.

As illustrated in Figure S8 and Table S3, Raman spectroscopy confirmed the graphitic nature of the carbon produced during the chemical process. The spectra from **PdCo-Q** and **PdMn-Q** display the characteristic G band of graphitic carbon at 1588 cm^{-1}.[5] In this instance, the ratio of intensities between

the D and G bands (I_D/I_G = 0.85 for **PdCo-Q** and 0.88 for **PdMn-Q**) indicate high defect concentration in both cases.

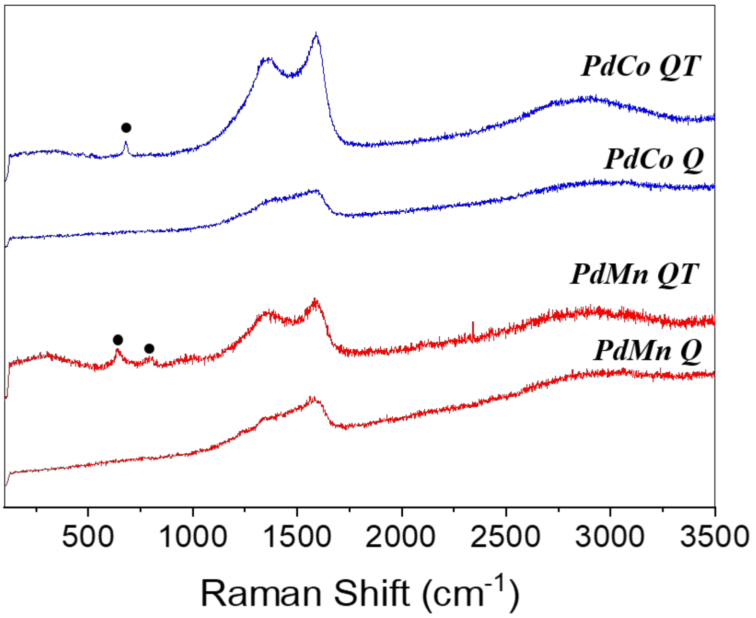

Figure S8. Raman spectra of **PdMn-Q** and **QT** (red) and **PdCo-Q** and **QT** (blue) materials.

Table S3. Detailed Raman shift of **PdMn-Q** and **QT** (red) and **PdCo-Q** and **QT** (blue) samples.

Material	D band positions (cm^{-1})	G band positions (cm^{-1})	I_D/I_G
PdCo-Q	**1356**	**1588**	**0.85**
PdMn-Q	**1356**	**1588**	**0.88**
PdCo-QT	**1356**	**1588**	**0.83**
PdMn-QT	**1356**	**1588**	**0.86**

Table S4. Resumed data extracted from XRD patterns of **PdM-Q** and **QT (M=Co, Mn).**

Material	Crystalline phases	[a]crystal average size (nm)	Lattice constant (Å)
PdCo-Q	**[1]Co doped Pd (fcc) / [2]$CoCo_2O_4$ (fcc)**	**n.a.**	**[1]3.89/ [2]n.a.**
PdMn-Q	**Mn_3O_4 (orthorombic and tetragonal) /Pd (fcc)**	**n.a.**	**n.a.**
PdCo-QT	**[1]PdCo (fcc) / [2]PdCo (fcc)**	**[1]18 / [2]22**	**[1]3,83 / [2]3.94**
PdMn-QT	**PdMn (Tg)**	**22**	**a=b=2.87-c=3.58**

[a]Calculated by Debbye Scherrer equation. n.a.: not applicable

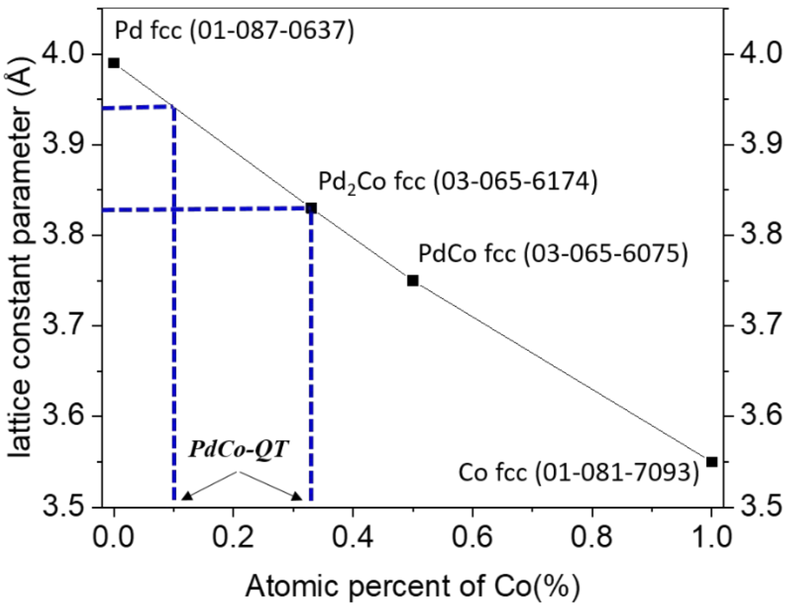

Figure S9. Theoretical plot of lattice constant parameter of PdCo fcc crystal system as a function of the atomic composition.

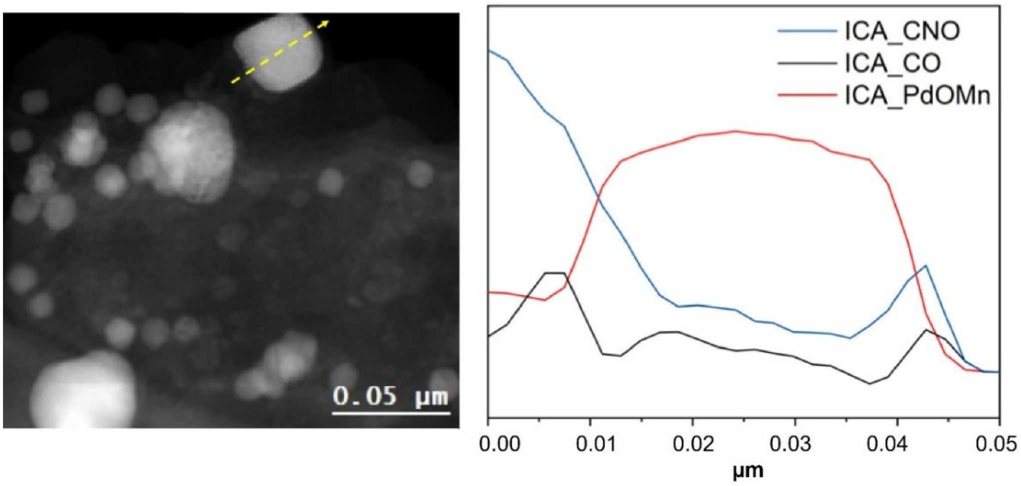

Figure S10. Intensity profiles on the different ICA components along the path marked with a yellow arrow on the HAADF-STEM image.

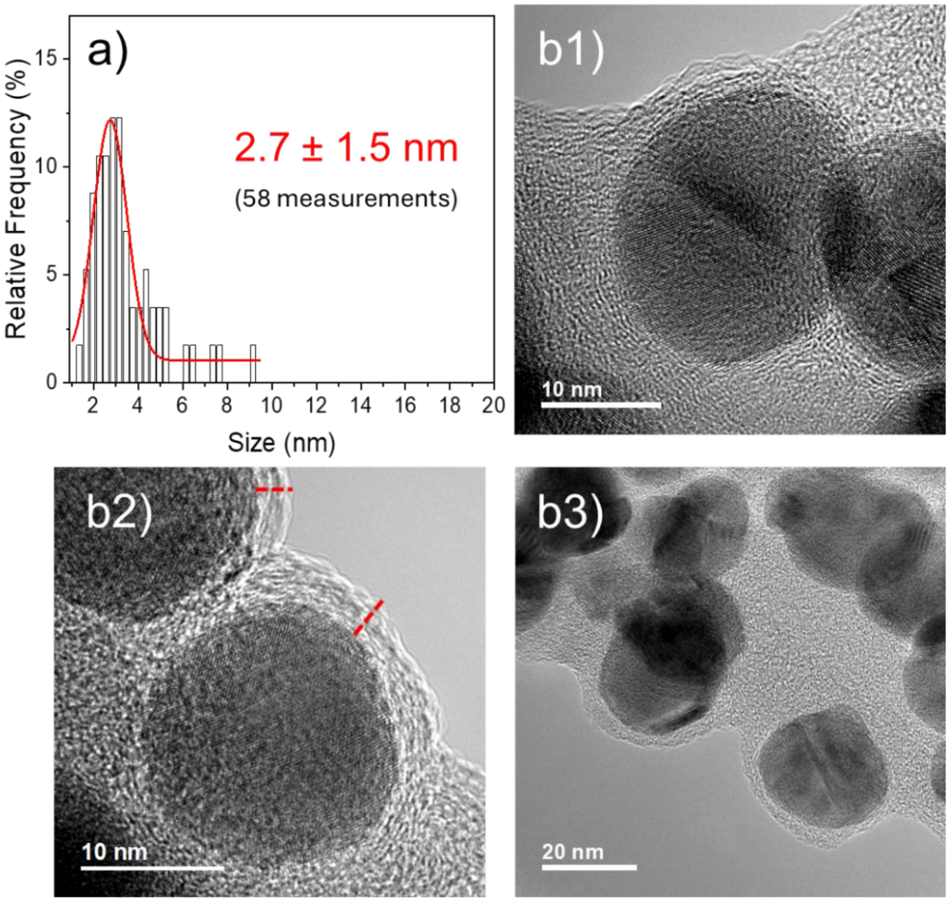

Figure S11. Carbonaceous covering on PdCo-QT sample: a) Layer thickness distribution. b) Representative HR-TEM images.

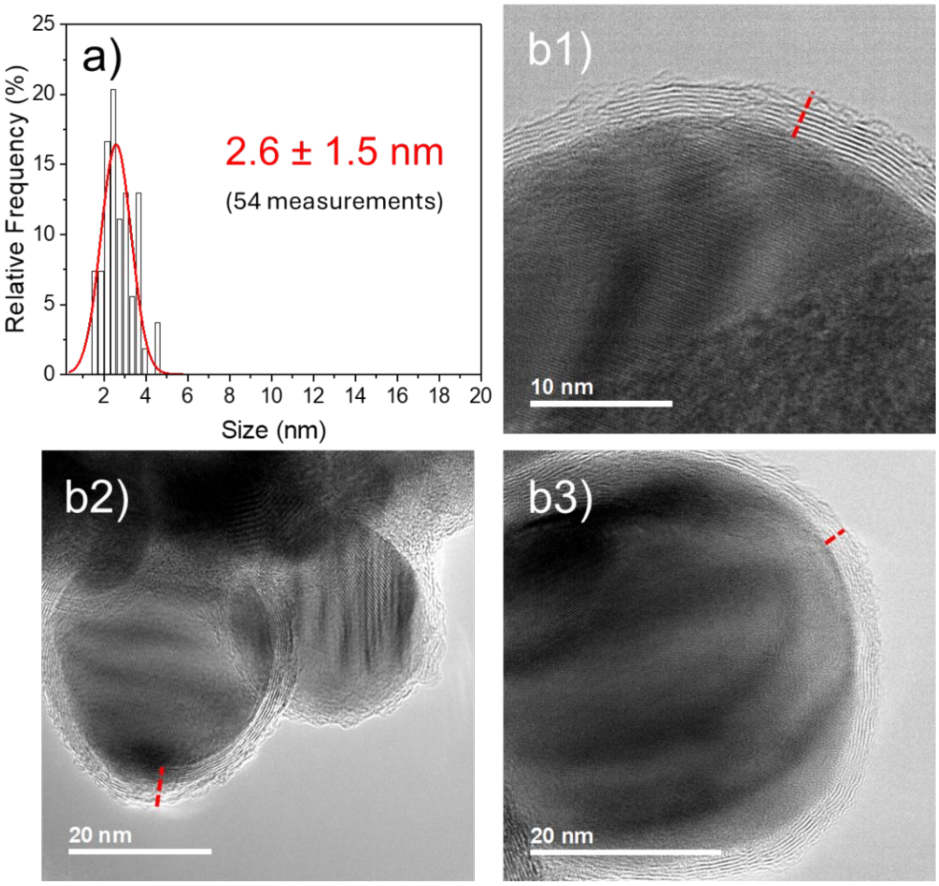

Figure S12. Carbonaceous covering on PdMn-QT sample: a) Layer thickness distribution. b) Representative HR-TEM images.

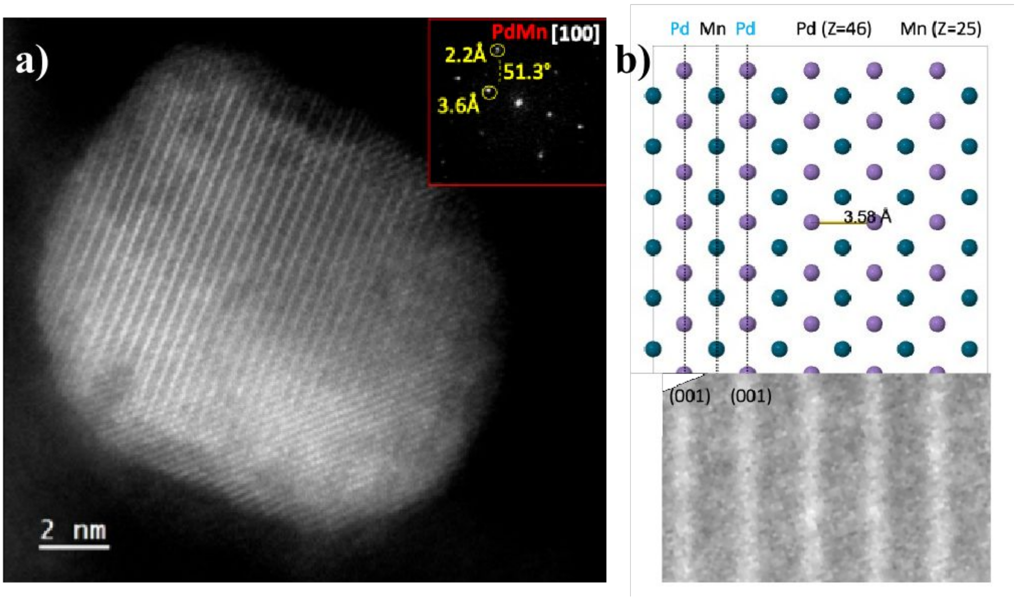

Figure S13. HR-HAADF-STEM study evidencing Pd and Mn ordering into a PdMn intermetallic. a) HR-STEM-HAADF image and Digital Diffractogram (inset). b) Structural model for the [100] projection of PdMn intermetallic phase and comparison against the contrast alternation observed in a).

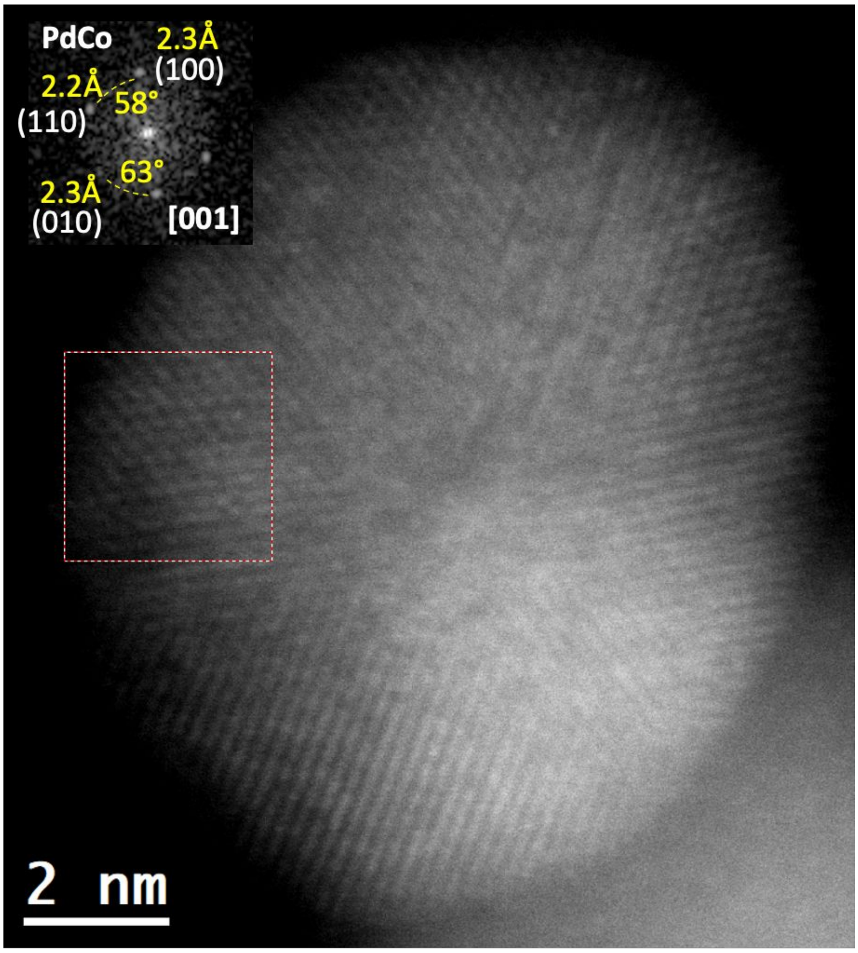

Figure S14. STEM HAADF image of the **PdCo-QT** (after 5th run) sample. Indexed Digital Diffractogram shown as inset.

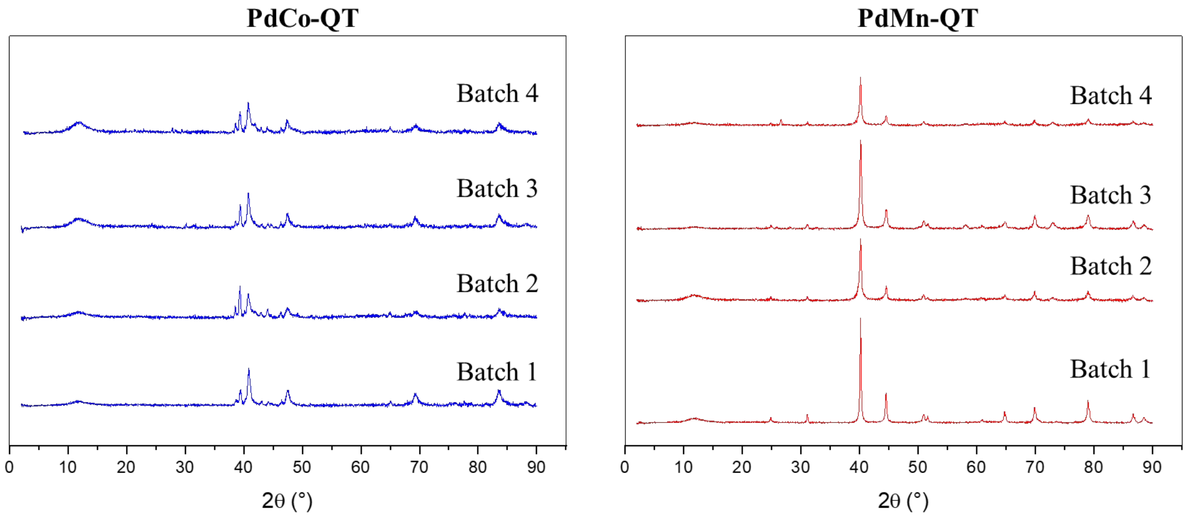

Figure S15. XRD pattern of **PdCo-QT** and **PdMn-QT** catalysts from several batch syntheses.

PdCo – Pd edge

Table S5. Summary of EXAFS Fits of **PdCo-QT** Sample Performed on Pd edge.

Sample	CN		σ^2 (Å^2)	Distance (Å)		ΔE_0	R-factor
	Co	Pd		Co	Pd		
PdCo-QT	4.9±0.2	4.9±0.3	0.0062±0.0003	2.608±0.004	2.681±0.003	2.2±0.6	0.004

The fits were performed on the first coordination shell (ΔR = 1.0-3.0 Å) over FT of the k^2-weighted χ(k) functions performed in the Δk = 3.6-14.0 Å^{-1} interval, resulting in a number of independent parameters of 2ΔRΔk/π=12.9 for each spectrum. S_0^2 = 0.9 from Pd foil.

Table S6. Summary of EXAFS Fits of **PdCo-MOF** and **PdCo-Q** Samples Performed on Pd edge.

Sample	CN			σ^2 (Å^2)	Distance (Å)			ΔE_0	R-factor
	O	Cl	Pd		O	Cl	Pd		
PdCo-MOF	1.4±0.3	1.4±0.3	-	0.0015±0.0016	2.006±0.022	2.307±0.285	-	4.5±2.4	0.010
PdCo-Q	0.9±0.6	2.0±0.4	3.3±0.9	0.0064±0.0014	1.988±0.055	2.285±0.018	2.740±0.009	0.3±1.8	0.021

The fits were performed on the first coordination shell (ΔR = 1.0-2.2 Å for PdCo-MOF and 1.0-3.0 Å for PdCo-Q) over FT of the k^2-weighted χ(k) functions performed in the Δk = 3.6-14.0 Å^{-1} interval, resulting in a number of independent parameters of 2ΔRΔk/π=7.7 for PdCo-MOF and 12.9 for PdCo-Q. S_0^2 = 0.9 from Pd foil.

PdMn – Pd edge

Table S7. Summary of EXAFS Fits of **PdMn-QT** Sample Performed on Pd edge.

Sample	CN		σ^2 (Å^2)	Distance (Å)		ΔE_0	R-factor
	Mn	Pd		Mn	Pd		
PdMn-QT	6.8±0.2	4.1±0.3	0.0076±0.0003	2.687±0.004	2.848±0.003	2.2±0.5	0.0007

The fits were performed on the first coordination shell (ΔR = 1.4-3.0 Å) over FT of the k^2-weighted χ(k) functions performed in the Δk = 3.6-14.0 Å^{-1} interval, resulting in a number of independent parameters of 2ΔRΔk/π=10.3 for each spectrum. S_0^2 = 0.9 from Pd foil.

Table S8. Summary of EXAFS Fits of **PdMn-MOF** and **PdMn-Q** Samples Performed on Pd edge.

Sample	CN			σ^2 (Å^2)	Distance (Å)			ΔE_0	R-factor
	O	Cl	Pd		O	Cl	Pd		
PdMn-MOF	1.5±0.3	1.3±0.3	-	0.0013±0.0017	2.007±0.023	2.307±0.287	-	4.7±2.5	0.011
PdMn-Q	0.4±0.4	2.2±0.3	4.2±0.7	0.0066±0.0009	1.996±0.083	2.284±0.012	2.740±0.006	1.0±1.2	0.010

The fits were performed on the first coordination shell (ΔR = 1.0-2.2 Å for JMM6 and 1.0-3.0 Å for PdMn-Q) over FT of the k^2-weighted χ(k) functions performed in the Δk = 3.6-14.0 Å^{-1} interval, resulting in a number of independent parameters of 2ΔRΔk/π=7.7 for PdMn-MOF and 12.9 for PdMn-Q. S_0^2 = 0.9 from Pd foil.

PdCo – Co edge

Table S9. Summary of EXAFS Fits of **PdCo-MOF**, **PdCo-Q**, and **PdCo-QT** Samples Performed on Co edge.

Sample	CN			σ^2 (Å^2)	Distance (Å)			ΔE_0	R-factor
	O	Co	Pd		O	Co	Pd		
PdCo-MOF	6.0±0.8	-	-	0.0066±0.0019	2.090±0.013	-	-	0.04±1.3	0.006
PdCo-Q	6.3±0.4	-	-	0.0072±0.0009	2.087±0.006	-	-	-0.1±0.6	0.002
PdCo-QT	-	3.1±0.1	4.5±0.1	0.0079±0.0003	-	2.542±0.003	2.602±0.003	4.8±0.2	0.001

The fits were performed on the first coordination shell (ΔR = 1.6-3.3 Å for PdCo-QT and 1.1-2.0 Å for the rest of samples) over FT of the k^2-weighted χ(k) functions performed in the Δk = 2.0-12.0 Å^{-1} interval, resulting in a number of independent parameters of 2ΔRΔk/π=9.9 for PdCo-QT and 5.6 for the rest of samples. S_0^2 = 0.8 from Co foil.

PdMn – Mn edge

Table S10. Summary of EXAFS Fits of **PdMn-MOF**, **PdMn-Q**, and **PdMn-QT** Samples Performed on Mn edge.

Sample	CN			σ^2 (Å^2)	Distance (Å)			ΔE_0	R-factor
	O	Pd	Mn		O	Pd	Mn		
PdMn-MOF	4.0±0.4	-	-	0.0056±0.0013	2.147±0.009	-	-	-3.5±1.0	0.0052
PdMn-Q	2.9±0.6	-	-	0.0068±0.0031	2.143±0.022	-	-	-7.4±2.5	0.0252
PdMn-QT	-	3.2±0.5	1.2±0.4	0.0043±0.0009	-	2.695±0.007	2.815±0.026	2.1±0.8	0.0045

The fits were performed on the first coordination shell (ΔR = 1.8-3.0 Å for PdMn-QT and 1.0-2.2 Å for the rest of samples) over FT of the k^2-weighted χ(k) functions performed in the Δk = 2.5-12.0 Å^{-1} interval, resulting in a number of independent parameters of 2ΔRΔk/π=7.0 for each spectrum. S_0^2 fixed to 1.0.

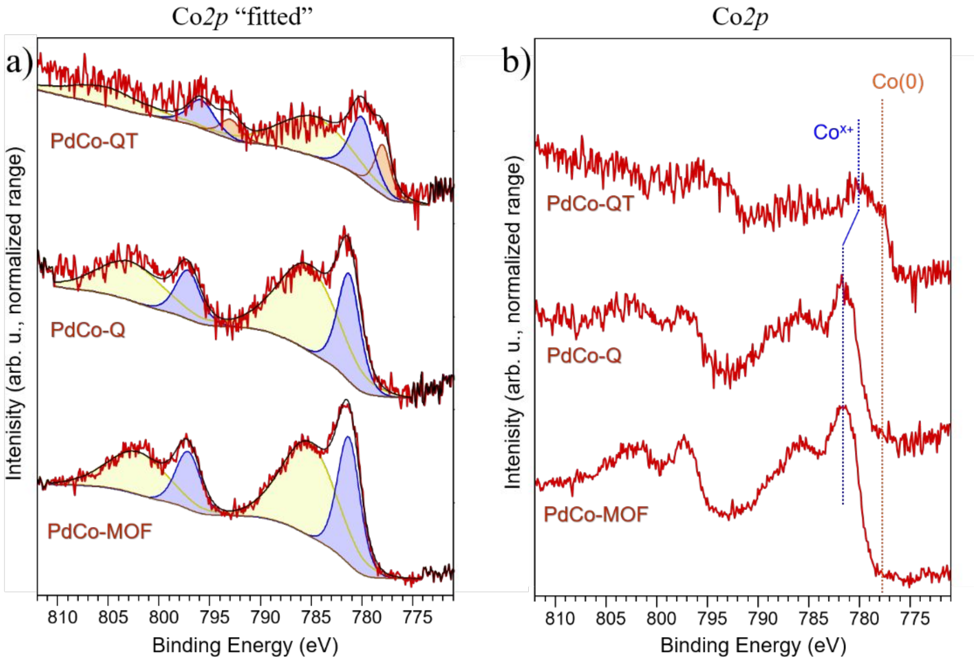

Figure S16. a) Co2*p* *"fitted"*, and b) Co2*p*, XPS regions of MOF-derived PdCo samples.

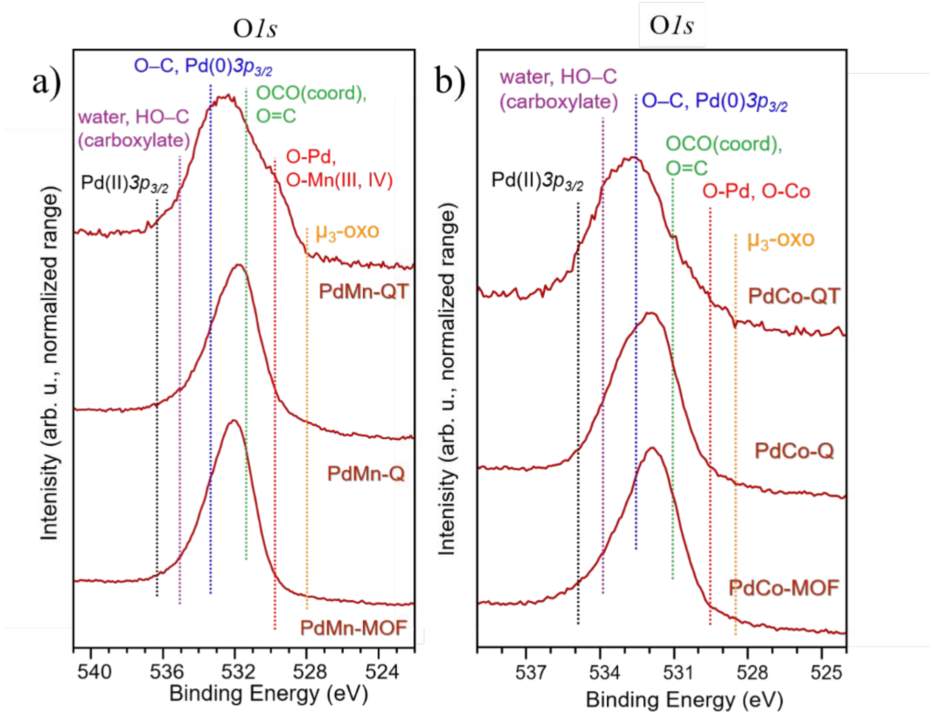

Figure S17. Complex O*1s* XPS regions of MOF-derived PdM samples, a) Mn and b) Co.

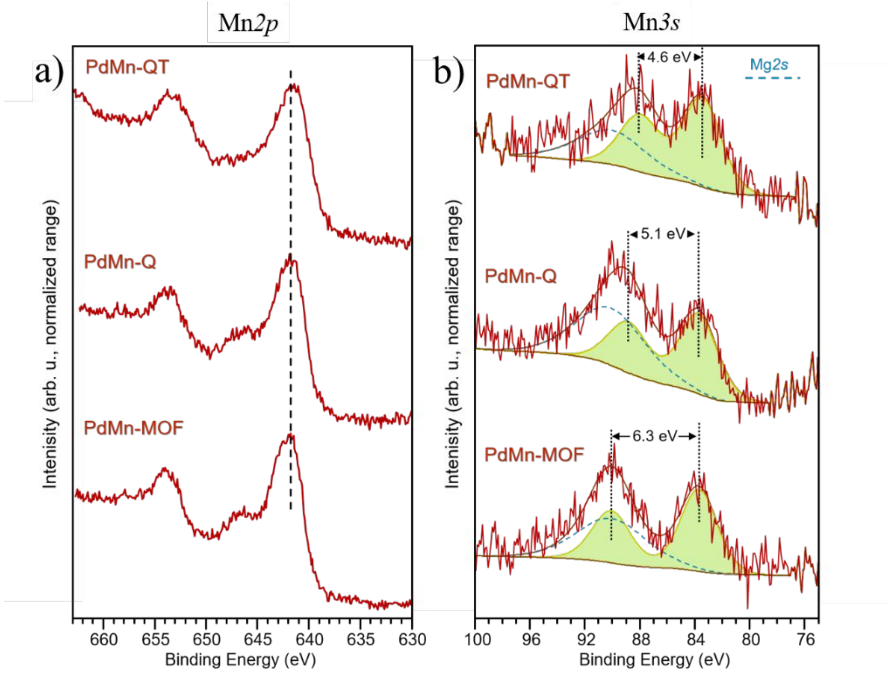

Figure S18. a) Mn*2p*, and b) Mn3s, XPS regions of MOF-derived PdMn samples.

In the case of the Mn*2p* XP region, the progressive disappearance of the satellite at ca. 647 eV suggests an increase in the manganese oxidation state through the chemical and thermal process (Figure S13a). Interestingly, the exchange splitting of the Mn*3s* core-level signal due mainly to the exchange interaction between the *3s* and *3d* electrons can confirm this phenomenon. The values of Mn average oxidation can be obtained, as suggested by Beyreuther *et al.*[6] considering this doublet separation from the following equation:

$$\boldsymbol{\upsilon_{Mn} = 9.67 - 1.27\Delta E_{3s}/eV}$$

where $\boldsymbol{\upsilon_{Mn}}$ is the average oxidation state and ΔE_{3s} the splitting value of the Mn*3s* core level. The values of Mn average oxidation state in **PdMn-MOF**, **PdMn-Q** and **PdMn-QT** are gathered in Table S11. In summary, the binding energy difference between the doublet composing the Mn*3s* XP region decreases after the chemical and thermal treatments on the **PdMn-MOF**. Consequently, the average manganese oxidation state becomes higher, which is also in agreement with Mn*2p* observations. However, the extreme oxophilicity of Mn and the manipulation of these samples under ambient conditions disallow any judgment regarding the generation of these oxidized species.

Table S11. Detailed values of Mn*3s* XP regions for PdMn-derived materials.

Material	Mn 3s (^{5}S) / eV	Mn 3s (^{7}S) / eV	ΔMn 3s / eV	υMn
**PdMn-MOF**	90.0	83.7	6.3	1.7
**PdMn-Q**	88.8	83.7	5.1	3.2
**PdMn-QT**	88.0	83.4	4.5	4.0

- **PdM-MOF and PdM-Q activity/stability**

Table S12. Activity and selectivity comparison of various catalysts in phenylacetylene hydrogenation.

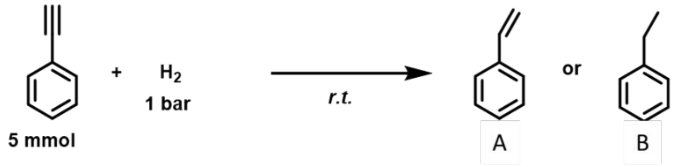

Entry	Catalyst	Time (h)	Conv. (%)	Selec. to A (mol.%)	TON	TOF (h^{-1})	Productivity ($g_{alkene} \cdot g_{cat}^{-1} \cdot h^{-1}$)
**1**	***PdCo-MOF***	**7**	**25**	**91**	**34.1**	**4.9**	**1.2**
**2**	***PdCo-Q***	**3**	**99**	**70**	**161.9**	**53.9**	**15.4**
**3**	***PdMn-MOF***	**7**	**68**	**87**	**79.6**	**11.4**	**2.7**
**4**	***PdMn-Q***	**2**	**99**	**78**	**135.5**	**67.8**	**33.8**

Reaction Conditions: 5mmol of phenylacetylene, substrate/Pd molar ratio: 323/1, 5mL EtOH, *r.t,*1000rpm, 1bar H_2. TON= mol of converted alkyne/mol of metal,TOF=TON/time.

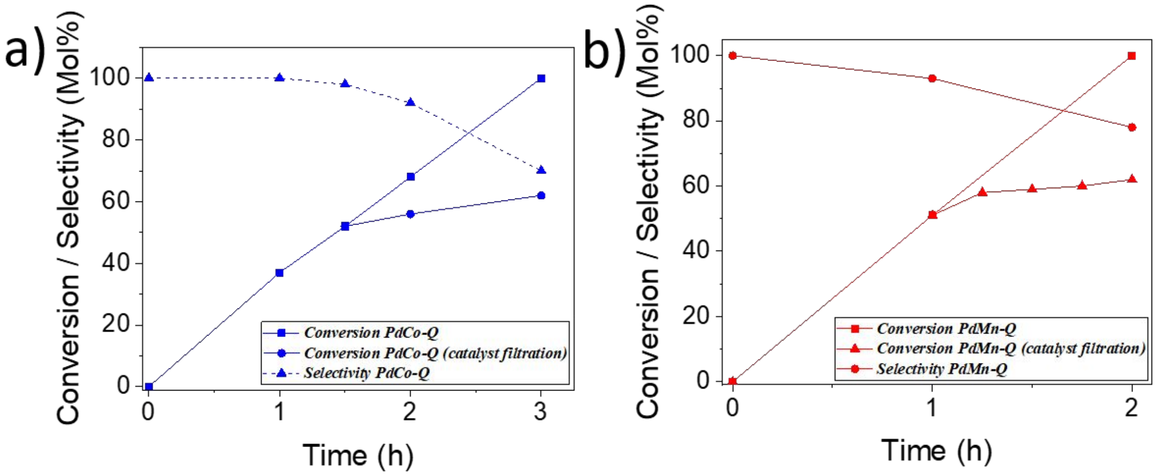

Figure S19. Catalyst filtration of a) **PdCo-Q** and b) **PdMn-Q** catalyst. Reaction Conditions: 5 mmol of phenylacetylene, substrate/Pd molar ratio: 323/1, 5 mL EtOH, *r.t.*, 1 bar H_2, 1000 rpm.

Table S13. ICP analysis results of the final filtrated reaction crude for **PdCo-Q** and **PdMn-Q** materials.

Material	Pd leaching (wt%)	Pd leaching (ppm)	M	M leaching (wt%)	M leaching (ppm)
PdCo-Q	0.55	**2.30**	**Co**	0.54	**1.24**
PdMn-Q	0.16	**0.66**	**Mn**	0.45	**1.35**
Pd/C commercial	0.4	**1.67**	**-**	-	

Note: wt% values refer to the entire catalyst quantity used.

The HR-HAADF images of the nanomaterials after the hydrogenation process show an increase in nanoparticle size, as well as some agglomeration of the nanoparticles (Figure S15). Indeed, the average particle size for **PdMn-Q-run1** is 8.8 ± 5.9 nm, significantly larger when compared to the 1.8 ± 0.8nm estimated for the fresh material. On the other hand, the **PdCo-Q-run1** clearly displays a bimodal type nanoparticle size distribution, with two populations, with average particle sizes of 1.3 ± 0.5nm and 5.0 ± 1.9nm. Note that only the first set of NPs shows a size comparable to that observed before catalysis, 1.7 ± 0.6nm.

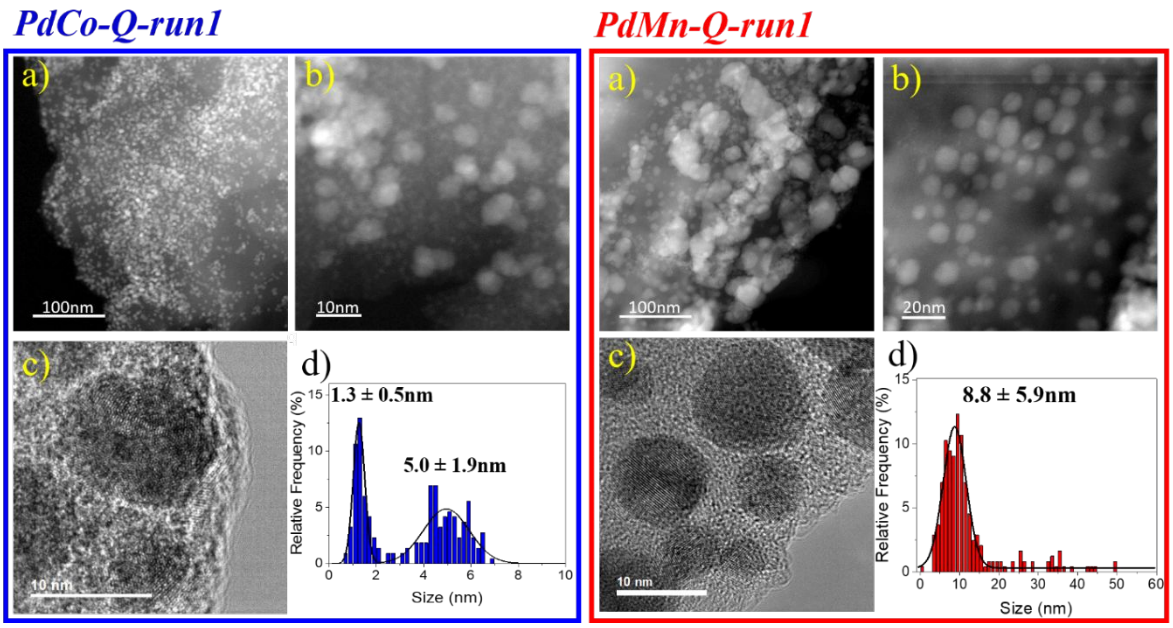

Figure S20. Microscopy results for **PdCo-Q** (blue panel) and **PdMn-Q** (red panel) after 1 catalytic cycle. a), b) HAADF-STEM image and d) the corresponding nanoparticle size distribution, and c) a representative HRTEM image.

In addition, Figure S16 and Table S14 summarize the XRD comparison of **PdCo-Q** and **PdMn-Q** before and after catalysis. Both materials' patterns after catalysis indicate a drastic increase concerning the monometallic Pd fcc phase peaks. Precisely, Pd signals at 40°, 46°, 68°, 81° and 86° from (111), (200), (220), (311), and (222) reflections planes suggest an average crystal size of 13 nm for **PdCo-Q-run1** and 27 nm for **PdMn-Q-run1**. In agreement with microscopy results, these observations indicate a drastic nanoparticle size increase. Furthermore, in the case of the **PdMn-Q** catalyst, the orthorhombic Mn_3O_4 phase has not been detected after the catalysis, with a complete disappearance of the peak at 18°. Also, in the case of the **PdCo-Q** catalyst, additional peaks at 18°, 21°, and 25° corresponding to a monoclinic CoO_2 phase are detected after catalysis (JCPDS 01-070-3469). Concurrently, the typical $CoCo_2O_4$ peak at 34° disappeared after catalysis.

In conclusion, the drastic modifications under catalytic conditions of the chemically MOF-derived materials have been demonstrated. Those modifications indicate a poor stability of these materials and a high mobility of the active species, in agreement with the ICP results discussed in the main text (section: *catalytic results/* subsection*: PdCo-PdMn catalytic stability*). Despite the interesting activity, the additional thermal step of our methodology is imperative to modulate the activity and, specially, to improve the stability towards an ideal and robust catalyst.

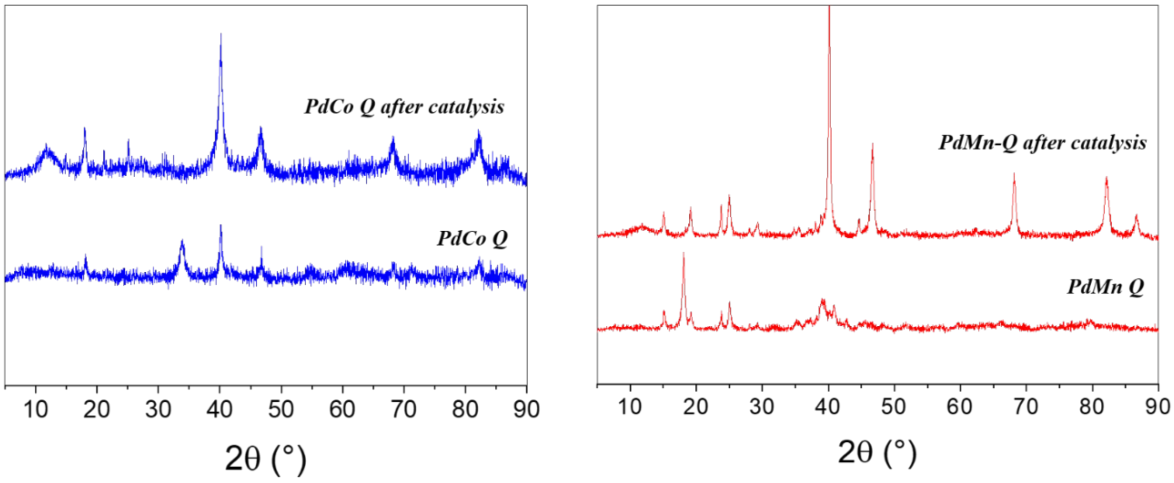

Figure S21. XRD patterns of **PdCo-Q** fresh and after 1 cycle (left panel), **PdMn-Q** fresh and after 1 cycle.

Table S14. Summarized data extracted from XRD patterns of **PdM-Q** and **PdM-Q after catalysis** (M=Co, Mn).

Material	Crystalline phases	[a]Av. crystal size (nm)	Lattice constant (Å)	Material	Crystalline phases	[a]Av. crystal size (nm)	Lattice constant (Å)
PdCo-Q - fresh	[1]Co-doped Pd (fcc) / [2]$CoCo_2O_4$ (fcc)	n.a.	[1]3.89/ [2]n.a.	*PdMn-Q - fresh*	Pd (fcc)/ Mn_3O_4 (orthorhombic and tetragonal)	n.a.	n.a.
PdCo-Q- after catalysis	[1]Co doped (fcc) / [2]CoO_2 (monoclinic)	[1]13.0/ [2]n.a.	[1]3.88/ [2]n.a.	*PdMn-Q – after catalysis*	[1]Pd (fcc) / [2]Mn_3O_4 (tetragonal)	[1]27/ [2]n.a.	[1]3.89/ [2]n.a.

[a]Calculated by Debbye Scherrer equation. n.a.: Not applicable

PdM-QT activity/stability

Table S15. State of the art of Pd-based monometallic and bimetallic catalysts in selective hydrogenation of phenylacetylene under similar reaction conditions to those used in this work.

Catalyst	T (°C)	Solvent	P (bar)	C (mol%)	S (mol%)	TON	TOF (h^{-1})	Productivity ($g_{alkyne} \cdot g_{cat}^{-1} \cdot h^{-1}$)	Ref
Pd_2Ni_2/NC	*r.t.*	EtOH	1	98	95	371	279	14.6	7
PdNi@γAl_2O_3	25	EtOH	1	98	94	1485	2250	29.0	8
PdIn@Mg Al_2O_3	*r.t.*	Hexane	1	92	97	127	36	1.3	9
PdPb/C	30	CH_3CN	1	97	98	465	232	9.0	10
PdCu/NPCNs	25	EtOH	1	97	93	230	173	7.1	11
Pd-NC@NC	28	EtOH	1	68	97	73	73	0.7	12
PdMn-NC	60	n-hexane	15	99	95	6378	1063	10.9	13
Pd_6-TiO_2	50	EtOH	10	95	94	820	987	4.6	14
**PdIn-QT-RUN 5**	*r.t.*	EtOH	1	96	95	150	21	9.4	4
**PdMn-QT-RUN 5**	***r.t.***	**EtOH**	**1**	**78**	**97.5**	**109**	**15.6**	**12.6**	**This work**
**PdCo-QT-RUN 5**	***r.t.***	**EtOH**	**1**	**89**	**97.1**	**144**	**20.6**	**11.0**	**This work**

Note: Aimed at establishing meaningful comparison and due to the lack of complete kinetic data, TON, TOF, and productivity values were all calculated at the highest level of conversion and selectivity reported for each catalyst under consideration.

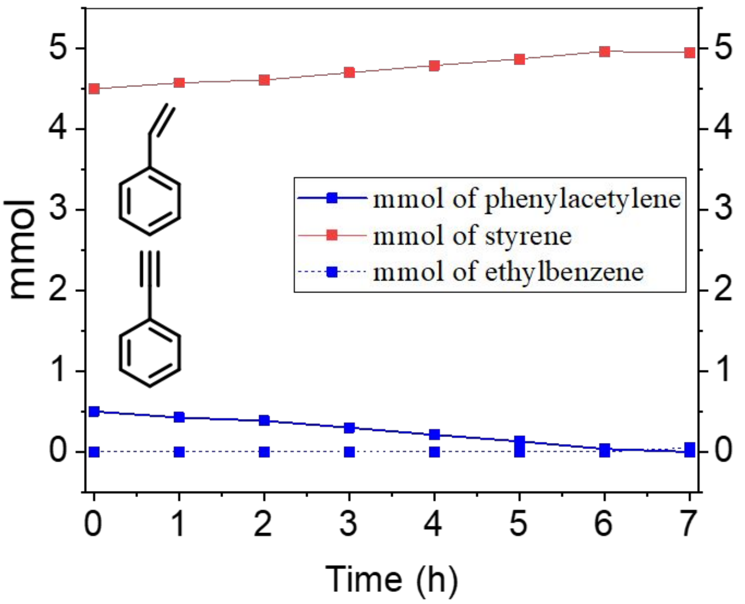

Figure S22. Alkene and alkyne conversion with **PdCo-QT** catalyst during an experiment with a substrate mix (9:1 eq respectively). Reaction Conditions: 4.5 mmol of phenylacetylene, 0.5 mmol of styrene substrate/Pd molar ratio: 323/1, 5 mL EtOH, r.t., 1 bar H_2, 1000 rpm.

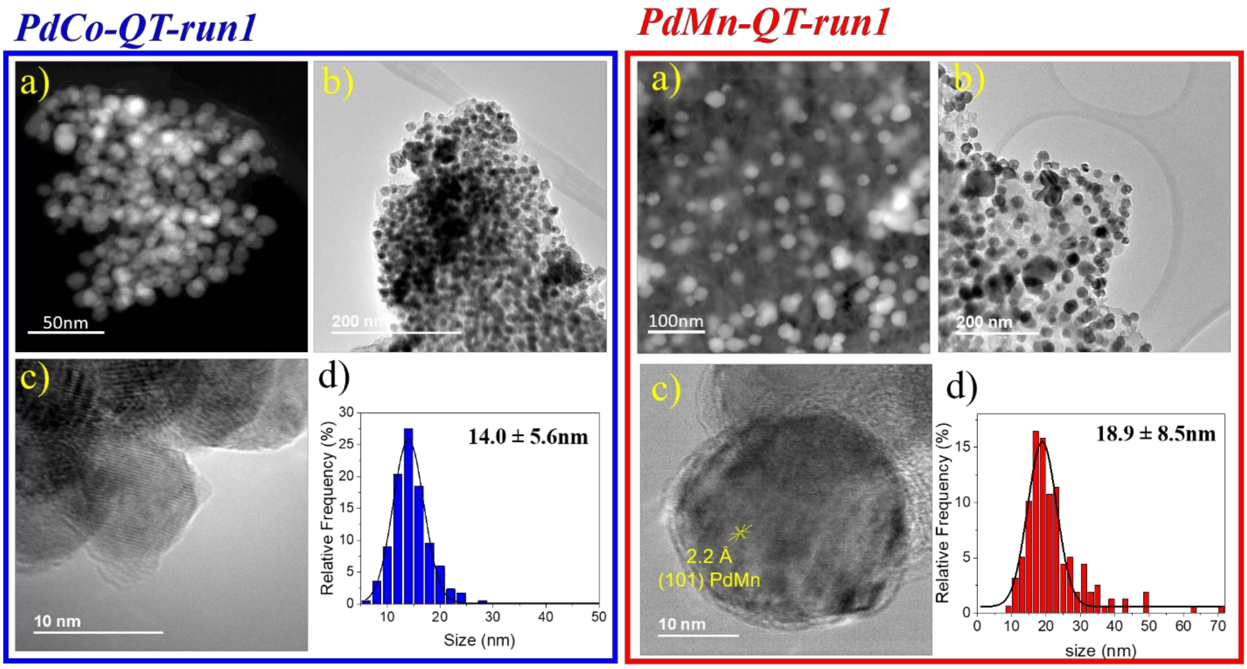

Figure S23. Microscopy results for **PdCo-QT** (blue panel) and **PdMn-QT** (red panel) after 1 catalytic cycles. a), b) HAADF-STEM image, d) the corresponding nanoparticle size distribution, and c) HRTEM image with measured interplanar distances.

Table S16. ICP analysis results of the final filtrated reaction crude for **PdCo-QT** and **PdMn-QT** materials.

Material	Pd leaching (wt%)	Pd leaching (ppm)	M	M leaching (wt%)	M leaching (ppm)
PdCo-QT	0.03	**0.13**	**Co**	0.50	**1.16**
PdMn-QT	0.08	**0.33**	**Mn**	1.00	**2.86**
Pd/C commercial	0.40	**1.67**	**-**	-	**-**

Note: wt% values refer to the entire catalyst quantity used.

Table S17. ICP analysis results of the final filtrated reaction crude for **PdCo-QT** materials after 1 to 5 cycles.

Run	Pd (wt%)	Co (wt%)
**1**	0.03	0.50
**2**	0.02	0.34
**3**	0.01	0.26
**4**	0.01	0.07
**5**	0.14	0.12

Note: wt% values refer to the entire catalyst quantity used.

Table S18. ICP analysis results of the final filtrated reaction crude for **PdMn-QT** materials after 1 to 5 cycles.

Run	Pd (wt%)	Mn (wt%)
**1**	0.08	1.0
**2**	0.1	0.35
**3**	0.5	0.1
**4**	0.02	0.1
**5**	0.03	0.34

Note: wt% values refer to the entire catalyst quantity used.

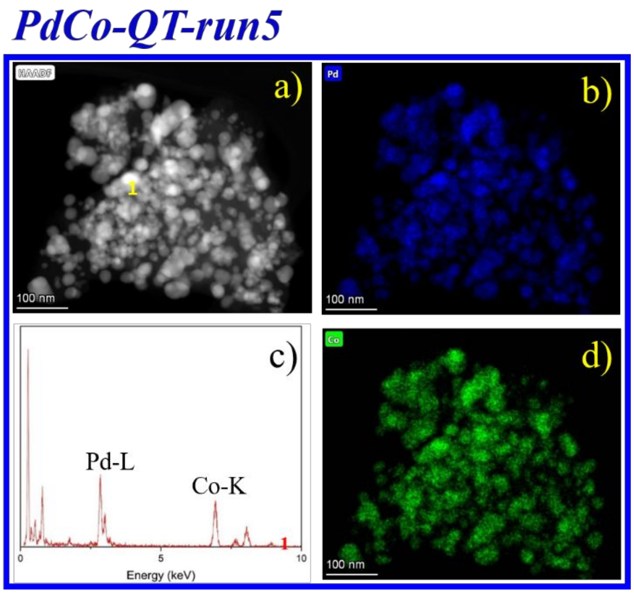

Figure S24. STEM-EDX **PdCo-QT** sample after run 5. a) HAADF image and the corresponding chemical map extracted from the STEM-SI-EDS b) Pd and d) Co, and c) a representative EDS spectrum.

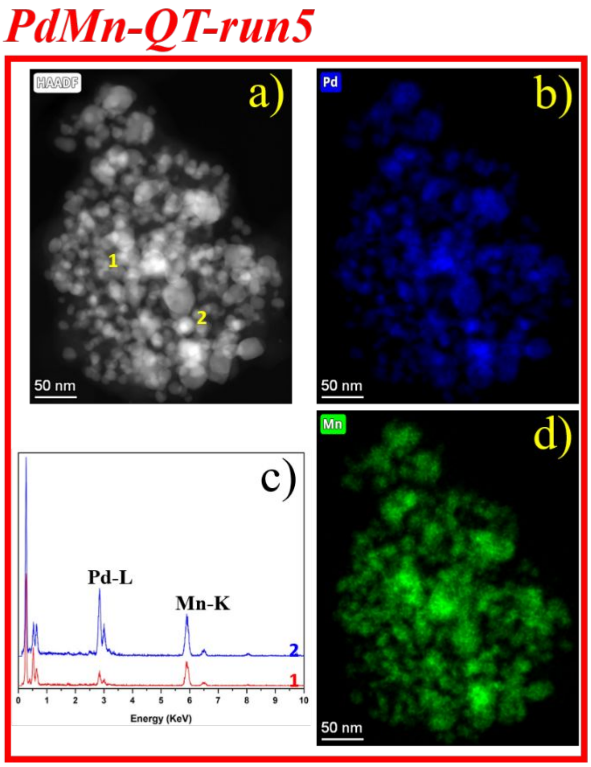

Figure S25. STEM-EDX **PdMn-QT** sample after run 5. a) HAADF image and the corresponding chemical map extracted from the STEM-SI-EDS b) Pd and d) Mn, and c) a representative EDS spectrum.

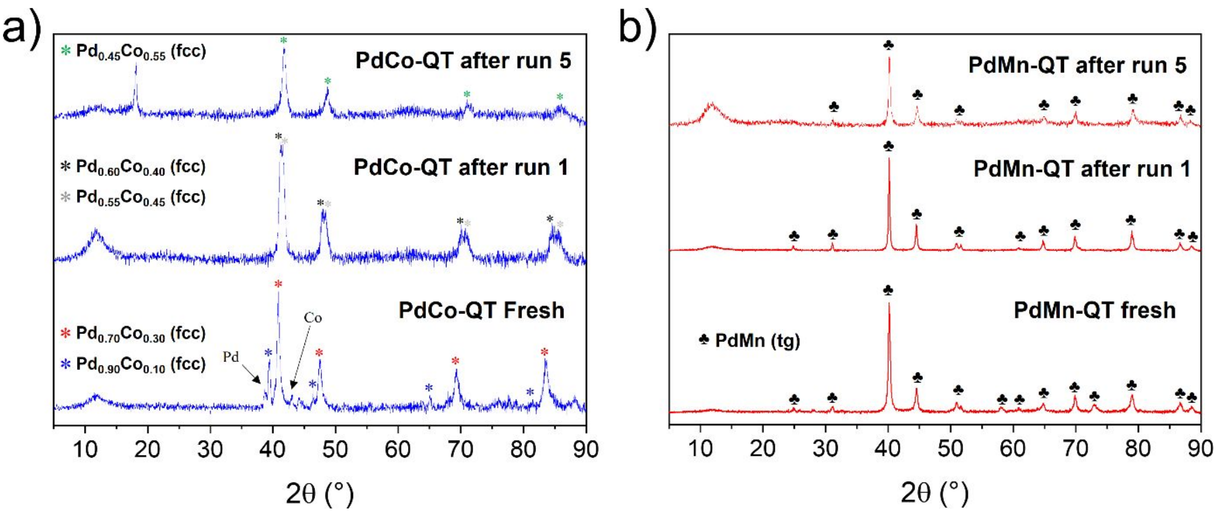

Figure S26. XRD patterns of a) **PdCo-QT** fresh, after 1 cycle, and after 5 cycles. b) **PdMn-QT** fresh, after 1, and 5 cycles.

Table S19. Resumed data extracted from XRD patterns of **PdM-QT** and **PdM-QT after 1** and **5 cycles (M=Co, Mn).**

Material	Crystalline phases	[a]crystal average size (nm)	Lattice constant (Å)	Material	Crystalline phases	[a]crystal average size (nm)	Lattice constant (Å)
PdCo QT-fresh	[1]$Pd_{0.66}Co_{0.33}$ (fcc) / [2]$Pd_{0.9}Co_{0.1}$ (fcc)	[1]18 / [2]22	[1]3,83 / [2]3.94	*PdMn QT-fresh*	PdMn (Tg)	22	a=b=2.87- c=3,58
PdCo QT-run 1	[1]$Pd_{0.6}Co_{0.4}$ (fcc)/ [2]$Pd_{0.55}Co_{0.45}$ (fcc)	[1]n.a. / [2]n.a.	[1]3,79 / [2]3.76	*PdMn QT-run 1*	PdMn (Tg)	30	a=b=2.87- c=3.58
PdCo QT-run5	$Pd_{0.45}Co_{0.55}$ (fcc)/ $CoCo_2O_4$ (fcc)	[1]13/[2]n.a.	[1]3.74/[2]n.a.	*PdMn QT-run5*	PdMn (Tg)	27	a=b=2.87- c=3.58

[a]Calculated by Debbye Scherrer equation. n.a.: Not applicable.

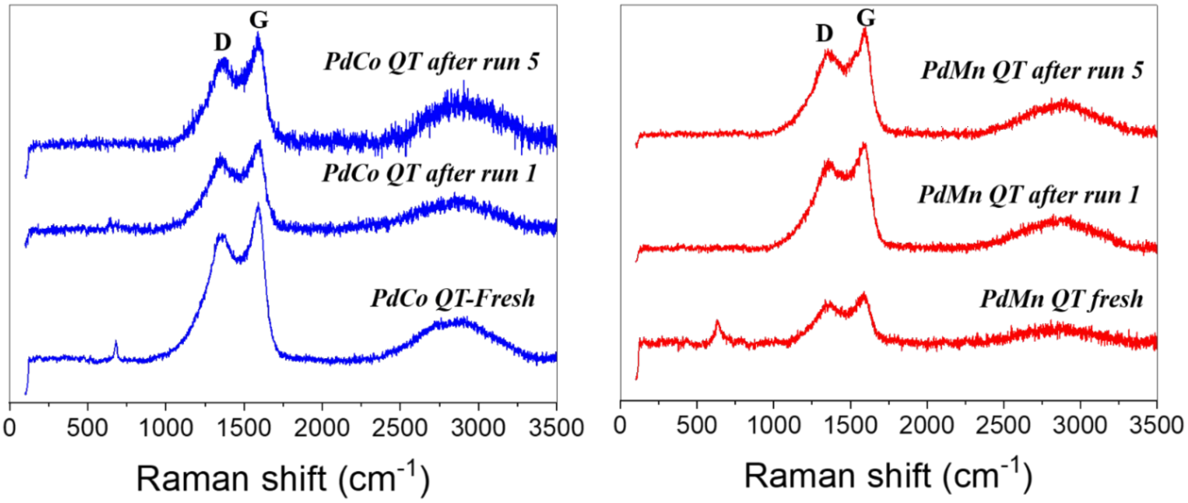

Figure S27. Raman spectra of **PdCo-QT** (blue), and **PdMn-QT** (red) materials (as prepared, after run 1 and after run 5).

Table S20. Detailed Raman shift of **PdCo-QT**, and **PdMn-QT** materials (as prepared, after run 1 and after run 5).

**Material**	**D band positions (cm^{-1})**	**G band positions (cm^{-1})**	**I_D/I_G**	**Material**	**D band positions (cm^{-1})**	**G band positions (cm^{-1})**	**I_D/I_G**
PdCo QT-fresh	**1357**	**1590**	**0.81**	***PdMn QT-fresh***	**1358**	**1586**	**0.82**
PdCo QT-run 1	**1351**	**1592**	**0.85**	***PdMn QT-run 1***	**1360**	**1589**	**0.83**
PdCo QT-run5	**1361**	**1592**	**0.80**	***PdMn QT-run5***	**1352**	**1592**	**0.80**

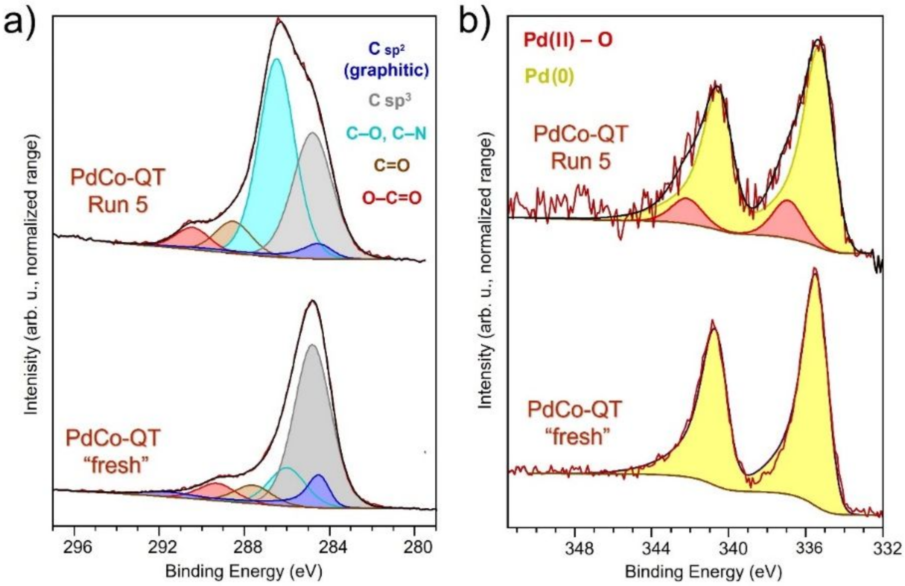

Figure S28. a) C*1s*, and b) Pd*3d* XPS regions of **PdCo-QT** samples (fresh and after run 5).

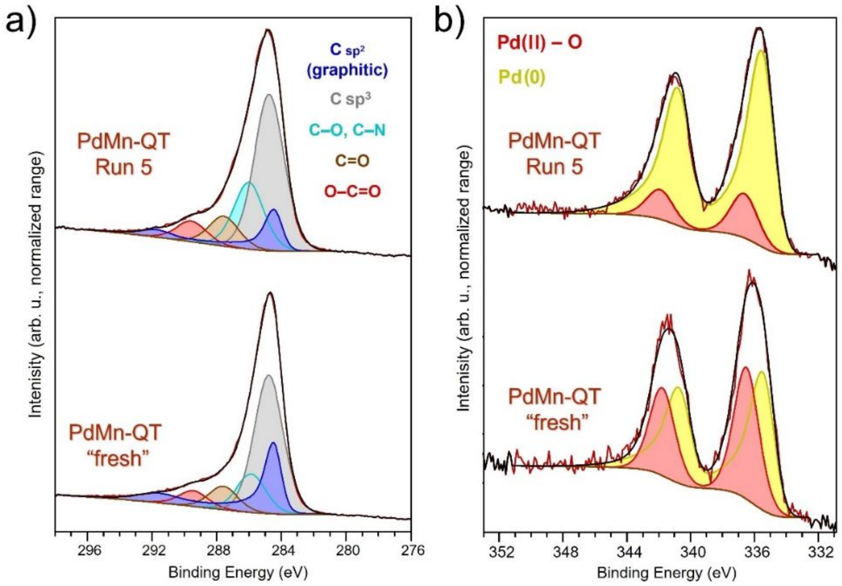

Figure S29. a) C*1s* and b) Pd*3d* XPS regions of **PdMn-QT** samples (fresh and after run 5).

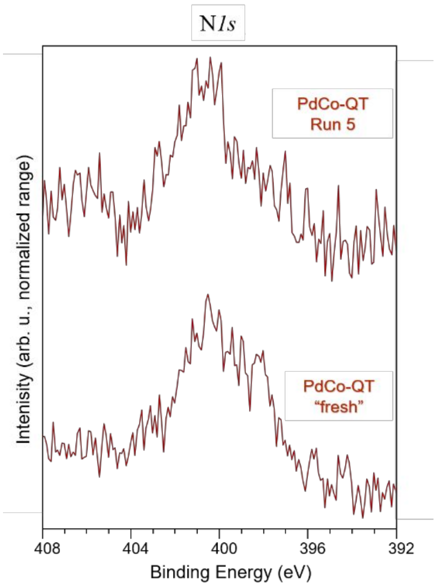

Figure S30. N*1s* XPS regions of **PdCo-QT** samples (fresh and after run5).

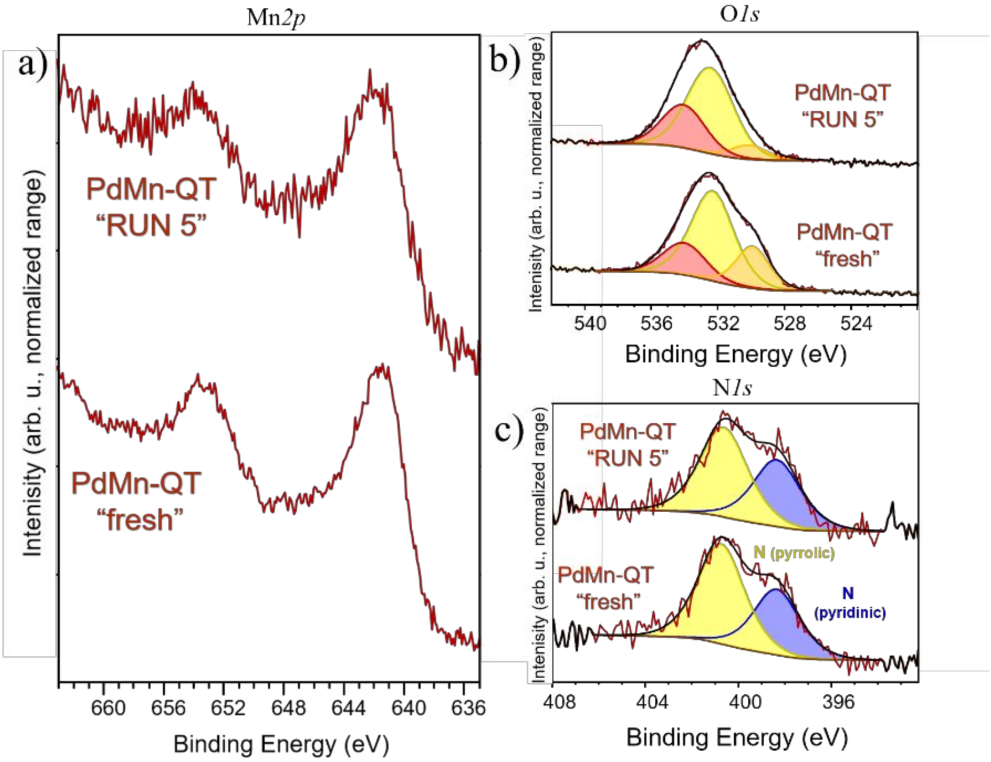

Figure S31. a) Mn*2p*, b) O*1s* and c) N*1s* XPS regions of **PdMn-QT** samples (fresh and after run 5).

III- References

(1) Militello, M. C.; Simko, S. J. Elemental Palladium by XPS. *Surf. Sci. Spectra* **1994**, *3* (4), 387–394.

(2) Morgan, D. J. Comments on the XPS Analysis of Carbon Materials. *C.* **2021**, *7* (3), 51.

(3) Bratsos, I.; Tampaxis, C.; Spanopoulos, I.; Demitri, N.; Charalambopoulou, G.; Vourloumis, D.; Steriotis, T. A.; Trikalitis, P. N. Heterometallic In(III)-Pd(II) Porous Metal-Organic Framework with Square-Octahedron Topology Displaying High CO2 Uptake and Selectivity toward CH4 and N2. *Inorg. Chem.* **2018**, *57* (12), 7244–7251.

(4) Martinez, J. S.; Mazarío, J.; Lopes, C. W.; Trasobares, S.; Calvino Gamez, J. J.; Agostini, G.; Oña-Burgos, P. Efficient Alkyne Semihydrogenation Catalysis Enabled by Synergistic Chemical and Thermal Modifications of a PdIn MOF. *ACS Catal.* **2024**, *14* (7), 4768–4785.

(5) Li, Z.; Deng, L.; Kinloch, I. A.; Young, R. J. Raman Spectroscopy of Carbon Materials and Their Composites: Graphene, Nanotubes and Fibres. *Prog. Mater. Sci.* **2023**, *135*, 101089.

(6) Beyreuther, E.; Grafström, S.; Eng, L. M.; Thiele, C.; Dörr, K. XPS Investigation of Mn Valence in Lanthanum Manganite Thin Films under Variation of Oxygen Content. *Phys. Rev. B - Condens. Matter Mater. Phys.* **2006**, *73* (15), 155425.

(7) Li, J.; Kou, J.; Xiang, Y.; Chen, M.; Zhang, J.; Zhan, X.; Zhang, H.; Wang, F.; Dong, Z. ZIF-8 Derived N-Doped Porous Carbon Confined Ultrafine PdNi Bimetallic Nanoparticles for Semi-Hydrogenation of Alkynes. *Mol. Catal.* **2023**, *535*, 112865.

(8) Song, X.; Shao, F.; Zhao, Z.; Li, X.; Wei, Z.; Wang, J. Single-Atom Ni-Modified Al2O3-Supported Pd for Mild-Temperature Semi-Hydrogenation of Alkynes. *ACS Catal.* **2022**, *12* (24), 14846–14855.

(9) Feng, Q.; Zhao, S.; Wang, Y.; Dong, J.; Chen, W.; He, D.; Wang, D.; Yang, J.; Zhu, Y.; Zhu, H.; Gu, L.; Li, Z.; Liu, Y.; Yu, R.; Li, J.; Li, Y. Isolated Single-Atom Pd Sites in Intermetallic Nanostructures: High Catalytic Selectivity for Semihydrogenation of Alkynes. *J. Am. Chem. Soc.* **2017**, *139* (21), 7294–7301.

(10) Liu, J.; Zhu, Y.; Liu, C.; Wang, X.; Cao, C.; Song, W.; Zhu, Y.; Liu, C.; Wang, X.; ao, D.; Song, W.; Liu, J. Excellent Selectivity with High Conversion in the Semihydrogenation of Alkynes Using Palladium-Based Bimetallic Catalysts. *ChemCatChem* **2017**, *9* (21), 4053–4057.

(11) Chen, M.; Kou, J.; Ma, H.; Xiang, Y.; Ma, P.; Sun, L.; Zhan, X.; Zhang, J.; Zhang, H.; Wang, F.; Dong, Z. Acceleration of the Semi-Hydrogenation of Alkynes over an N-Doped Porous Carbon Sphere-Confined Ultrafine PdCu Bimetallic Nanoparticle Catalyst. *Phys. Chem. Chem. Phys.* **2023**, *25* (5), 4201–4210.

(12) Wang, S.; Liu, T.; Zhu, Y.; Liu, X.; Luo, Q.; Zhu, M.; Ding, T.; Yao, T. Structure-Activity Relationship of Continuous Transformed Palladium Nanoclusters and Single Atoms for Selective Phenylacetylene Hydrogenation. *J. Phys. Chem. C* **2023**, *127* (12), 5911–5919.

(13) Liu, H.; Zhu, P.; Yang, D.; Zhong, C.; Li, J.; Liang, X.; Wang, L.; Yin, H.; Wang, D.; Li, Y. Pd-Mn/NC Dual Single-Atomic Sites with Hollow Mesopores for the Highly Efficient Semihydrogenation of Phenylacetylene. *J. Am. Chem. Soc.* **2024**, *146* (3), 2132–2140.

(14) Tang, J.; Jia, K.; Zhang, R.; Liu, C.; Lin, X.; Ge, T.; Liu, X.; Zhao, Q.; Liu, W.; Ma, D.; Fan, H.; Huang, J. Selective Hydrogenation of Alkyne by Atomically Precise Pd6 Nanocluster Catalysts: Accurate Construction of the Coplanar and Specific Active Sites. *ACS Catal.* **2024**, *14* (4), 2463–2472.
